# Castor Bean Organelle Genome Sequencing and Worldwide Genetic Diversity Analysis

**DOI:** 10.1371/journal.pone.0021743

**Published:** 2011-07-07

**Authors:** Maximo Rivarola, Jeffrey T. Foster, Agnes P. Chan, Amber L. Williams, Danny W. Rice, Xinyue Liu, Admasu Melake-Berhan, Heather Huot Creasy, Daniela Puiu, M. J. Rosovitz, Hoda M. Khouri, Stephen M. Beckstrom-Sternberg, Gerard J. Allan, Paul Keim, Jacques Ravel, Pablo D. Rabinowicz

**Affiliations:** 1 Institute for Genome Sciences, University of Maryland School of Medicine, Baltimore, Maryland, United States of America; 2 Center for Microbial Genetics and Genomics, Northern Arizona University, Flagstaff, Arizona, United States of America; 3 J. Craig Venter Institute, Rockville, Maryland, United States of America; 4 Department of Biological Sciences, Environmental Genetics and Genomics Laboratory, Northern Arizona University, Flagstaff, Arizona, United States of America; 5 Department of Biology, Indiana University, Bloomington, Indiana, United States of America; 6 Pathogen Genomics Division, Translational Genomics Research Institute, Phoenix, Arizona, United States of America; 7 Department of Microbiology and Immunology, University of Maryland School of Medicine, Baltimore, Maryland, United States of America; 8 Department of Biochemistry and Molecular Biology, University of Maryland School of Medicine, Baltimore, Maryland, United States of America; UCLA-DOE Institute for Genomics and Proteomics, United States of America

## Abstract

Castor bean is an important oil-producing plant in the Euphorbiaceae family. Its high-quality oil contains up to 90% of the unusual fatty acid ricinoleate, which has many industrial and medical applications. Castor bean seeds also contain ricin, a highly toxic Type 2 ribosome-inactivating protein, which has gained relevance in recent years due to biosafety concerns. In order to gain knowledge on global genetic diversity in castor bean and to ultimately help the development of breeding and forensic tools, we carried out an extensive chloroplast sequence diversity analysis. Taking advantage of the recently published genome sequence of castor bean, we assembled the chloroplast and mitochondrion genomes extracting selected reads from the available whole genome shotgun reads. Using the chloroplast reference genome we used the methylation filtration technique to readily obtain draft genome sequences of 7 geographically and genetically diverse castor bean accessions. These sequence data were used to identify single nucleotide polymorphism markers and phylogenetic analysis resulted in the identification of two major clades that were not apparent in previous population genetic studies using genetic markers derived from nuclear DNA. Two distinct sub-clades could be defined within each major clade and large-scale genotyping of castor bean populations worldwide confirmed previously observed low levels of genetic diversity and showed a broad geographic distribution of each sub-clade.

## Introduction

Castor bean (*Ricinus communis*) is an oilseed crop in the Euphorbiaceae family, which includes 245 genera comprising 6,300 species [1]. Other important members of this family include tropical crops such as cassava (*Manihot esculenta*) and rubber tree (*Hevea brasiliensis*), as well as ornamental poinsettias (*Euphorbia pulcherrima*), the invasive weed leafy spurge (*Euphorbia esula*), and the parasitic *Rafflesia* that has the largest flower in the plant kingdom [2].

Castor bean is cultivated in tropical and subtropical areas of the world for oil production as well as an ornamental. The world annual castor oil production in 2004–5 was around 0.5 million tons [3] and the U.S. is among the world largest importers of castor oil and its derivatives [4], [5]. Castor oil contains 90% of the unusual fatty acid ricinoleate (12-hydroxy-oleate), whose hydroxy group has unique chemical and physical properties that make castor oil a vital industrial raw material for numerous products [6], including high-quality lubricants, paints, coatings, plastics, soaps, medications for skin affections, and cosmetics. Castor oil can also be used as a lubricity additive to replace sulfur-based lubricant components in petroleum diesel helping to reduce sulfur emissions [7]. Moreover, Brazil, a highly advanced country in the use of ethanol as motor fuel, is starting to develop the industry of castor oil as a biodiesel component [8].

Castor bean seeds also contain ricin, a highly toxic water-soluble protein. Ricin is a Type 2 ribosome-inactivating enzyme [9] that can be extracted from seeds through a relatively simple process, raising concerns that it could be used as a bioweapon. This, added to its importance as an oil crop, poses a need to gain knowledge of the castor bean plant and its genetic variation in order to help the development of breeding and forensic tools. To this end, organelle genomic sequences can be used to develop robust markers for genetic and phylogenetic studies due to their high level of conservation.

The chloroplast genome contains genes that code for structural and functional components of the organelle. In land plants, chloroplast genomes are contained in a circular molecule of DNA ranging between 115 and 165 kb in size [10]. It contains two copies of a duplicated region arranged as inverted repeats (IR), separated by a large single-copy (LSC) sequence and a small single-copy (SSC) sequence [11], [12].

Complete chloroplast genomic sequences can be obtained using a shotgun strategy [13]. This approach involves DNA purification from isolated organelles [14], mechanical shearing of the DNA, and cloning of random fragments for sequencing [15], [16]. Cloned restriction fragments from purified chloroplast DNA have also been used to complete chloroplast genome sequencing [17], [18]. If a reference chloroplast genome from a related species is available, primers can be designed to amplify multiple PCR fragments to cover and assemble the whole chloroplast genome [19], [20]. Recently, the rolling circle amplification technique [21] has been used to amplify chloroplast DNA and generate the necessary quantities of DNA for standard Sanger capillary sequencing [22], [23] or next-generation sequencing [24]. In addition, chloroplast genomes are often assembled as byproducts of plant genome sequencing projects. Complete chloroplast genome sequences have been obtained using genomic bacterial artificial chromosome (BAC) libraries. In these cases, the libraries are screened by hybridization or PCR amplification of a conserved chloroplast sequence to identify large-insert BAC clones that contain chloroplast DNA. A few BAC clones that cover the complete chloroplast genome are then sequenced by a shotgun strategy [25].

Here we report the compete castor bean chloroplast and mitochondrion genome sequences generated from a whole genome shotgun (WGS) sequencing project of the cultivar (cv.) Hale [26] along with draft chloroplast genome sequences obtained by methylation filtration (MF) [27] from additional castor bean cultivars. MF is used for selectively cloning and sequencing hypomethylated (*i.e.* low-copy) sequences from plant genomes, reducing the recovery of repetitive elements that are usually methylated [28], [29], [30]. As the chloroplast DNA is not methylated [31], [32], MF libraries are typically constructed using nuclear DNA to reduce the recovery of mitochondrion and, mainly, chloroplast DNA clones, which are preferentially selected in MF libraries [33]. Therefore, we used a MF approach to sequence the chloroplast genomes of seven additional castor bean accessions in order to identify polymorphisms for phylogenetic and population genetic studies.

Castor bean has low genetic diversity based on AFLP and SSR studies [34], and recent single nucleotide polymorphism (SNP) analysis in a worldwide collection of accessions identified five main groups of castor bean showing a mixture of genotypes in most geographical regions studied [35]. The chloroplast SNPs analysis described here, contributes to the characterization of the genetic diversity and global castor bean population structure.

## Results and Discussion

### Assembly of the castor bean cv. Hale organelle genomes

Organelle genomic sequences were extracted from the castor bean cultivar Hale sequence database [26]. The chloroplast genome was assembled using the Celera Assembler [36] using short (3.5–9 kbp) and long (40 kbp) paired-end Sanger reads that showed high similarity to other dicot chloroplast genomes. The 40 kbp insert size fosmid reads allowed us to resolve the proper assembly and orientation of the large inverted repeat, common in angiosperm chloroplasts. This assembly was used to identify additional chloroplast sequences from a larger pool of castor bean sequences that were used to close the gaps in the draft assembly. The resulting closed circular molecule contained 163,161 bp with a near-perfect inverted repeat of 27,347 bp.

**Figure 1:**
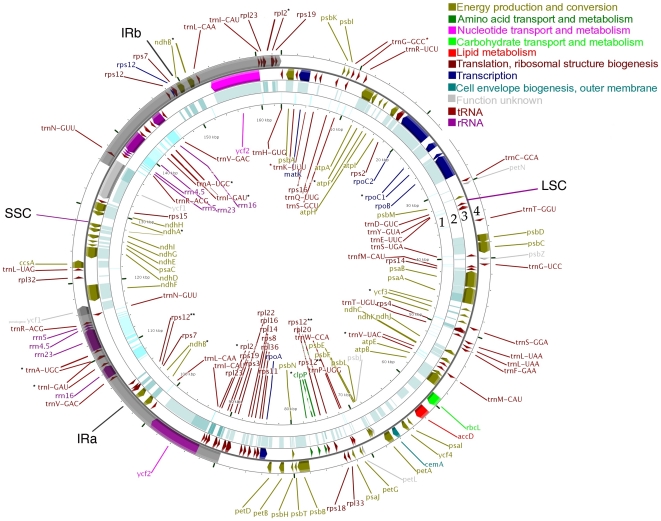
Map of the castor bean chloroplast. The large (LSC, 89,652 bp) and small (SSC, 18,817 bp) single copy regions are separated by two inverted repeats (IRa and IRb, 27,347 bp each), which are colored in grey. The outer most whorl (4) contains genes transcribed clockwise, and the genes on whorl 3 are transcribed counter-clockwise. The inner-most whorl (1) shows the alignment of a nucleotide blast of *R. communis* against all cassava (*M. esculenta*) tRNAs and rRNAs from the plastid genome. Whorl 2 shows the alignment of a nucleotide to protein blast (BLASTX) of *R. communis* against all proteins of the cassava plastid genome (*M. esculenta*). * Split genes or genes with introns. ** Trans-spliced gene *rps12*.

Annotation of the finished chloroplast molecule predicted 129 genes, including 38 tRNA genes, 8 ribosomal RNA genes, and 83 protein-coding genes (Figure 1). Each of the 27 kbp inverted repeats contains 17 genes. Fourteen genes contain one intron and 3 genes contain 2 introns. Cassava is the closest relative of castor bean whose chloroplast genome has been sequenced and annotated [37]. Sequence alignment of these two genomes showed a 93% sequence identity over approximately 150 of the 161 kbp of the castor bean chloroplast genome. The gene content of both chloroplasts is identical, and the presence of the conserved *atpF* group II intron in castor bean, which is absent in cassava [37], was confirmed. At the amino acid level, an average of 91% identity was observed between the castor bean and cassava protein-coding genes.

Sequence reads belonging to the mitochondrion genome were extracted using a custom script and assembled using the CAP3 assembler [38]. The assembled genome represents a consensus of the multiple possible genomic and subgenomic configurations that can exist in vivo [39]. The high confidence consensus mitochondrial genome spans 502,773 bp. Due to its larger size and lower abundance, the mitochondrial genome is less suitable for marker development in multiple cultivars than the chloroplast counterpart. Therefore, we did not further utilize the mitochondrion genome for genetic diversity analysis.

The mitochondrial genomes typically code for a few RNA genes as well as some ribosomal proteins and subunits of the oxidative phosphorylation complexes. The castor bean mitochondrial genome contains 37 protein-coding and 3 ribosomal RNA genes, commonly found in angiosperms (Figure S1). Horizontal gene transfer to the nuclear genome often results in variability regarding presence or absence of genes in the mitochondrial genome. In castor bean, the genes coding for ribosomal proteins L2 and S14, often found in plant mitochondrial genomes [40], have been transferred to the nuclear genome, while the gene coding for ribosomal protein L10, which has been recently found in the nuclear or mitochondrial genomes in different angiosperms [41], [42], has been retained in the castor bean mitochondrial genome.

### Sequencing the chloroplast genomes of additional castor bean accessions

Based on diversity groupings previously determined by AFLP analysis [34], we selected seven castor bean accessions representing genetically and geographically diverse varieties that originated in different parts of the world (Ethiopia, India, U.S. Virgin Islands, Puerto Rico, El Salvador, Greece, and Mexico) for chloroplast genome sequencing and subsequent SNP discovery.

**Figure 2:**
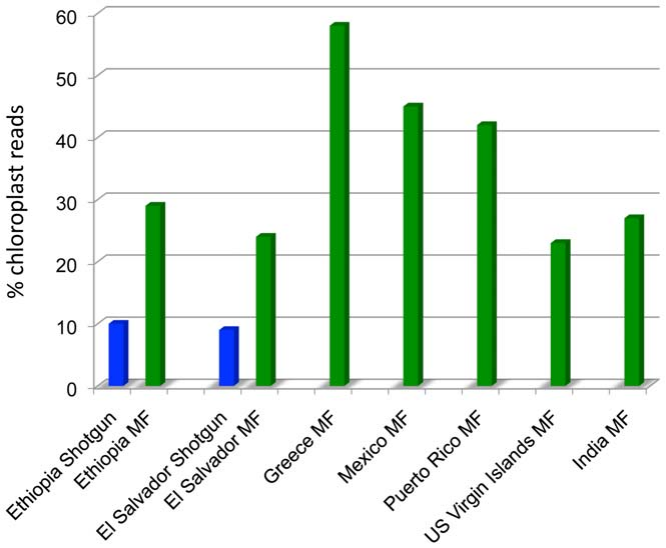
Chloroplast read enrichment in methylation filtration libraries. Percentage of chloroplast reads in each shotgun (blue) or MF (green) library. Available shotgun reads for cultivars from Ethiopia and El Salvador are shown to highlight the enrichment in chloroplast sequences in MF libraries.

In order to rapidly obtain chloroplast sequence information we constructed a MF library for each accession and sequenced both ends of several thousand clones per library (Table 1). To estimate the enrichment in chloroplast sequences obtained by MF of leaf genomic DNA from castor bean we also prepared standard shotgun genomic libraries for the accessions from Ethiopia and El Salvador. While the shotgun sequences produced 10% and 9% chloroplast sequences in these two accessions, respectively, the proportion of plastid sequences went up to 29% and 24%, respectively, (nearly a 3-fold enrichment) in the MF libraries. On average, the proportion of chloroplast sequences in the seven accessions' MF libraries was 35% with a maximum of 58% in the Greek accession (Figure 2).

**Table 1:** MF and WGS sequencing of 7 additional castor bean accessions.

Origin	Castor bean accession	Number of shotgun reads	% chloroplast shotgun reads	Number of MF reads	% chloroplast MF reads	Number of contigs
Ethiopia	PI 193851	9,216	10	2,304	29	2
El Salvador	PI 197048	192	9	7,104	24	21
Greece	PI 280219	N/A	N/A	4,032	58	3
Mexico	PI 255238	N/A	N/A	6,144	45	3
Puerto Rico	PI 209132	N/A	N/A	5,376	42	8
US Virgin Islands	PI 209326	N/A	N/A	6,912	23	15
India	PI 173946	N/A	N/A	5,376	27	38

**Figure 3:**
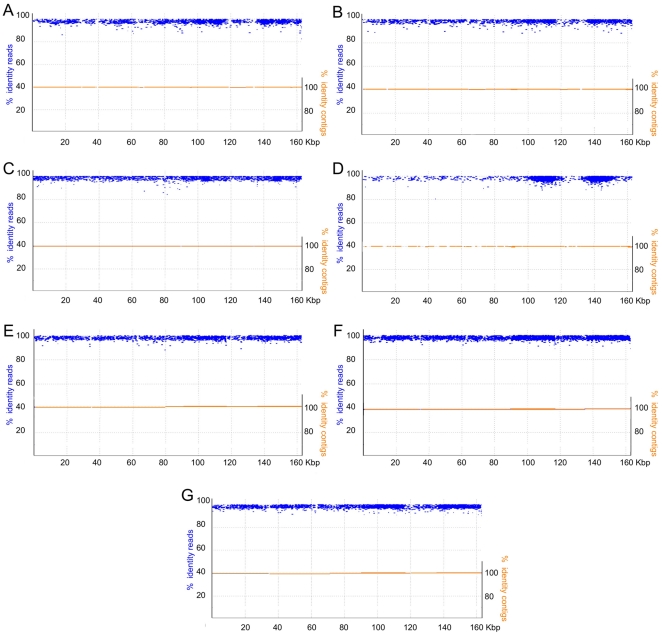
Draft assemblies of the chloroplast genomes of 7 castor bean accessions. Assembled contigs corresponding to the chloroplast genomes of the different accessions are shown in orange, mapped to the reference v. Hale chloroplast genome (x-axis). Individual reads are shown in blue, aligned to the same reference genome. Left y-axis corresponds to the percent identity of each read relative to the reference cv. Hale sequence. Right y-axis shows the percent identity of contig relative to the Hale sequence. Each panel corresponds to a different cultivar: A) US Virgin Islands, B) El Salvador, C) Puerto Rico, D) India, E) Ethiopia, F) Greece, and G) Mexico.

The MF (or the combination of MF and shotgun) reads were assembled for each accession using the AMOS-Cmp assembler, which uses a reference sequence to guide the assembly [43]. We used the closed chloroplast genome that we obtained from the cv. Hale as reference and generated a draft assembly for the chloroplast genome of each cultivar. In this way we were able to order and orient all contigs in each genome, producing a single scaffold for each one. In general, we obtained fairly complete assemblies that would allow SNP identification. Thus, we did not attempt to do any manual work to finish the sequences. Nevertheless, alignment of all the reads in each genome to the reference showed that most of the gaps in the assemblies could potentially be covered by existing sequence reads after manual analysis (Figure 3). In order to obtain genotypic information for some SNPs that fell within gaps in some of the genomes, we performed additional assemblies of the same sequences with the Celera assembler, which uses a different algorithm [36]. In this way we managed to close some gaps, increasing the chances of identifying SNPs with genotypic information in all seven cultivars.

Chloroplast DNA sequences are useful for phylogenetics and population genetics [44], [45], as well as cultivar identification and forensic analyses [46]. Our results show that MF is a cost-effective way to obtain sequence information of plant organelle genomes given that it does not require time-consuming plastid preparations, library screenings, or amplifications. With this approach, we were able to identify SNPs that allowed us to conduct a worldwide analysis of the castor bean accessions as described below.

### SNP identification and phylogenetic analysis

**Figure 4:**
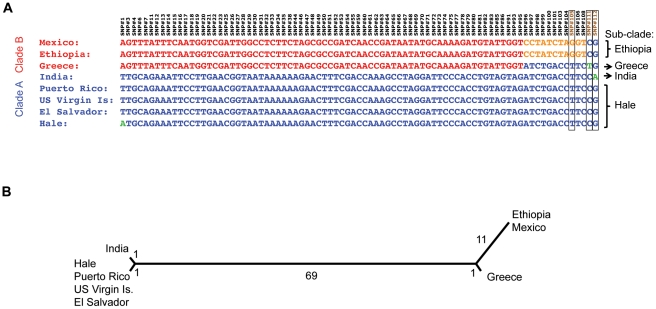
SNP identification and phylogeny obtained from genomic sequencing of 8 castor bean accessions. A) 83 high quality SNPs identified in the chloroplast genomes of the 8 sequenced chloroplast accessions. The two major clades are shown in red and blue and SNPs that differentiate different sub-clades are shown in orange or green. B) Phylogeny of the 8 castor bean accessions based on the identified SNPs. Two major clades, “A” and “B”, are shown separated by 69 SNPs. Members of each sub-clade are indicated in each branch of the phylogeny.

We searched for candidate SNPs in pair-wise alignments between each of the seven castor bean chloroplast genome assemblies and that of cv. Hale. Using custom bioinformatics analyses and stringent criteria (see Methods), 112 SNPs were identified (Table S1). One of them showed a triallelic nature so it was discarded and 29 were not covered in all seven chloroplast genome assemblies. Of these 29, SNP#111 was covered in all genome assemblies except that of the Indian cv. However, because the Greek cv. showed a rare allele for this SNP and was specific to this cultivar (see below) we genotyped this SNP using a TaqMan assay in the Indian cv. to verify that the Greek cv. allele was not present in any of the other genomes. As a result we selected a set of 83 SNPs for which there is high quality sequence data for all cultivars (including SNP#111, Figure 4A) and we used this SNP set to construct a chloroplast-based phylogeny (Figure 4B). Two main groups were evident among the seven castor bean cultivars and we called them clades A and B, which are separated by 69 of the 83 high-quality SNPs.

Members of clade A are highly similar, with only SNP#112 unique to the Indian accession (designating the India sub-clade). The accessions from Puerto Rico, Virgin Islands, and El Salvador were monomorphic for all SNPs and differed from cv. Hale only at SNP#1 (Figure 4A). However, because no assay could be designed for this SNP, these four cultivars are hereafter referred to as the Hale sub-clade. For clade B, the Ethiopian and Mexican accessions did not show polymorphisms for any of the SNPs analyzed and we refer to these accessions as the Ethiopia sub-clade as defined by SNP#105. SNP#111 allowed the identification of the Greek cv. genotype (Greece sub-clade). Thus, the eight accessions with chloroplast sequences were divisible into four sub-clades (Hale, India, Greece, and Ethiopia). We designed TaqMan assays specific to these sub-clades in order to distinguish individuals belonging to each sub-clade among 894 castor bean samples from accessions collected around the world.

**Figure 5:**
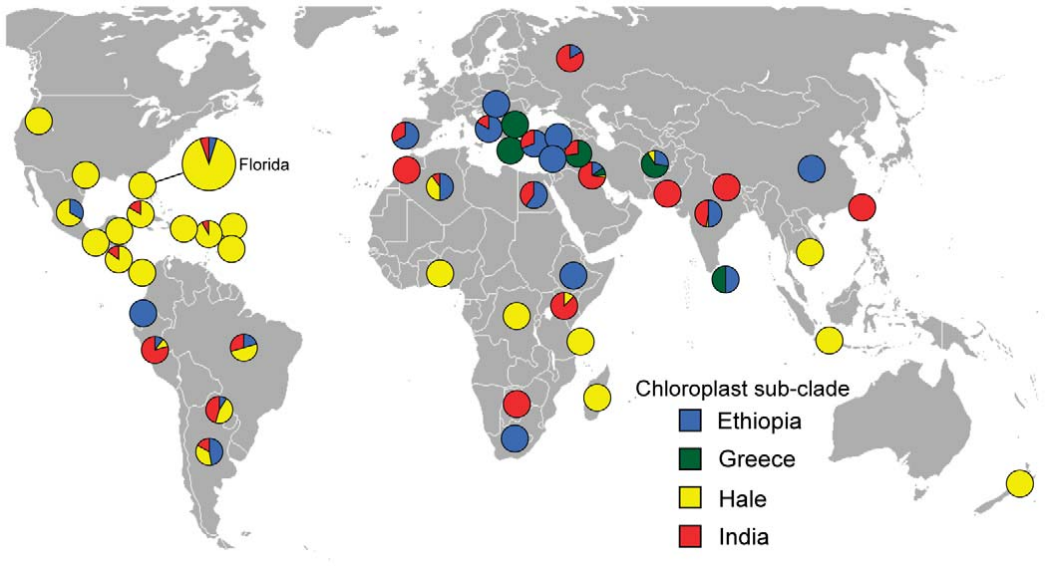
Worldwide distribution of chloroplast genotypes of castor bean from a collection of 894 accessions. Distribution of chloroplast genotypes corresponding to the sub-clades shown in Figure 4, based on the origin of each accession. The pie chart corresponding to Florida is expanded to reflect the larger number of samples (*n* = 272) that came from that state, relative to other parts of the world.

Overall, genotyping of the worldwide population with these assays showed that limited chloroplast genetic diversity exists throughout the world in castor bean, which is consistent with the low genetic variation observed for nuclear genomic sequences [34], [35]. Nevertheless, we could determine that each sub-clade was broadly spread geographically (Figure 5). Within clade A, the Hale sub-clade genotype was distributed throughout the world, dominating in collections from North and Central America, the Caribbean, and parts of South America, Africa, and Asia (Table 2). Members of the India sub-clade, within clade A as well, were also found widespread on every continent sampled. Members of the clade B were found less frequently than those of clade A, with the Ethiopia sub-clade being more widely distributed while the Greece sub-clade was mostly limited to the Middle East and Sri Lanka. The geographic origin of the different accessions could not always be correlated with its genotype. For example, the Mexican and Ethiopian cvs. showed no polymorphisms for any of the SNPs we tested, although the majority of samples from Mexico fell in the Hale sub-clade. A similar scenario was found in clade A, within which there is very little variation but included the Indian cv. together with mostly American varieties.

**Table 2:** Country or state of origin and the chloroplast sub-clade for 894 *Ricinus communis* samples collected worldwide.

	Number of samples per sub-clade			
Origin	Ethiopia	Greece	Hale	India
Afghanistan	3	7	1	
Algeria	5		4	1
Argentina	25		19	9
Bahamas			8	
Benin			8	
Botswana				11
Brazil	9		22	13
Cambodia			8	
China	5			
Costa Rica			5	1
Cuba			17	3
Ecuador	4			
Egypt	3			2
El Salvador			7	
Ethiopia	4			
Florida	13		244	15
Greece		3		
Guatemala			8	
Hale			5	
Hungary		4		
India	52		3	49
Indonesia			8	
Iran	5	3	1	24
Israel	5			
Jamaica			11	1
Jordan	5			
Kenya			1	7
Madagascar			8	
Mexico	3		6	
Morocco				5
Nepal				8
New Zealand			1	
Oregon			3	
Pakistan				6
Panama			8	
Paraguay	1		5	5
Peru	3		3	23
Portugal	4			2
Puerto Rico			8	
Russia	1			5
Serbia	5			1
South Africa	4			
Sri Lanka	1	1		
Syria		8		3
Taiwan				2
Texas			1	
Turkey	40			18
Uruguay			11	
US Virgin Islands			8	
Yugoslavia	5			
Zaire			5	
Zanzibar			2	
Total	205	26	449	214

The two main phylogenetic groups obtained based on chloroplast DNA sequence variation argue for two ancient and differentiated populations that merged to generate the current global populations. In fact, two robust ancestral populations appear to have been involved in the creation of modern hybrid *Ricinus* populations —perhaps even different subspecies. This division into two groups was much less apparent from previous analysis of nuclear DNA sequence variation data [34], [35], likely because of extensive recombination. This has not happened in the cytoplasmic DNAs due to the lack of recombination. It is somewhat fortuitous that the two main groups still exist since cytoplasmic DNA would tend to rapidly go to fixation as one type or the other, especially in small populations.

We compared the nuclear SNP data where five main groups where encountered worldwide [35] with the four chloroplast sub-clades. A strong relationship occurred between groupings based on nuclear and chloroplast SNPs, particularly for the Hale cultivar genotype. Because 451 of the samples that we analyzed in this study had also been analyzed with nuclear SNPs [35], we could compare the grouping results from both types of SNP markers for exactly the same samples. For this subset of samples, 96.1% (171 of 178) of the Hale chloroplast sub-clade genotypes were also grouped together when analyzed with nuclear SNPs. This correspondence was not complete for comparisons between other groups and their most likely population assignment: India 68% (34 of 50), Ethiopia 57% (59 of 86), and Greece 8% (11 of 137).

Global patterns of genetic variation in castor bean have likely been influenced by agricultural and horticultural practices. The pattern of introduction of *R. communis* from multiple chloroplast groupings in many countries is consistent with genetic patterns from nuclear SNPs [35]. In some areas however, such as North America, this is not evident from chloroplast DNA probably because of the limited diversity found in the chloroplast genome. We caution that this low level of genetic diversity may be related to the fact that our samples were limited primarily to those available from the U.S. Department of Agriculture (USDA) germplasm collection, which may not represent the totality of the world's geographic and genetic diversity for castor bean.

## Materials and Methods

### Plant material

Castor bean accessions were obtained from USDA's National Plant Germplasm System, Agricultural Research Center in Griffin, Georgia and some of the accessions for the worldwide population analysis were also obtained from commercial sources or collected in the wild in the state of Florida, USA. The accessions selected for chloroplast genome sequencing were: Ethiopian cv.: PI193851, Greek cv.: PI280219, Mexican cv.: PI255238, Puerto Rican cv.: PI209132, Indian cv.: PI173946, Salvadorian cv.: PI197048, and cv. from U.S. Virgin Islands: PI209326. Plants were grown in normal greenhouse conditions and leaf samples were collected and dried before DNA extraction as described [34]. Total DNA was prepared using DNeasy kits (Qiagen) following the manufacturer's instructions.

### Organelle genome sequencing, assembly and annotation

Assembly of the castor bean cv. Hale chloroplast genome was carried out by selecting chloroplast reads from the database of whole genome shotgun Sanger reads generated for genome assembly [26]. This database includes paired-end sequence reads from multiple plasmid and fosmid libraries. From this database, we extracted a set of approximately 7,000 3–4 kbp-insert paired-end plasmid reads and selected 765 reads that could be aligned at high stringency against available dicot chloroplast genome sequences from GenBank using sequence similarity searches. These reads were assembled using the Celera Assembler [36]. Subsequently, additional chloroplast reads from fosmid clones were added to the assembly to allow building and orienting the 27 kbp inverted repeats. We then performed standard finishing work to close sequence gaps and resolve ambiguities [47].

Because the sequence and structure of angiosperm mitochondrial genomes are poorly conserved and include unique sequences, it is relatively difficult to identify all reads corresponding to the mitochondrial genome when mixed with a nuclear genome WGS project. To address this difficulty, we developed a method for extending initially assembled reads by iteratively comparing all reads to the ends of the extending assemblies. By examining the read depths of the resulting assemblies, mitochondrial sequences could be separated from those of nuclear and chloroplast origin. The resulting reads were assembled using CAP3 [38].

The chloroplast genomes of the seven additional castor bean cultivars were sequenced using MF libraries. Briefly, castor bean total DNA was purified from leaves and was randomly sheared by nebulization, end-repaired with consecutive BAL31 nuclease and T4 DNA polymerase treatments, and 1.5 to 3 kb fragments were eluted from a 1% low-melting-point agarose gel after electrophoresis. After ligation to BstXI adaptors, DNA was purified by three rounds of gel electrophoresis to remove excess adaptors, and the fragments were ligated into the vector pHOS2 (a modified pBR322 vector) linearized with BstXI. The pHOS2 plasmid contains two BstXI cloning sites immediately flanked by sequencing primer binding sites. The ligation reactions were introduced by electroporation into *E. coli* strain GC10 (*McrBC* ^−^) for regular shotgun libraries or strain DH5α (*McrBC* ^+^) for MF libraries.

Draft assemblies of the genomes of the seven castor bean cultivars sequenced were conducted using AMOS-Cmp [43] using the closed cv. Hale genome sequence as reference. Additional assemblies of the same sequence data were performed with the Celera assembler [36] in order to cover some of the gaps that contained candidate SNPs.

Chloroplast genome annotation was carried out using the software tool DOGMA (Dual Organellar GenoMe Annotator) [48] followed by manual inspection of intron-containing genes. Comparative analysis of castor bean and cassava chloroplast genomes was carried out using CGView [49]. The mitochondrion genome was annotated using comparative analysis to identify mitochondrial genes, taking into account exons and introns. Intergenic regions were further annotated as possible using sequence similarity searches against GenBank.

The finished and annotated castor bean chloroplast and mitochondrion genome sequences from the cv. Hale can be found under GenBank accession numbers JF937588 and HQ874649, respectively. The draft sequence assemblies of the 7 castor bean accessions chloroplast genomes can be found under GenBank accession numbers JF940518 (Ethiopia), JF940520 (India), JF940515 (U.S. Virgin Islands), JF940521 (Puerto Rico), JF940517 (El Salvador), JF940519 (Greece), and JF940516 (Mexico).

### SNP discovery

In order to call candidate SNPs in pair-wise comparisons of the seven castor bean chloroplast genomes versus the Hale cv. genome we applied the following criteria: reads must be longer than 500 bp, the polymorphic base should be covered by at least 3 reads and should not be located in the first or last 30 bp of any read, the Phred quality score of the polymorphic base and mean scores of the 5 bases upstream and 5 bases downstream should be greater than 30, and the polymorphic base should be covered in all cultivars meeting the previous criteria.

### Genotyping assay development and screening

We developed three Real-Time TaqMan (Applied Biosystems) minor groove binding (MGB) assays for the four castor bean sub-clades. Primers and probes were designed using Primer Express for TaqMan MGB assays for Allelic Discrimination version 3.0 (Applied Biosystems). Each assay had dual-probes targeting the specific SNP (Table 3). Although a single SNP separates the Hale cultivar from the cultivars from Puerto Rico, U.S. Virgin Islands, and El Salvador, sequence composition prevented the development of a reliable assay, so these closely related cultivars were grouped together.

**Table 3:** Sequence for TaqMan primers and probes for defining castor bean chloroplast sub-clades.

SNP	Sub-clade	Forward primer sequence	Reverse primer sequence	Sub-clade specific probe*	Alternate probe*
105	Ethiopia	GACATTCCGTCTTCTGAAACCAA	CTAACTATAGTGCAAAGTCGCATCTCTT	TTTCTATATGCCGATTATGG	TTTCTATATGCCTATTATG
111	Greece	GATTGATTGGCTGATGTTTCAAAA	AAAGAAACGTCTGTATTCAGCTACAAAG	ATACCCAAAGTTCC	ATACCCAAAGCTCCCA
112	India	TGTCAGGCTATTGTTCTCCTGTTC	GGGAGTCCATCATGTAATCAAAAGA	CTAAAAGTAATGAAGTAAGAC	AAGTAATGGAGTAAGACATC

*SNP-state is underlined in each probe.

We ran the genotyping assays on an ABI Prism 7900HT Sequence Detection System (Applied Biosystems). Each 10 µl PCR mixture contained 1× AB TaqMan Universal Master mix, 0.9 µM each primer, and 0.25 µM each probe. We added 0.2 U of Platinum Taq (Invitrogen) per reaction to increase the efficiency of the amplification. Each assay was run at standard conditions: 2 min inactivation at 50°C, a 10 min hot start at 95°C, followed by 40 cycles of 15 sec denaturation and 1 min annealing at 60°C. Amplification and allelic discrimination plots were visualized using SDS version 2.3 software. We screened 894 samples from 49 countries and 3 US states against all three SNP assays.

## Supporting Information

Figure S1 **The mitochondrion genome.** Circular map of the castor bean mitochondrial genome generated using OrganellarGenomeDRAW [Lohse M, Drechsel O, Bock R (2007) Curr Genet 52: 267–274]. Genes inside of the circle are transcribed clockwise, and genes outside the circle are transcribed counterclockwise. The GC content graph is shown on inner ring (dark on light).
(PDF)

Table S1 **SNPs identified in the castor bean chloroplast genomes.** Flanking sequences for each SNP is given, noting the polymorphic base between brackets.
(DOC)
