# Supplementary material for: Castor Bean Organelle Genome Sequencing and Worldwide Genetic Diversity Analysis
Source: PLoS One. 2011 Jul 7;6(7):e21743. doi: 10.1371/journal.pone.0021743 (PMC3131294; doi:10.1371/journal.pone.0021743)
Supplement: Figure S1 — The mitochondrion genome. Circular map of the castor bean mitochondrial genome generated using OrganellarGenomeDRAW [Lohse M, Drechsel O, Bock R (2007) Curr Genet 52: 267–274]. Genes inside of the circle are transcribed clockwise, and genes outside the circle are transcribed counterclockwise. The GC content graph is shown on inner ring (dark on light). (PDF) [file pone.0021743.s001.pdf]

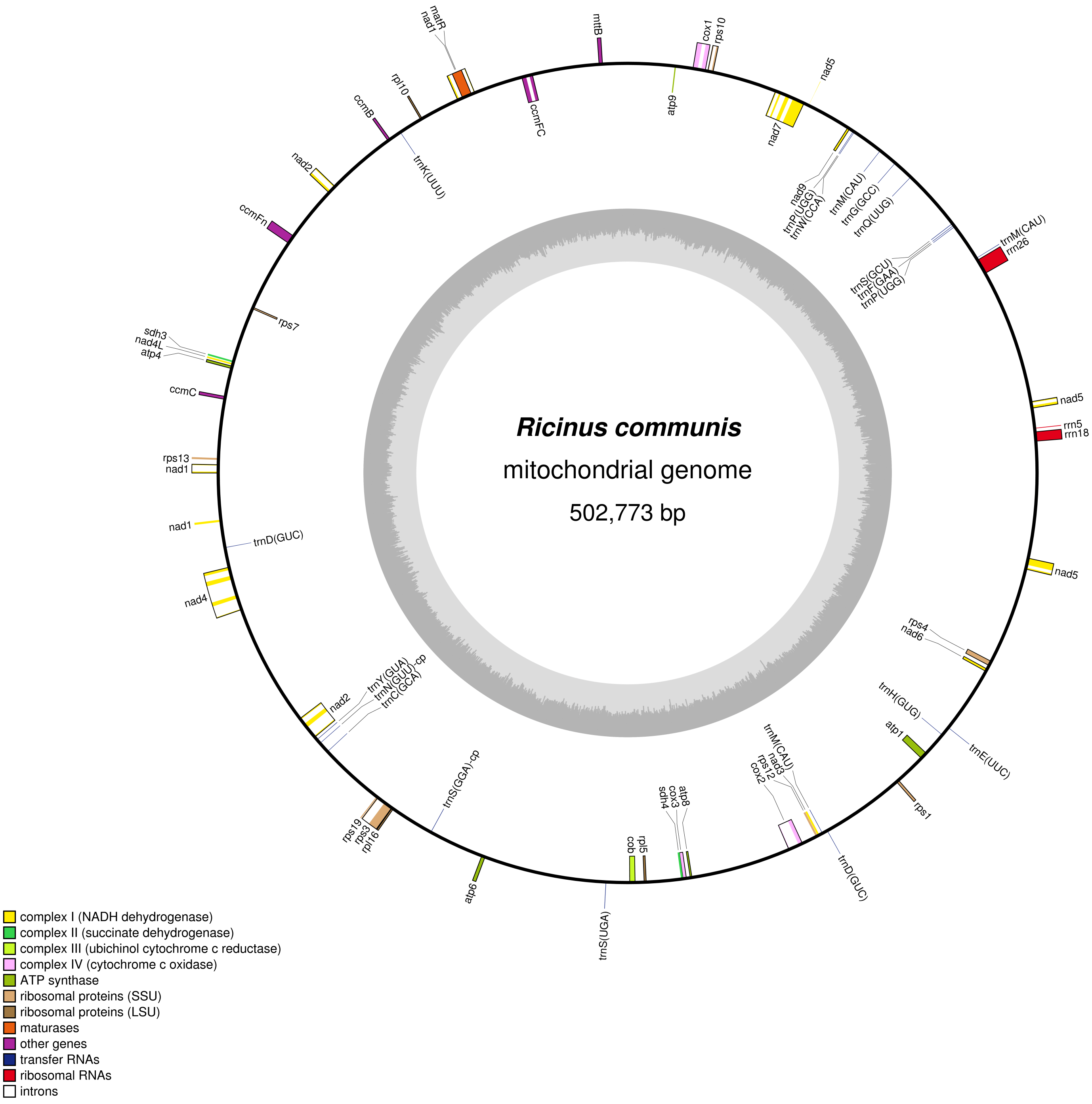
Ricinus communis
mitochondrial genome
502,773 bp
matR
nad1
mttB
cox1
rps10
nad5
atp9
nad7
ccmFC
rpl10
ccmB
trnK(UUU)
nad2
ccmFn
nad9
trnP(UGG)
trnW(CCA)
trnM(CAU)
trnG(GCC)
trnQ(UUG)
trnM(CAU)
rrn26
trnS(GCU)
trnF(GAA)
trnP(UGG)
rps7
sdh3
nad4L
atp4
ccmC
nad5
rrn5
rrn18
rps13
nad1
nad1
trnD(GUC)
nad5
nad4
rps4
nad6
trnH(GUG)
atp1
trnE(UUC)
nad2
trnY(GUA)
trnN(GUU)-cp
trnC(GCA)
trnM(CAU)
nad3
rps12
cox2
rps1
atp8
cox3
sdh4
rpl5
cob
trnS(GGA)-cp
rps19
rps3
rpl16
atp6
trnS(UGA)
trnD(GUC)
complex I (NADH dehydrogenase)
complex II (succinate dehydrogenase)
complex III (ubichinol cytochrome c reductase)
complex IV (cytochrome c oxidase)
ATP synthase
ribosomal proteins (SSU)
ribosomal proteins (LSU)
maturases
other genes
transfer RNAs
ribosomal RNAs
introns
