# Supplementary material for: Castor Bean Organelle Genome Sequencing and Worldwide Genetic Diversity Analysis
Source: PLoS One. 2011 Jul 7;6(7):e21743. doi: 10.1371/journal.pone.0021743 (PMC3131294; doi:10.1371/journal.pone.0021743)
Supplement: Table S1 — SNPs identified in the castor bean chloroplast genomes. Flanking sequences for each SNP is given, noting the polymorphic base between brackets. (DOC) [file pone.0021743.s002.doc]

Table S1

| **SNP number** | **Alleles** | **Coordinate in the Hale genome** | **Sequence flanking the SNPs (polymorphic base showing both alleles between brackets)** |
| --- | --- | --- | --- |
| SNP1 | A/T | 63537 | TTTCATGTTCTTTTCTTACCTATGCATAGAAAATATAGAGTCTTGCATATTTATATATCTCCTGGGAATCCCTTTTTTTTTACTAAAAAAAAATCGACGGATTCTAAATTATAACGAATTTTGGGGGAATTTATAATCGGAAAAACTCTTTATTTTTTTTTTTATTTTATATTAAATATATTAAAATATTTTCTATTTTCTATTAAAGTAAAGTTAAAAAAATGAAAAAAGAAAAATGAAAGTTATAAGACAAGAAAAAAAAAGATAATAAAAGAAGAATAACAAATAAATGGATTATCATATTTCATGTAGAAATATGAATAAGTCATTTAGTTAGTTCTACGCTCTTTGCACTTTATTATATTATATATACTCACTTAGATATACTTAGTATAATTATATACGAATTTTAATGATTATAAGAAATCTTTCTAATAACTAAATAACTAATAAATAACTAATAATAGAATAATTAATATAAATATAATTATAATTAATTT[A/T]AATTATTAATTTAAATTATTAAATTAATAGAATAAAATAATAATTAATAATAAAAAAAAAATAATTATAATAATAATAAATAATAATAAAAAAAAAAAAATAATGAAAATATATAAATATATTAAAATATAAATATATTAAATATATATTAAAATATATTAAATATATATTAAAATATTCAAAATAATATAATATAAAAATAATATAATTATAATATATAATAAAAAAAAAAAAATGAAATAACAGGTACAAATATTAAATCGAGGTGCCCATTCTATGACAATTCTCAACAGCTTACCCTCCATTTTTGTGCCTTTAGTAGGCTTAGTATTTCCGGCAATTGCGATGGCTTCCTTATCTCTTCATGTTCAAAAAAACAAGATTTTTTAGATCCAATTAGGTCTAACGGGGGTAGATTTCATTTCTTTTTTTTTTCAAGACTTAGACTTGGATCATAATATAATACAGATATAATACGGATATCTCTTTAGTTTAGTATA |
| SNP2 | A/G | 146 | GGGCGAATGACGGGAATTGAACCCGCGCATGGTGGATTCACAATCCACTGCCTTGATCCACTTGGCTACATCCGCCCCTAATCCTATAACTCTATTTAAAAATAAATATTCGATTAAATATTAGAAGTAATTAAATATTAGAAGT[A/G]GAAGGGTTTCAATTTCTAGCATTTAAATTTCAATTTTATCTCTCTTAAAAGATTTTATAAGATACAAATACAAGAATAAAATAAAACTACAAAAATAAAAAGACATAAAAATAAAAGCTTTATCTTTTATATCTTTTATTTTCAATCGAAAAAAAAAGAACTTCATAAAAAAAAAAGATTGAGAAGAACATATACTCAATTTAAAATAAGTACATAAACTATATATATAATCTTCAGAAAGCGACCCATAAAAAGATTTTTTTTTCTAAAAGATTCTTTTATTTTTTAATTTTAAAGATTAAAGAAAATAAAAAAAGAAAATAAACTTTTTTATATGTTTTTTTTATGTTTATGTAAAATAAACATATATATATTACTAAAATATAATTACTAAAAAAAGGAGCAATACCAATCCTCTCGATAGAACAAGAAATTGGCTATTGCTCCTTTTTTTATTTTATTTTCAAGAACTCACGCATACTAAGACCAAAGTCTTATCC |
| SNP3 | T/G | 312 | GGGCGAATGACGGGAATTGAACCCGCGCATGGTGGATTCACAATCCACTGCCTTGATCCACTTGGCTACATCCGCCCCTAATCCTATAACTCTATTTAAAAATAAATATTCGATTAAATATTAGAAGTAATTAAATATTAGAAGTAGAAGGGTTTCAATTTCTAGCATTTAAATTTCAATTTTATCTCTCTTAAAAGATTTTATAAGATACAAATACAAGAATAAAATAAAACTACAAAAATAAAAAGACATAAAAATAAAAGCTTTATCTTTTATATCTTTTATTTTCAATCGAAAAAAAAAGAACTTCA[T/G]AAAAAAAAAAGATTGAGAAGAACATATACTCAATTTAAAATAAGTACATAAACTATATATATAATCTTCAGAAAGCGACCCATAAAAAGATTTTTTTTTCTAAAAGATTCTTTTATTTTTTAATTTTAAAGATTAAAGAAAATAAAAAAAGAAAATAAACTTTTTTATATGTTTTTTTTATGTTTATGTAAAATAAACATATATATATTACTAAAATATAATTACTAAAAAAAGGAGCAATACCAATCCTCTCGATAGAACAAGAAATTGGCTATTGCTCCTTTTTTTATTTTATTTTCAAGAACTCACGCATACTAAGACCAAAGTCTTATCCATTTGTAGATGGAGCTTCGACAGCAGCTAGGTCTAGAGGGAAGTTATGAGCATTACGTTCATGCATAACTTCCATACCAAGGTTAGCACGGTTGATAATATCAGCCCAGGTATTAATTACACGACCTTGACTATCAACTACAGATTGGTTGAAATTGAAACCATTT |
| SNP4 | G/T | 463 | GGGCGAATGACGGGAATTGAACCCGCGCATGGTGGATTCACAATCCACTGCCTTGATCCACTTGGCTACATCCGCCCCTAATCCTATAACTCTATTTAAAAATAAATATTCGATTAAATATTAGAAGTAATTAAATATTAGAAGTAGAAGGGTTTCAATTTCTAGCATTTAAATTTCAATTTTATCTCTCTTAAAAGATTTTATAAGATACAAATACAAGAATAAAATAAAACTACAAAAATAAAAAGACATAAAAATAAAAGCTTTATCTTTTATATCTTTTATTTTCAATCGAAAAAAAAAGAACTTCATAAAAAAAAAAGATTGAGAAGAACATATACTCAATTTAAAATAAGTACATAAACTATATATATAATCTTCAGAAAGCGACCCATAAAAAGATTTTTTTTTCTAAAAGATTCTTTTATTTTTTAATTTTAAAGATTAAAGAAAATAAAAAAA[G/T]AAAATAAACTTTTTTATATGTTTTTTTTATGTTTATGTAAAATAAACATATATATATTACTAAAATATAATTACTAAAAAAAGGAGCAATACCAATCCTCTCGATAGAACAAGAAATTGGCTATTGCTCCTTTTTTTATTTTATTTTCAAGAACTCACGCATACTAAGACCAAAGTCTTATCCATTTGTAGATGGAGCTTCGACAGCAGCTAGGTCTAGAGGGAAGTTATGAGCATTACGTTCATGCATAACTTCCATACCAAGGTTAGCACGGTTGATAATATCAGCCCAGGTATTAATTACACGACCTTGACTATCAACTACAGATTGGTTGAAATTGAAACCATTTAGGTTGAAAGCCATAGTGCTAATACCTAAAGCAGTGAACCAAATACCTACTACAGGCCAAGCAGCTAGGAAGAAGTGTAAAGAACGAGAGTTGTTGAAACTAGCATATTGGAAGATCAATCGGCCAAAATAACCATGAGCAGCTACGATAT |
| SNP5 | C/T | 1205 | TCATGCATAACTTCCATACCAAGGTTAGCACGGTTGATAATATCAGCCCAGGTATTAATTACACGACCTTGACTATCAACTACAGATTGGTTGAAATTGAAACCATTTAGGTTGAAAGCCATAGTGCTAATACCTAAAGCAGTGAACCAAATACCTACTACAGGCCAAGCAGCTAGGAAGAAGTGTAAAGAACGAGAGTTGTTGAAACTAGCATATTGGAAGATCAATCGGCCAAAATAACCATGAGCAGCTACGATATTATAAGTTTCTTCCTCTTGACCGAATCTATAACCTTCATTAGCAGATTCATTTTCTGTGGTTTCCCTGATCAAACTAGAGGTTACCAAGGAACCATGCATAGCACTGAATAGGGAGCCGCCGAATACACCAGCTACGCCTAACATATGAAATGGGTGCATAAGGATGTTGTGTTCAGCCTGGAATACAATCATAAAGTTGAAAGTACCAGAAATTCCTAGAGGCATACCATCAGAAAAACT[C/T]CCTTGACCGATTGGATAGATCAAGAAAACAGCAGTAGCAGCTGCAACAGGAGCTGAATATGCAACAGCAATCCAAGGGCGCATACCCAGACGGAAACTAAGCTCCCACTCACGGCCCATGTAACAAGCTACACCAAGTAAGAAGTGTAGAACAATTAGCTCATAAGGACCGCCATTGTATAACCATTCATCAACAGACGCCGCTTCCCATATTGGGTAAAAATGCAAACCTATAGCTGCAGAAGTAGGAATAATGGCACCAGAAATAATATTGTTTCCATAAAGTAAAGATCCAGAAACAGGTTCACGAATACCATCAATATCTACCGGAGGGGCAGCAATGAAAGCGATAATAAATACAGAAGTTGCGGTCAATAAAGTTGGGATCATCAAAACACCAAACCATCCAATGTAAAGACGGTTTTCAGTGCTGGTTATCCAGTTACAGAAACGACCCCATAAGCTTTCGCTTTCGCGTCTCTCTAAAATTGCAGTCATGGT |
| SNP6 | C/T | 1307 | CCATTTAGGTTGAAAGCCATAGTGCTAATACCTAAAGCAGTGAACCAAATACCTACTACAGGCCAAGCAGCTAGGAAGAAGTGTAAAGAACGAGAGTTGTTGAAACTAGCATATTGGAAGATCAATCGGCCAAAATAACCATGAGCAGCTACGATATTATAAGTTTCTTCCTCTTGACCGAATCTATAACCTTCATTAGCAGATTCATTTTCTGTGGTTTCCCTGATCAAACTAGAGGTTACCAAGGAACCATGCATAGCACTGAATAGGGAGCCGCCGAATACACCAGCTACGCCTAACATATGAAATGGGTGCATAAGGATGTTGTGTTCAGCCTGGAATACAATCATAAAGTTGAAAGTACCAGAAATTCCTAGAGGCATACCATCAGAAAAACTCCCTTGACCGATTGGATAGATCAAGAAAACAGCAGTAGCAGCTGCAACAGGAGCTGAATATGCAACAGCAATCCAAGGGCGCATACCCAGACGGAAACTAAG[C/T]TCCCACTCACGGCCCATGTAACAAGCTACACCAAGTAAGAAGTGTAGAACAATTAGCTCATAAGGACCGCCATTGTATAACCATTCATCAACAGACGCCGCTTCCCATATTGGGTAAAAATGCAAACCTATAGCTGCAGAAGTAGGAATAATGGCACCAGAAATAATATTGTTTCCATAAAGTAAAGATCCAGAAACAGGTTCACGAATACCATCAATATCTACCGGAGGGGCAGCAATGAAAGCGATAATAAATACAGAAGTTGCGGTCAATAAAGTTGGGATCATCAAAACACCAAACCATCCAATGTAAAGACGGTTTTCAGTGCTGGTTATCCAGTTACAGAAACGACCCCATAAGCTTTCGCTTTCGCGTCTCTCTAAAATTGCAGTCATGGTAAAATCTTGGTTTATTTAATCATCAGGGACTCCCAAGCACACGAATTCTCTATAAAAGAAAAAAAATCGAAATAGATAATTGAGGACTTGTTATTCAACAGT |
| SNP7 | A/T | 1768 | AACAGCAATCCAAGGGCGCATACCCAGACGGAAACTAAGCTCCCACTCACGGCCCATGTAACAAGCTACACCAAGTAAGAAGTGTAGAACAATTAGCTCATAAGGACCGCCATTGTATAACCATTCATCAACAGACGCCGCTTCCCATATTGGGTAAAAATGCAAACCTATAGCTGCAGAAGTAGGAATAATGGCACCAGAAATAATATTGTTTCCATAAAGTAAAGATCCAGAAACAGGTTCACGAATACCATCAATATCTACCGGAGGGGCAGCAATGAAAGCGATAATAAATACAGAAGTTGCGGTCAATAAAGTTGGGATCATCAAAACACCAAACCATCCAATGTAAAGACGGTTTTCAGTGCTGGTTATCCAGTTACAGAAACGACCCCATAAGCTTTCGCTTTCGCGTCTCTCTAAAATTGCAGTCATGGTAAAATCTTGGTTTATTTAATCATCAGGGACTCCCAAGCACACGAATTCTCTATAAAAGAAAA[A/T]AAATCGAAATAGATAATTGAGGACTTGTTATTCAACAGTATAACATGACTTATATACCCGTGTCAACCAATATCAACATTGATGGATATATTTTTATGATCTTATCTATCTAGATTCATCATTCATCCGAACTTTTTTCACTAAATTTGAAGTGAATTAAAAAATAGAATTCAGATTTCTATTTATATATTATAGAATATAGAATTTAAAATAGAATTTAAATTTCTATAGATATGATAAAGCATTTCATCTGATTTCAATATGATAATATATGATAATCATTCAATACGATAATCATAATGGGTTGCCCGGGACTCGAACCCGGAACTAGTCGGATAGAGTAGAAATTTTCATTGTTTGTTAAAAAATAGGTAAAAAAAATCCCTCTCCAAGCCGTGCTTGCATTTTTCATTGCACACGGCTTTCCCTATGTATACATCAAAACCTCAGTTCCCTTCCTAGACGAAACCTCTAAAGAAGTTGAATACTCAGTCGCTCAA |
| SNP8 | C/A | 4432 | TTGAAGCAAGGATAGAGGATTTTTTTTTATTGGGTTATTAAATGCTACATAGTGCGATACAGTAAAAACAAAGTAGTATGATAGAAAAGAATAGATACCTCGGAAATAGGTAAACTCATCAGTGGACTCCCATCCTCTCTTTTTTCCATCTAATTGGTTTATGTTTATTATAAGATAAATAGATAAAAAAAAATGATTAGAAATCCTTTATTTTTTCAAGCCAATCGCTCTTTTGATTTTGGAAAAAAATATCTTTATCAATATACTCTTTCTTCTACACATTAATCTCCCGCTCATACTCATAGTGGGGAATAGCTAATAGTTAGGACTCATTAAAAGAAAATAAAAAATTGGCTAAAGGCCTTTCCCGCATTAGGCACTAATTTTTTTTTAACGTCTAATTAGATTGGATAATCATTCAAATTAAGAACGTAAGCTCGTTGCTTTTTTGTTTCCCTATAATTGGAACCGTAGGACTCTATCCATTTATTCACTCGACA[C/A]AACCTTGAATTCATTTATTATGTTCCGCACCAAGACCTCAAATAACCAAGAATTCAAATAAGGTTTGGCCCGATTCGACAAAAATGAAATATTCTCAGAATTCTCCATTGATACGACATGCTGTTTTTTCCATTCATTCCTTTCAGGATCAGTCGCGGTCTTTCAAACTATACCGATGGTATGGACGAATCCCTTCCTTCATACAAATGCGTAAAAGATATTAGCCGCACTTAAAAGCCGAGTACTCTACCATTGAGTTAGCAACCCCCCCCCCCCAAAAAAAAAAAAATGAAATTCTATAGATACAGCCGGAATCAAAATAAATAAAGAAATTGAAGCACATGACACAATCAAAACATTAAACTAGCATTAAACTAGCAAGAAATTAAATACAAAAAATTTTAATCAATTCTGAATAAAAAAAAATTAAAAATATTCTTTTATTTTATAATTTATAACATTTTTTTCAATCAAACAAAACGTTTGTATTACAACAAATC |
| SNP9 | C/A | 4487 | GATACAGTAAAAACAAAGTAGTATGATAGAAAAGAATAGATACCTCGGAAATAGGTAAACTCATCAGTGGACTCCCATCCTCTCTTTTTTCCATCTAATTGGTTTATGTTTATTATAAGATAAATAGATAAAAAAAAATGATTAGAAATCCTTTATTTTTTCAAGCCAATCGCTCTTTTGATTTTGGAAAAAAATATCTTTATCAATATACTCTTTCTTCTACACATTAATCTCCCGCTCATACTCATAGTGGGGAATAGCTAATAGTTAGGACTCATTAAAAGAAAATAAAAAATTGGCTAAAGGCCTTTCCCGCATTAGGCACTAATTTTTTTTTAACGTCTAATTAGATTGGATAATCATTCAAATTAAGAACGTAAGCTCGTTGCTTTTTTGTTTCCCTATAATTGGAACCGTAGGACTCTATCCATTTATTCACTCGACACAACCTTGAATTCATTTATTATGTTCCGCACCAAGACCTCAAATAACCAAGAATT[C/A]AAATAAGGTTTGGCCCGATTCGACAAAAATGAAATATTCTCAGAATTCTCCATTGATACGACATGCTGTTTTTTCCATTCATTCCTTTCAGGATCAGTCGCGGTCTTTCAAACTATACCGATGGTATGGACGAATCCCTTCCTTCATACAAATGCGTAAAAGATATTAGCCGCACTTAAAAGCCGAGTACTCTACCATTGAGTTAGCAACCCCCCCCCCCCAAAAAAAAAAAAATGAAATTCTATAGATACAGCCGGAATCAAAATAAATAAAGAAATTGAAGCACATGACACAATCAAAACATTAAACTAGCATTAAACTAGCAAGAAATTAAATACAAAAAATTTTAATCAATTCTGAATAAAAAAAAATTAAAAATATTCTTTTATTTTATAATTTATAACATTTTTTTCAATCAAACAAAACGTTTGTATTACAACAAATCTAAAAAAACCATCACTTGAATAAAGAAAAAACCAAATCTATGGAACAGGAATAAA |
| SNP10 | A/C | 4709 | CACATTAATCTCCCGCTCATACTCATAGTGGGGAATAGCTAATAGTTAGGACTCATTAAAAGAAAATAAAAAATTGGCTAAAGGCCTTTCCCGCATTAGGCACTAATTTTTTTTTAACGTCTAATTAGATTGGATAATCATTCAAATTAAGAACGTAAGCTCGTTGCTTTTTTGTTTCCCTATAATTGGAACCGTAGGACTCTATCCATTTATTCACTCGACACAACCTTGAATTCATTTATTATGTTCCGCACCAAGACCTCAAATAACCAAGAATTCAAATAAGGTTTGGCCCGATTCGACAAAAATGAAATATTCTCAGAATTCTCCATTGATACGACATGCTGTTTTTTCCATTCATTCCTTTCAGGATCAGTCGCGGTCTTTCAAACTATACCGATGGTATGGACGAATCCCTTCCTTCATACAAATGCGTAAAAGATATTAGCCGCACTTAAAAGCCGAGTACTCTACCATTGAGTTAGCAACCCCCCCCCCCC[A/C]AAAAAAAAAAAATGAAATTCTATAGATACAGCCGGAATCAAAATAAATAAAGAAATTGAAGCACATGACACAATCAAAACATTAAACTAGCATTAAACTAGCAAGAAATTAAATACAAAAAATTTTAATCAATTCTGAATAAAAAAAAATTAAAAATATTCTTTTATTTTATAATTTATAACATTTTTTTCAATCAAACAAAACGTTTGTATTACAACAAATCTAAAAAAACCATCACTTGAATAAAGAAAAAACCAAATCTATGGAACAGGAATAAATAGATCCACAGAAAAGATCCAATTACCTCAGATCGGATTATATTTATTTGATACACTGTTGTCAATATAAATTGAATAAATTGAGAAAAAAAAAATACACAATGACGAAAACAAAAGAATGAATTGCATTCAATTTAACAATTAAAAAATAAAAAAAAAATAGGTATGAATATAGTATGAATAGAACAAGAGAAGGGGGGGTGTAATAGAGAAAAGGTTATA |
| SNP11 | G/A | 6116 | TCATCAAATCAATTAACTTATGATTTAAGTCCTTTTTCTTCTTTTTTTTTTTTCTTTTTTGGTAAACAAAAAAGAAAAAAAGCATCTGTACCCTTCTAACTCAAGTTGGATAACTTTCAAATAGCTCAAAGGGAACCCCTTAGGCGTTTCATTTTTCACTTATTGAGTGATCTCTAATGCCCTTTCTTTTTCTTTTTGTTTCTTTCTTTTATTTTTAAATGTATAATGTATTTTGATTCTTCATTCTGATCTAATTGTTGAGACAATTGAAAACGGTATTTCCTTGTTCCAGGATCCTTTATCTTTGCCTTGAATCGTTGGGTTTAGACATTACTTCGGTGATCTTTAATCGTTTCGAAATGGCAGCAACATACCACTTTTTGTGATTTCTTTCTATTAAAGAATCAGACTAACAATTGATTCCTGTGTGATACACTTTTTTAATCAAAAGAATTTGGTCAATTCCAACAAACCTTATTCTTGGACTTGAAATTTGCTAG[G/A]ATTGGATCCTTTCTATTTCGATATTGAAAATATACTTACGAAGTTGTTCCAACTTATTGATTAGGGCTAACCCTAGATTCTTGCTCCTAAGAAATAAATCAATACTTTACTTTCTACTCGAGCTCCATCATGTACTATTTACATTACAACCCAATAAAAAACGAAGGTTCTAGTGTATAACAGAACAAACGATGTCGAGCTAAGAGCACCCTCATTCCTATATGCTATTGCTATATGCTATATAATATTATATATAGAGCTATATAATATTAATCTATATACTAATCTATATACTATATAATATATATAATATAATTATATATATATTATATATATAATAGGGGGGATGTAAGAATCCACAGCCGATCGTGTCCTTCAAGTCGCACGTTGCTTTCTACCACATCGTTTCAAACGAAGTTTTACCATAACATTCTTTGAGTTTTGAAATAGTATGTAATTGATTCAATTATGGAATCGTGAATAGTCATTGGTTCGACCGG |
| SNP12 | A/T | 7410 | CTTCTCATTATAAGAGAAGAGAAAAACCCCCTTTTTTAGATACAGATTTGAATTAAACCATCAAGGATTTAAAATAAAATAAATAGGTAAAAAAAATATTCAATTTTTCAGATAGACTATCAGATAGACTAAAGAATTGTCTAAACTAAAGAATTGTCACTGAAATTAATTATGGATATTCTATGTTAAATATTAAAATTAAGCGAGGGATGACAATTTTTTTTTACATTACAAAATCGGAAACAAAGGTGTTAATAAAAAAAAATGCTAAAAATACATACATATAAGCATTTTTTCATTTTATGAATAGTTTATTTGACTTGACTTGATTCTTTCTAAAAATTTTTCCTAAAGTAAAGCGAAAAGTGAATAGGATATATGAATCAATCCCGTCTCAATTGTTAGATAGATTCTCAATTGTTAGATAGATTTAGAATTATTCATTAAAGACAAAGATAAAATATCTGAAAAATGAGGTTAGTTAAAAGAAACTACATATG[A/T]AACATATGTAGTTTCTTTGTTTGTTTTAATTTTCACAGGCACCGGCGAAGGCATTGAAATAGATATCTTCCATTACTAATACTAATTAACTCCTATCTACTTCCCAAATTAAATTATATGGGAGATGTTAAATGATAAATCAAACAAGGAAGGATCCTTTTTTTGATATATAGGAATAGCTGGGACGGAAGGATTCGAACCTCCGAATAGCGGGACCAAAACCCGTTGCCTTACCACTTGGCCACGCCCCATTTGAATTTCTATTCAACACTAATAAAAACTAATATTGGTATTAGCTCTTCGTCAATTCCCACCAAATATCTAGAAAATATATTAGCTTGTTGTTCGGATTTTTCTATGTGTAGATATAGAATTAAATTCAATTTATTGATCATAATATATAATTCAATTAAGATACTGTATAAAAATATGATTTCCTCTATTCTTTTTTGATTTGAGAATTGAAGGATTTTTGATTGGGTGAGTTTAATAAATTTTAT |
| SNP13 | A/T | 7411 | TTCTCATTATAAGAGAAGAGAAAAACCCCCTTTTTTAGATACAGATTTGAATTAAACCATCAAGGATTTAAAATAAAATAAATAGGTAAAAAAAATATTCAATTTTTCAGATAGACTATCAGATAGACTAAAGAATTGTCTAAACTAAAGAATTGTCACTGAAATTAATTATGGATATTCTATGTTAAATATTAAAATTAAGCGAGGGATGACAATTTTTTTTTACATTACAAAATCGGAAACAAAGGTGTTAATAAAAAAAAATGCTAAAAATACATACATATAAGCATTTTTTCATTTTATGAATAGTTTATTTGACTTGACTTGATTCTTTCTAAAAATTTTTCCTAAAGTAAAGCGAAAAGTGAATAGGATATATGAATCAATCCCGTCTCAATTGTTAGATAGATTCTCAATTGTTAGATAGATTTAGAATTATTCATTAAAGACAAAGATAAAATATCTGAAAAATGAGGTTAGTTAAAAGAAACTACATATGA[A/T]ACATATGTAGTTTCTTTGTTTGTTTTAATTTTCACAGGCACCGGCGAAGGCATTGAAATAGATATCTTCCATTACTAATACTAATTAACTCCTATCTACTTCCCAAATTAAATTATATGGGAGATGTTAAATGATAAATCAAACAAGGAAGGATCCTTTTTTTGATATATAGGAATAGCTGGGACGGAAGGATTCGAACCTCCGAATAGCGGGACCAAAACCCGTTGCCTTACCACTTGGCCACGCCCCATTTGAATTTCTATTCAACACTAATAAAAACTAATATTGGTATTAGCTCTTCGTCAATTCCCACCAAATATCTAGAAAATATATTAGCTTGTTGTTCGGATTTTTCTATGTGTAGATATAGAATTAAATTCAATTTATTGATCATAATATATAATTCAATTAAGATACTGTATAAAAATATGATTTCCTCTATTCTTTTTTGATTTGAGAATTGAAGGATTTTTGATTGGGTGAGTTTAATAAATTTTATA |
| SNP14 | A/T | 7412 | TCTCATTATAAGAGAAGAGAAAAACCCCCTTTTTTAGATACAGATTTGAATTAAACCATCAAGGATTTAAAATAAAATAAATAGGTAAAAAAAATATTCAATTTTTCAGATAGACTATCAGATAGACTAAAGAATTGTCTAAACTAAAGAATTGTCACTGAAATTAATTATGGATATTCTATGTTAAATATTAAAATTAAGCGAGGGATGACAATTTTTTTTTACATTACAAAATCGGAAACAAAGGTGTTAATAAAAAAAAATGCTAAAAATACATACATATAAGCATTTTTTCATTTTATGAATAGTTTATTTGACTTGACTTGATTCTTTCTAAAAATTTTTCCTAAAGTAAAGCGAAAAGTGAATAGGATATATGAATCAATCCCGTCTCAATTGTTAGATAGATTCTCAATTGTTAGATAGATTTAGAATTATTCATTAAAGACAAAGATAAAATATCTGAAAAATGAGGTTAGTTAAAAGAAACTACATATGAA[A/T]CATATGTAGTTTCTTTGTTTGTTTTAATTTTCACAGGCACCGGCGAAGGCATTGAAATAGATATCTTCCATTACTAATACTAATTAACTCCTATCTACTTCCCAAATTAAATTATATGGGAGATGTTAAATGATAAATCAAACAAGGAAGGATCCTTTTTTTGATATATAGGAATAGCTGGGACGGAAGGATTCGAACCTCCGAATAGCGGGACCAAAACCCGTTGCCTTACCACTTGGCCACGCCCCATTTGAATTTCTATTCAACACTAATAAAAACTAATATTGGTATTAGCTCTTCGTCAATTCCCACCAAATATCTAGAAAATATATTAGCTTGTTGTTCGGATTTTTCTATGTGTAGATATAGAATTAAATTCAATTTATTGATCATAATATATAATTCAATTAAGATACTGTATAAAAATATGATTTCCTCTATTCTTTTTTGATTTGAGAATTGAAGGATTTTTGATTGGGTGAGTTTAATAAATTTTATAA |
| SNP15 | T/C | 7851 | CATTAAAGACAAAGATAAAATATCTGAAAAATGAGGTTAGTTAAAAGAAACTACATATGAAACATATGTAGTTTCTTTGTTTGTTTTAATTTTCACAGGCACCGGCGAAGGCATTGAAATAGATATCTTCCATTACTAATACTAATTAACTCCTATCTACTTCCCAAATTAAATTATATGGGAGATGTTAAATGATAAATCAAACAAGGAAGGATCCTTTTTTTGATATATAGGAATAGCTGGGACGGAAGGATTCGAACCTCCGAATAGCGGGACCAAAACCCGTTGCCTTACCACTTGGCCACGCCCCATTTGAATTTCTATTCAACACTAATAAAAACTAATATTGGTATTAGCTCTTCGTCAATTCCCACCAAATATCTAGAAAATATATTAGCTTGTTGTTCGGATTTTTCTATGTGTAGATATAGAATTAAATTCAATTTATTGATCATAATATATAATTCAATTAAGATACTGTATAAAAATATGATTTCCTC[T/C]ATTCTTTTTTGATTTGAGAATTGAAGGATTTTTGATTGGGTGAGTTTAATAAATTTTATAAAGTTTAATAAAGAAGGGTTTTTAATCTACCTTTCTTTATCTTTATTTTTTATCAATTTTTATATCTTTTCTTTTTTTTCTTTTATATTAATAACATATTAATAACTCAGTCAAAATAAAATTATCTTCAAGAACAAAATGTTTGTTATGCTTAATATTTTTAGTTTAATTTGTATCTGTCTTAATTCTGCCCTTTATTCAAGCAATTTTTTCTTCACAAAATTGCCCGAAGCCTACGCCTTTTTGAATCCAATCGTAGATGTTATGCCAGTAATCCCTGTACTCTTTTTTCTATTAGCCTTTGTTTGGCAGGCTGCTGTAAGTTTTCGATGAGATCTTTAATGCTGTCCTAAAATAATTCATGATTTATTCGAGAAAAAAAATTATAGCAATTGATAAGATCAGATAAGTCTTATAGTATAAAGTATAAACTCTTAATT |
| SNP16 | T/A | 7908 | TGAAACATATGTAGTTTCTTTGTTTGTTTTAATTTTCACAGGCACCGGCGAAGGCATTGAAATAGATATCTTCCATTACTAATACTAATTAACTCCTATCTACTTCCCAAATTAAATTATATGGGAGATGTTAAATGATAAATCAAACAAGGAAGGATCCTTTTTTTGATATATAGGAATAGCTGGGACGGAAGGATTCGAACCTCCGAATAGCGGGACCAAAACCCGTTGCCTTACCACTTGGCCACGCCCCATTTGAATTTCTATTCAACACTAATAAAAACTAATATTGGTATTAGCTCTTCGTCAATTCCCACCAAATATCTAGAAAATATATTAGCTTGTTGTTCGGATTTTTCTATGTGTAGATATAGAATTAAATTCAATTTATTGATCATAATATATAATTCAATTAAGATACTGTATAAAAATATGATTTCCTCTATTCTTTTTTGATTTGAGAATTGAAGGATTTTTGATTGGGTGAGTTTAATAAATTT[T/A]ATAAAGTTTAATAAAGAAGGGTTTTTAATCTACCTTTCTTTATCTTTATTTTTTATCAATTTTTATATCTTTTCTTTTTTTTCTTTTATATTAATAACATATTAATAACTCAGTCAAAATAAAATTATCTTCAAGAACAAAATGTTTGTTATGCTTAATATTTTTAGTTTAATTTGTATCTGTCTTAATTCTGCCCTTTATTCAAGCAATTTTTTCTTCACAAAATTGCCCGAAGCCTACGCCTTTTTGAATCCAATCGTAGATGTTATGCCAGTAATCCCTGTACTCTTTTTTCTATTAGCCTTTGTTTGGCAGGCTGCTGTAAGTTTTCGATGAGATCTTTAATGCTGTCCTAAAATAATTCATGATTTATTCGAGAAAAAAAATTATAGCAATTGATAAGATCAGATAAGTCTTATAGTATAAAGTATAAACTCTTAATTCAAATATTGAAGTTATTGTATAAATATAAACTATAAACGCGAGAATTCTGGATCACC |
| SNP17 | C/A | 9287 | TAAAAAAATAAAATATATAAAATTAAAATAAAAAAAAAATATAAGGGTTTTTCTTGATTTTTCAATGTTCTTAGTATTTTACTATTCTACATTTTTAAATATTAAATAAAACAAAAAAGTTCGCTAAATTTAAAAGAAAAGAAAAAAATTCCAAGTCATCACCGGAACCGGAAAGAGAGGGATTCGAACCCTCGGTACGGATAACTCGTACAACGGATTAGCAATCCGACGCTTTAGTCCACTCAGCCATCTCTCCCGATTGAAAAAGGATACTTACTATGTTACATTACACAACAGGTAAGGCTTGAAAATAAAAAAAAAGCCTTTTTCCCTCTTTTCTTTATTTTATTACTTTTATTACTTTCAAAATTACTTTTTATATTTTTTTTATTCTTTTATTTTTATATGGAACTATTTTATTTAATTTAATATATTAATTTAATATATTAAATTAAATATATATTCTATAATTATATATTTAATTTGAATAATTATATATT[C/A]AATTTAAATGAAAATTTAAAATAAAATTCAAAATGTCTTACTCGACAAAAGGTTCATTTATATACAATAATCGCATCGTAGCGGGTATAGTTTAGTGGTAAAAGTGTGATTCGTTCTATTAACTCTATTCTAATAGTTAAGGGATCCTTGGCTCCATATTCCGGTGAAAAACTTTATTTCTTAAAAAGATTTAATCCTTTACCTCTCAATGAAAAATTCGAGGAAGAATATACATTCTCGTGATTTGTATCCAATTTTTTGAATTTCTAATTGAAAAAAATTGGATTATGAAATTACGAAACATAATTTTTATTAATTGGATCAATCCATTCAATTGAATGAGTATGAGTAAAGGATCTATGGAAAAAGACAGAAAAGTATATTTCTAATCGTAACTAAATCTTCAATTTTTTCTTTGTTTTGTATAGTCGAGATTGAAGCAAAATAAGTATTAAACGATGACTTTGGTTTACTATAGACATCGACATCTTGTTTTAGCT |
| SNP18 | C/T | 10146 | TATGGAAAAAGACAGAAAAGTATATTTCTAATCGTAACTAAATCTTCAATTTTTTCTTTGTTTTGTATAGTCGAGATTGAAGCAAAATAAGTATTAAACGATGACTTTGGTTTACTATAGACATCGACATCTTGTTTTAGCTCGGTGGAAACAAACTGCCTTTCCTAAGGATTCTTTCAGATAGAAATAGAGAACGAAGTAACTAGAAAGATTAAAAAACCCCCACTCGTCTAGAGGGATCATCTAGAAAGCGCCTTGCTTTGAATGCATTAAGACAGAAAAGCTAACATAGATGTTATGTGTCGAATTTTTTTTTCGCTCACTTCTTATAGCTGGAAATTTTTCCATATTCCATAAAGGAGCCGAATGAAACCAAAGTTTCATGTTCGGTTTTGAATTAGAGACGTTAAAGATGATGAATCAACGTCGACTATAACCCCTAGCCTTCCAAGCTAACGATGCGGGTTCGATTCCCGCTACCCGCTCTATATCTCTATACT[C/T]TCTAAAAAATACTAAAAAATAGAGTCTAAAAATAAAAAAAAAATAGAGATAATATTTTTATTCTAATATATTTATATAGAATATAATAGAATTTTATATAGAATATAATAGAATATAGAATATTTCTAAAAAAATATTAATTCAAAAAATTTAAATTATTTATTTAAAATTTTTGAATAGTTTTTTAATAGTCTAATTAAATTGGACTCCATATACGTATTTTCTATTTTTCTATTATTAATTTTCTATTTTTCTATTATTATAATTATATTATAATTATTAATATTATTATTCTAATTATTAATATTATTATTATATTATAATATTATATAATATTATATAAATTTTTTAATATTAATATTATATAAAATATTATATAAATTTTTTAATTTTATATTTTATATTATATAAATAGCTAACTATATATTAACAATATATAATAACCCTCTATTCTATATAGTTAGTTATTATAGAAGTTATTAAGTTTTTATATATTTCTA |
| SNP19 | T/G | 10641 | ATACTCTCTAAAAAATACTAAAAAATAGAGTCTAAAAATAAAAAAAAAATAGAGATAATATTTTTATTCTAATATATTTATATAGAATATAATAGAATTTTATATAGAATATAATAGAATATAGAATATTTCTAAAAAAATATTAATTCAAAAAATTTAAATTATTTATTTAAAATTTTTGAATAGTTTTTTAATAGTCTAATTAAATTGGACTCCATATACGTATTTTCTATTTTTCTATTATTAATTTTCTATTTTTCTATTATTATAATTATATTATAATTATTAATATTATTATTCTAATTATTAATATTATTATTATATTATAATATTATATAATATTATATAAATTTTTTAATATTAATATTATATAAAATATTATATAAATTTTTTAATTTTATATTTTATATTATATAAATAGCTAACTATATATTAACAATATATAATAACCCTCTATTCTATATAGTTAGTTATTATAGAAGTTATTAAGTTTTTATATA[T/G]TTCTATTATAACTAGTTAATATCTATTATAACTATATAATATAAAGTTATAGTATATAGTTATAATTTGTTCTATATAAATAGACTCTAGATAGTGTATATACTATCTTTATATTAATATACTATCTTTATATTATTAGAATATAAATTAATAAGAAACTAGAATAATGTTAGAAAAAGTAATTGTAAATTGTAATAAAAAAAATTGAAAATTCTAATAATCTAATAATAAATTCTAAAAATTAGAATATTTCATTCTAAAATTAAATAATTCTAAAATGAATATAAAAGAATTAATCCATCATTTACATTTACATTTTTTACATTTCATTTTTAATTCAAAAAATAGGTAATTCCTAAAATTATCTTGCATATTCTTTTTTTTATGAACAAAAAATATGAAAGAAAAAAGAAAAAAATTGGAATGGAATAGAAAAGCGTCCATTGTCTAATGGATAGGACAGAGGTCTTCTAAACCTTTGGTATAGGTTCAAATCCTAT |
| SNP20 | T/G | 10847 | ATTGGACTCCATATACGTATTTTCTATTTTTCTATTATTAATTTTCTATTTTTCTATTATTATAATTATATTATAATTATTAATATTATTATTCTAATTATTAATATTATTATTATATTATAATATTATATAATATTATATAAATTTTTTAATATTAATATTATATAAAATATTATATAAATTTTTTAATTTTATATTTTATATTATATAAATAGCTAACTATATATTAACAATATATAATAACCCTCTATTCTATATAGTTAGTTATTATAGAAGTTATTAAGTTTTTATATATTTCTATTATAACTAGTTAATATCTATTATAACTATATAATATAAAGTTATAGTATATAGTTATAATTTGTTCTATATAAATAGACTCTAGATAGTGTATATACTATCTTTATATTAATATACTATCTTTATATTATTAGAATATAAATTAATAAGAAACTAGAATAATGTTAGAAAAAGTAATTGTAAATTGTAATAAAAAAAAT[T/G]GAAAATTCTAATAATCTAATAATAAATTCTAAAAATTAGAATATTTCATTCTAAAATTAAATAATTCTAAAATGAATATAAAAGAATTAATCCATCATTTACATTTACATTTTTTACATTTCATTTTTAATTCAAAAAATAGGTAATTCCTAAAATTATCTTGCATATTCTTTTTTTTATGAACAAAAAATATGAAAGAAAAAAGAAAAAAATTGGAATGGAATAGAAAAGCGTCCATTGTCTAATGGATAGGACAGAGGTCTTCTAAACCTTTGGTATAGGTTCAAATCCTATTGGACGCAATTTTTTTTCATATAATATACATATTATAATATAATAATCTATATATAGATTATATAGATTTTATATAGATTCTAAAATATAAATATATAGATTATAAATTTATAAATTATAAAATAAAATTTTTATAATTAAAAGTTAAAGATTAAAAGTGATTAATTTCTTTATACTTATACCTGTAGTGATTTATTTCTTTAGAC |
| SNP21 | G/T | 22460 | TTGTTGTTCAGCATCTTGAACTAGCCATCCCTTAGAAGGTATTGTTAAAAGATCATCAATTCCTAATGAAATGGATGTAGCAGTGGCTTGCTGGAAACCCAGAGTCTTTACTTGATCCAGGATGTGTGATGTATATGCCATTCCAAAATGATCTATTAATCTGCTAATAAGTCGTTTAATGGCAGTTCCATCTATCACTTTATTGTGAAAGACCAGATTGGCCCGTTTGGCCATAAGTACCTCCATATTCCGCTGAGTAGAGTTCGACAATGGATTTGAGTCAATGATTGGAAACTTCCTTTTCTCGATCTTGATTTGCATAGAAATGAAATTCAGGAACTAGGGTCCTAGCTGAACCGAAGAGATCGGAATTCATGCCAGTGTCATAGAATTACTTAGCTTAGATCCCTATGATTAGATACCATCTGAGCCGGCTTGGCAAAACCCTTGTATAGCTTCTTCGATTTCTCGATAAAGAGAAATATGACCAACAGTAGTTC[G/T]AATGTATATACAAAGAATTTCTTTTTTTATATTTCTTACTATTAGATAGTGTTCATAAATCTCATGATAGGTACCCAAAGATTCATAGTGAACTTCGACGGGAGCTTCTCTTGAAGCAATAGCGCGTTGATCTAGTTGCCAACGGAGCCACAAAGGACTATCTAAATTGATTCTTTTCTGCCGATAAGCACCAATTGCATCATAGGAATTAGAAAAAAAGGATTCTTTCGTATATTGATTGTTATTATCGTAAATTCTTTCATTTTGATAATTTCTGTGATTACATGGATTATACCTATTTGCACAAATACCTCGGCGATTCCTGCTTGTTAATACATAGAGCCCAATAAGCATATCTTGAGTTGGTACGGAAATGGGATCTCCAATAGCTGGAGATAAGAGATTCATATGAGAAAACATAAGTAAACGAGCCTCCGCTTGAGCCTCCAACGATAAAGGTACATGAACAGCCATTTGATCCCCATCAAAGTCTGCATTGA |
| SNP22 | A/C | 22602 | CCAAAATGATCTATTAATCTGCTAATAAGTCGTTTAATGGCAGTTCCATCTATCACTTTATTGTGAAAGACCAGATTGGCCCGTTTGGCCATAAGTACCTCCATATTCCGCTGAGTAGAGTTCGACAATGGATTTGAGTCAATGATTGGAAACTTCCTTTTCTCGATCTTGATTTGCATAGAAATGAAATTCAGGAACTAGGGTCCTAGCTGAACCGAAGAGATCGGAATTCATGCCAGTGTCATAGAATTACTTAGCTTAGATCCCTATGATTAGATACCATCTGAGCCGGCTTGGCAAAACCCTTGTATAGCTTCTTCGATTTCTCGATAAAGAGAAATATGACCAACAGTAGTTCGAATGTATATACAAAGAATTTCTTTTTTTATATTTCTTACTATTAGATAGTGTTCATAAATCTCATGATAGGTACCCAAAGATTCATAGTGAACTTCGACGGGAGCTTCTCTTGAAGCAATAGCGCGTTGATCTAGTTGCCA[A/C]CGGAGCCACAAAGGACTATCTAAATTGATTCTTTTCTGCCGATAAGCACCAATTGCATCATAGGAATTAGAAAAAAAGGATTCTTTCGTATATTGATTGTTATTATCGTAAATTCTTTCATTTTGATAATTTCTGTGATTACATGGATTATACCTATTTGCACAAATACCTCGGCGATTCCTGCTTGTTAATACATAGAGCCCAATAAGCATATCTTGAGTTGGTACGGAAATGGGATCTCCAATAGCTGGAGATAAGAGATTCATATGAGAAAACATAAGTAAACGAGCCTCCGCTTGAGCCTCCAACGATAAAGGTACATGAACAGCCATTTGATCCCCATCAAAGTCTGCATTGAATCCCTTACAAACTAATGGATGTAAACAAATAGCGCGGCCTTCCACTAAAATGGGTTGGAATGCCTGTATGCCTAATCTATGCAAAGTCGGCGCTCTATTCAGTAATACCGGATGCCCCTGCATAACTTCCTGAAGTATTTC |
| SNP23 | A/G | 25850 | ATGATTGTAGTACCAAGCACTTCTTGGCGAGCTCTAATATGATCAGATTTATAAGTAAGCATTTCTTGTAAAATATGAGAAACACCAAATCCTTCTAGAGCCCAAACCTCCATTTCTCCGACTCGTTGTCCCCCTTGCTTGGCCCTTCCTCTAAGGGGTTGTTGGGTAACAAGTGCATAATGTCCACTGGAACGTCCATGTATTTTATCATCAACTTGATGAATTAATTTCAAGATATAAGGCTTTCCTATTATAACAGGCTGCTCAAAGGGATCCCCCGTTCTTCCATCAAATATTCTACTTTTTCCCGGATATTCGGGTTCAAATACCCACGGATTTGCTGTTTGCTTACTGGCTTCATATAATTCAGAAAATACAAGTTTTCTCGAGGCCTCTTGTTCATATCTCTCATCAAAGGGTGCTATTCGATAATGTCTATCTAGCAGACCTCCCGCTAACCCGAGTGAGCATTCAAATATCTGTCCTACATTCATTCGTGA[A/G]GGTACTCCTAATGGGTTGAAGACCATATCAACAGGCCTTCCATCTTGCAAATAAGGCATATCTTGTCTAGGCAAAATTTTGGAAATGATGCCTTTATTTCCATGTCTTCCAGCGACTTTATCACCCACTTTGATTTCACGTTTCTGTAAAATATATATACGAATCGTTTCCGGATTATAACTGGAACCCCCCTTTTTCTGGACCCATCTCACATCAATAACTCGCCCCCTACCACCTATAGGTAGTTTTAGACAAGTTTCCTTTGAAGTAGATACCTGAATACCAAGTATGGCTCTTAATAATCTATCTTCGGGAGCATACGATGATTCTTTCGCCATTTGAGGCGTTAATTTACCTACTAAAATATCGCCCGTCTCTACCCAAGATCCTAGCATCACAATTCCATTTTTGTCTAAATTTCGGAGTAAATGGGCTTCTAGATGCGGTATTTCATTAGTGATCCTTTCAGGTCCTTGGCTTGTCACATGAGTCTGAATTTC |
| SNP24 | C/T | 26541 | CCTTTTTCTGGACCCATCTCACATCAATAACTCGCCCCCTACCACCTATAGGTAGTTTTAGACAAGTTTCCTTTGAAGTAGATACCTGAATACCAAGTATGGCTCTTAATAATCTATCTTCGGGAGCATACGATGATTCTTTCGCCATTTGAGGCGTTAATTTACCTACTAAAATATCGCCCGTCTCTACCCAAGATCCTAGCATCACAATTCCATTTTTGTCTAAATTTCGGAGTAAATGGGCTTCTAGATGCGGTATTTCATTAGTGATCCTTTCAGGTCCTTGGCTTGTCACATGAGTCTGAATTTCATATTTCCGTATGTGAAAAGAAGTATAAATATCTTCATATACCAGACGTTCGCTAATGAGTACCGCATCCTCAAAATTGTAACCTTCCCACGGCATATAAGCCACTAATACGTTTTTGCCCAAGGCGAGTTCGCCACCAATTGTAGCGGCACCATCCGCTAAAACTTGTCCCTTTTTAATGCATTTACCC[C/T]GCGGAACCTGGGGTTTTTGATGCATACAAGTATTTTTGTTGGAACGTTGATACATAACTAATGGAATGCGTAGAGTATCCCCATTGCCCGATAAAATGATCTTGTCAATATCAGTATAAATTATCTTTCCTTCGCGTTCGGCTATAGCGGGAACCCCTGAATCTAGAGCCACTTGGCGTTCCAATCCAGTCCCAACAATGCATTTTTCGGACCGAGAAAGTGGAACTGCTTGACGTTGCATATTAGAACTCATTAAAGCCCGATTCGCATCATTATGCTCGATAAAAGGAATGAGGGAAGCTCCGATAGAAAAATATTGGAAGGGAAAAATACTTCGAAGATGCACTTGTTCCCATGCAATAGTCAGGAATTCTTGACGATATCGAGCTGGGGCAACCTGTTCTTCCTGAACACCTCGATTTAGCGCCAAAGAATTTCCTGCCGCTACCATATGGTATTCCTCTCTATTTGGTGATAAATAAAACATCCGTACTTTTTTT |
| SNP25 | C/A | 28799 | TCAATTTTTGGAAACTTATAAAGTTCTTCTGTTAAGCCCTGATCAATGAACCTACAAAATCCTTCAAATTGTATCTGATTCAATCCAGGTATTGTAGACATTCCCTCATTTCCATCCCCGAGCATTTTGAATTTCCCGTTTATTAAAAAAATTCCATTATTGTATTGGATCGTTCTTCATTCAAATTATATGGATCGATCTAGCAATGATGGAATTTATATTCTGTTTACAGAATCACATAAAATTTTATCTATCTCCATAGATATGGAATGTATGAAATACGTATGAACGGAGGAATAAAGAGAAGTTTATACTCAAATTCGAATTTGCAACAGAGACAGCTGGAAAAGAATTGAGAAATTCCACTTACCTTAGACTTATGAAATTTTGTATAGAATAGCAAAACAAAAAGTGATTCAATTTCTACCATTATTATGATATTACATATTCCAATACGCTTGGATACCAATTTCCAATACGCTTGGATACCAATAAAATAA[C/A]TGAATTCGGGATTAAATCTTTCGATGAGAGAAAAAACCAATAATCCGAAACAATATAAAGTTGATTTTTTGATTTTTTTTTTAGCACTTAGCTTTTTCGCGGATTTCATTGTTTAGTTCAAAAAAATTTGCATAGAAGAGAGATTTTTTTATATCTTTTTTTTAATCGAGTTCATATAATACGATTGAAGTGCATAACATAAAACAAACATAAGAAGGCTTATTAAACATATGCGGGCATAACTATAGCTATCTCATAATTATAGCTATCTATACTATATATACGTCTCTCCTTTTACTGCAGTTCTATTTTGGACAGCGCATGTCATGCTCTATCAAAATTTATATTCCATTTTCATTGAATAGAAATTTTCCTATAGGAATCATAGACGGATAAGATAGATATCCGCATAAAATTTTATGTTCTAGGGTTTACATATACTCATAATTGTTGTTATAATTGAAACTGAGAAGAATTTTTTTTTATTGAAAAGAATCAAT |
| SNP26 | G/T | 28862 | TCAAATTGTATCTGATTCAATCCAGGTATTGTAGACATTCCCTCATTTCCATCCCCGAGCATTTTGAATTTCCCGTTTATTAAAAAAATTCCATTATTGTATTGGATCGTTCTTCATTCAAATTATATGGATCGATCTAGCAATGATGGAATTTATATTCTGTTTACAGAATCACATAAAATTTTATCTATCTCCATAGATATGGAATGTATGAAATACGTATGAACGGAGGAATAAAGAGAAGTTTATACTCAAATTCGAATTTGCAACAGAGACAGCTGGAAAAGAATTGAGAAATTCCACTTACCTTAGACTTATGAAATTTTGTATAGAATAGCAAAACAAAAAGTGATTCAATTTCTACCATTATTATGATATTACATATTCCAATACGCTTGGATACCAATTTCCAATACGCTTGGATACCAATAAAATAACTGAATTCGGGATTAAATCTTTCGATGAGAGAAAAAACCAATAATCCGAAACAATATAAAGTT[G/T]ATTTTTTGATTTTTTTTTTAGCACTTAGCTTTTTCGCGGATTTCATTGTTTAGTTCAAAAAAATTTGCATAGAAGAGAGATTTTTTTATATCTTTTTTTTAATCGAGTTCATATAATACGATTGAAGTGCATAACATAAAACAAACATAAGAAGGCTTATTAAACATATGCGGGCATAACTATAGCTATCTCATAATTATAGCTATCTATACTATATATACGTCTCTCCTTTTACTGCAGTTCTATTTTGGACAGCGCATGTCATGCTCTATCAAAATTTATATTCCATTTTCATTGAATAGAAATTTTCCTATAGGAATCATAGACGGATAAGATAGATATCCGCATAAAATTTTATGTTCTAGGGTTTACATATACTCATAATTGTTGTTATAATTGAAACTGAGAAGAATTTTTTTTTATTGAAAAGAATCAATACGGATTGGTTACGTATCAATTTTTATTTTCTTATATCATTAGGATTAGAGAAATACAATTTT |
| SNP27 | G/T | 28870 | TATCTGATTCAATCCAGGTATTGTAGACATTCCCTCATTTCCATCCCCGAGCATTTTGAATTTCCCGTTTATTAAAAAAATTCCATTATTGTATTGGATCGTTCTTCATTCAAATTATATGGATCGATCTAGCAATGATGGAATTTATATTCTGTTTACAGAATCACATAAAATTTTATCTATCTCCATAGATATGGAATGTATGAAATACGTATGAACGGAGGAATAAAGAGAAGTTTATACTCAAATTCGAATTTGCAACAGAGACAGCTGGAAAAGAATTGAGAAATTCCACTTACCTTAGACTTATGAAATTTTGTATAGAATAGCAAAACAAAAAGTGATTCAATTTCTACCATTATTATGATATTACATATTCCAATACGCTTGGATACCAATTTCCAATACGCTTGGATACCAATAAAATAACTGAATTCGGGATTAAATCTTTCGATGAGAGAAAAAACCAATAATCCGAAACAATATAAAGTTGATTTTTT[G/T]ATTTTTTTTTTAGCACTTAGCTTTTTCGCGGATTTCATTGTTTAGTTCAAAAAAATTTGCATAGAAGAGAGATTTTTTTATATCTTTTTTTTAATCGAGTTCATATAATACGATTGAAGTGCATAACATAAAACAAACATAAGAAGGCTTATTAAACATATGCGGGCATAACTATAGCTATCTCATAATTATAGCTATCTATACTATATATACGTCTCTCCTTTTACTGCAGTTCTATTTTGGACAGCGCATGTCATGCTCTATCAAAATTTATATTCCATTTTCATTGAATAGAAATTTTCCTATAGGAATCATAGACGGATAAGATAGATATCCGCATAAAATTTTATGTTCTAGGGTTTACATATACTCATAATTGTTGTTATAATTGAAACTGAGAAGAATTTTTTTTTATTGAAAAGAATCAATACGGATTGGTTACGTATCAATTTTTATTTTCTTATATCATTAGGATTAGAGAAATACAATTTTCGATTGAA |
| SNP28 | T/G | 29324 | TGAGAGAAAAAACCAATAATCCGAAACAATATAAAGTTGATTTTTTGATTTTTTTTTTAGCACTTAGCTTTTTCGCGGATTTCATTGTTTAGTTCAAAAAAATTTGCATAGAAGAGAGATTTTTTTATATCTTTTTTTTAATCGAGTTCATATAATACGATTGAAGTGCATAACATAAAACAAACATAAGAAGGCTTATTAAACATATGCGGGCATAACTATAGCTATCTCATAATTATAGCTATCTATACTATATATACGTCTCTCCTTTTACTGCAGTTCTATTTTGGACAGCGCATGTCATGCTCTATCAAAATTTATATTCCATTTTCATTGAATAGAAATTTTCCTATAGGAATCATAGACGGATAAGATAGATATCCGCATAAAATTTTATGTTCTAGGGTTTACATATACTCATAATTGTTGTTATAATTGAAACTGAGAAGAATTTTTTTTTATTGAAAAGAATCAATACGGATTGGTTACGTATCAATTTT[T/G]ATTTTCTTATATCATTAGGATTAGAGAAATACAATTTTCGATTGAAATCCAAGAATCGTTTATGAATTCACAGTTAATAGTTAATGGTTCCAATTTGTCCTTAATTTTTGAGTTTGTGACTGAAAATTGACTTTTCATTTCAATAGATAATAGAAAAATTTAGATAGTGTGTGTTTTGAGATACTATACAAAATAAATGGAAAAGATAGATCCAATCCAAAAAAGAAAAAAAGAAACTCCTCCCTCTAAAAATAAAAAAAAAGCAAAAAAAAAGGGTGGGAATATTTTCGTCTATTTTGTGTTGCCTTGGCGGCATGGCCGAGTGGTAAGGCGGGGGACTGCAAATCCTTTTTCCCCAGTTCAAATCCGGGTGTCGCCTGATCAACAAAAAACGCAAAATATCTTATTCCCTTTTTTTGCTGATATAACTTATCAAATTTGCTCCCAACAGAAGTACGCGGAGGAAAAGGCTTCTTGATACTGATACTTCCCAGGGTATT |
| SNP29 | A/G | 32205 | TCTTGATTCAATTGATAATTTTTTTTTCCATTACTTTCTTTTTTTTTTATTTGATATTTATTTTATTAATATCCCCATTCTTTGGTAATGGAATGCATAGAATATATTAAAAATAAAAAAACTTCAGTGCGCAATTTGAATGAGATAGATTGTTTCAAATTCAAATTTGTAATTGAGATTACACAAAATCGGAGCCAAAAAAAAAGAGACTTTTCAGATAAAGATTAAAAGGATTCTATCAAAGCAATTAAATTGATTTTGTATCGTACATACATACATACAAATAAATAAAAATTCCATTTGTGTATGCGTTACCGGAAAAGATATGGTACTTGATTCGATTTCATTATATGGGTCGGGAGTAATCAAAAAATCCAAACTCAGTATAGTATCTTTTTGAATCTTGGATATAACGTTACCAATAATTGTACTAATCCACATATGTCCCTATCAATCAGTACTGGTTGAAAAAATGAAAATTCCCGTATTTGTATTTACTT[A/G]GATGAGAAATGTAAAACGAAAAGAAAATAAATGGTCCCCTTGGGAATGAAATTATGCTATTTGTCCCCCTTTCGCAGAAAAGGGAAAGATGAATTAATGTATTTTTTATTAGATTATTGGATTTGGATCCGTCGGGACTGACGGGGCTCGAACCCGCAGCTTCCGCCTTGACAGGGCGGTGCTCTGACCACTTGAACTACAATCCCCGGGAAATGCAATACAAGAAAGTGTACAGCATATATATTCTTATGATTTCATTCATACCCCTTCTATTTACTATTTAAATTTTACTATTTCGGTTTTAGACTGGAATCACCGCGTTGTATATAACAGAAACGGGGGTAGTATATTGATATCCACATGGATATGCACTTTAGTGCTAAAAGGGATAGGGAGTACTAGTCAGCTTTTCGTTATTTCAAAATCTGAATCTAGATCATTAGCAAGATCAGAATTTATGAGAAAAAAACTAAATAAAATAAAGCAAATCAAATAAAGAA |
| SNP30 | A/C | 32488 | ATAAATAAAAATTCCATTTGTGTATGCGTTACCGGAAAAGATATGGTACTTGATTCGATTTCATTATATGGGTCGGGAGTAATCAAAAAATCCAAACTCAGTATAGTATCTTTTTGAATCTTGGATATAACGTTACCAATAATTGTACTAATCCACATATGTCCCTATCAATCAGTACTGGTTGAAAAAATGAAAATTCCCGTATTTGTATTTACTTAGATGAGAAATGTAAAACGAAAAGAAAATAAATGGTCCCCTTGGGAATGAAATTATGCTATTTGTCCCCCTTTCGCAGAAAAGGGAAAGATGAATTAATGTATTTTTTATTAGATTATTGGATTTGGATCCGTCGGGACTGACGGGGCTCGAACCCGCAGCTTCCGCCTTGACAGGGCGGTGCTCTGACCACTTGAACTACAATCCCCGGGAAATGCAATACAAGAAAGTGTACAGCATATATATTCTTATGATTTCATTCATACCCCTTCTATTTACTATTT[A/C]AATTTTACTATTTCGGTTTTAGACTGGAATCACCGCGTTGTATATAACAGAAACGGGGGTAGTATATTGATATCCACATGGATATGCACTTTAGTGCTAAAAGGGATAGGGAGTACTAGTCAGCTTTTCGTTATTTCAAAATCTGAATCTAGATCATTAGCAAGATCAGAATTTATGAGAAAAAAACTAAATAAAATAAAGCAAATCAAATAAAGAACAGGTAAAGATATATCCGAAATTATGACTTTTTTTTTTTCTGTATCAGGCATTTCGAGAGAACAAAGGGGTTATATCATTCATGGCGGATCAGTGAATTATTGGGCCGAGCTGGATTTGAACCAGCGTAGACATATTGCCAACGAATTTACAGTCCGTCCCCATTAACCGCTCGGGCATCGACCCAGGAAGAATCAATTCCAGACTTATTAAGAATCCATGATCAACTTCCTTTCGTAATACCCTACCCCCAGGGGAAGTCGAATCCCCGCTGCCTCCTTGAA |
| SNP31 | T/C | 32746 | TGGGAATGAAATTATGCTATTTGTCCCCCTTTCGCAGAAAAGGGAAAGATGAATTAATGTATTTTTTATTAGATTATTGGATTTGGATCCGTCGGGACTGACGGGGCTCGAACCCGCAGCTTCCGCCTTGACAGGGCGGTGCTCTGACCACTTGAACTACAATCCCCGGGAAATGCAATACAAGAAAGTGTACAGCATATATATTCTTATGATTTCATTCATACCCCTTCTATTTACTATTTAAATTTTACTATTTCGGTTTTAGACTGGAATCACCGCGTTGTATATAACAGAAACGGGGGTAGTATATTGATATCCACATGGATATGCACTTTAGTGCTAAAAGGGATAGGGAGTACTAGTCAGCTTTTCGTTATTTCAAAATCTGAATCTAGATCATTAGCAAGATCAGAATTTATGAGAAAAAAACTAAATAAAATAAAGCAAATCAAATAAAGAACAGGTAAAGATATATCCGAAATTATGACTTTTTTTTTTTC[T/C]GTATCAGGCATTTCGAGAGAACAAAGGGGTTATATCATTCATGGCGGATCAGTGAATTATTGGGCCGAGCTGGATTTGAACCAGCGTAGACATATTGCCAACGAATTTACAGTCCGTCCCCATTAACCGCTCGGGCATCGACCCAGGAAGAATCAATTCCAGACTTATTAAGAATCCATGATCAACTTCCTTTCGTAATACCCTACCCCCAGGGGAAGTCGAATCCCCGCTGCCTCCTTGAAAGAGAGATGTCCTGAACCACTAGACGATGGGGGCGCATTTGCCCGACCACCAGCATACTATGCTCATAGTATGAACAGTTTTTTGAAATTGTCAATATAACGAAATGGTATGACTAGATTCGAAGAATCTTTCCGCCCTTCATGATTCGATAGAATTTTGTGATTCTTATATTCGCCATTATCCATTCTAAGAATAATCAATAATTCTAAGAATATTAAATAGAATTAATTTAATAAATAAATATTAAATATAAATAA |
| SNP32 | A/T | 33675 | TCTAAGAATAATCAATAATTCTAAGAATATTAAATAGAATTAATTTAATAAATAAATATTAAATATAAATAAAATAAATAGAATTAAATTAATAAGAATTAATAATAAGAATTAATAAATTAATATAAAATAAATAGAAAATAAAATAGAAAAAAATTAATAATTAATATATAAATATAATAAATATAAATAGAATGAATTTAGAAAGAATGAATTTAGAATTCATAATTCTATTAATATTAGTAATATAATAGAATTAATTATTAATATTATATATTAATATTATATATATATTTTTGAATTTAGTACAAATTGAATATTTAATAAAAAAACAAAATAGAAAAAAAAAAAAAATAGAAATAATACGAAAGATTCTTTCAACGGGTCTGCCAGAAAACCAGAAAATAGGGTTAAACCTTACTTCTTTCGCTTTCGTTCATTAATTCACTCATTGTTAAGATAAATAGGCATATCTCTCTCTCACACTAAACCAGTAATTT[A/T]ACAAATGAGAAATCTGGGAAGAGGGATAGGCATCAACAAGTTATCCAATTTGATTTTAAGAATTTGGTTGAGGGGACAAATAGAATCTTTTCATCAGCGATTCGATGAAATATCTTGGATCTATGTTGAATTGCTAAATACATGTATCAATCAAGCGAATTTCGGTGGTGAGGTTCGGCCTTGATAAATTCATTACATTGATATTTATATTTATCAAATTCAATATCAATAAACTCTTTTTTTACCTATATTCACTAGTTTAGTCTAAACTCACTAGGTAAATCAAACCTTTCGCTTATAAATGAACTACTATGAATCTACTTCCTATACTTAGTATGTATAATATACTTATTCTAATTTATATTAGTTTCTATTAGAATTTATGAATTTAGAATAGAATTCTAAATTAGAATAATAATATTCCATGTTTAGAATAATCTAATTTTTCTAATATCTAATAATAGTATTAAAAAATAATGTTATTATTTTATATTTTAGAT |
| SNP33 | A/C | 34929 | TATATTATATTTATAATTTATTTATATTATATTATATTTATTTATATTATATTTTATTTTTATTATAATATATAATAATTATAATATTTATTTAATCTTTATTATAATTTAATATTAATAATTTATTATATATAAATATATAATATTTATTAGAATATTATATATATTCTAATTTTATATATTAGATTAGAATTATATTCTAATTTCATTCTATATATTAGATTCTAAATTTATATATAATTATATATATATTAGATTCTAATTTATATATATATATTAGATATTAGAATTATATTCTAATTTAATAAATTGAATATTAAATAAATATAATATTAATATTATAATATAATAATATAAATATATATTAATATTAAAATATTAATATATTAATATAAATATATTCAAATATATTAAAATATTAAAATCAAATATTAATATTATTATATTAAAAATCAAATATTAATATTATTATATTAAAAATCAAATTATTATATTAAAAT[A/C]TATTAAAATATTTAAATAATATTAAAATATTAAAATAGATTTATAGATTTACTTTATTTATTTACTTATTTTTTTTTCTATAGATCTATCTTATCCACATAGTAGCTCATTCAGGAATTGAATCAAACGGGCCCTTTTAACTCAGTGGTAGAGTAACGCCATGGTAAGGCGTAAGTCATCGGTTCAAATCCGATAAGGGGCTTTGGCCTTTTTTTCTTTTTAATTTTAATAAAAAGAAATAAAAAGAAAAAACCTGCGATAATATTCCTATTTGATAATATTTCTATTTGAAGAGGGAATAGAAATATTGTTGATATTTGTCATAAAAAAAGTAAGAAACTCTAGAATTTAAATTAATTCTAAAATGGAAATTAGAAATTTTTGAAAATTAACATTATACATTATAATAAGTTATACCATATCAATACTCTATTAAAACTCTATTAAACGAATATTATAATGATTTTTTATAATGATTTTTAGTATATAAATTTTTACTA |
| SNP34 | A/T | 34934 | TATATTTATAATTTATTTATATTATATTATATTTATTTATATTATATTTTATTTTTATTATAATATATAATAATTATAATATTTATTTAATCTTTATTATAATTTAATATTAATAATTTATTATATATAAATATATAATATTTATTAGAATATTATATATATTCTAATTTTATATATTAGATTAGAATTATATTCTAATTTCATTCTATATATTAGATTCTAAATTTATATATAATTATATATATATTAGATTCTAATTTATATATATATATTAGATATTAGAATTATATTCTAATTTAATAAATTGAATATTAAATAAATATAATATTAATATTATAATATAATAATATAAATATATATTAATATTAAAATATTAATATATTAATATAAATATATTCAAATATATTAAAATATTAAAATCAAATATTAATATTATTATATTAAAAATCAAATATTAATATTATTATATTAAAAATCAAATTATTATATTAAAATATATT[A/T]AAATATTTAAATAATATTAAAATATTAAAATAGATTTATAGATTTACTTTATTTATTTACTTATTTTTTTTTCTATAGATCTATCTTATCCACATAGTAGCTCATTCAGGAATTGAATCAAACGGGCCCTTTTAACTCAGTGGTAGAGTAACGCCATGGTAAGGCGTAAGTCATCGGTTCAAATCCGATAAGGGGCTTTGGCCTTTTTTTCTTTTTAATTTTAATAAAAAGAAATAAAAAGAAAAAACCTGCGATAATATTCCTATTTGATAATATTTCTATTTGAAGAGGGAATAGAAATATTGTTGATATTTGTCATAAAAAAAGTAAGAAACTCTAGAATTTAAATTAATTCTAAAATGGAAATTAGAAATTTTTGAAAATTAACATTATACATTATAATAAGTTATACCATATCAATACTCTATTAAAACTCTATTAAACGAATATTATAATGATTTTTTATAATGATTTTTAGTATATAAATTTTTACTATATAA |
| SNP35 | A/T | 34935 | ATATTTATAATTTATTTATATTATATTATATTTATTTATATTATATTTTATTTTTATTATAATATATAATAATTATAATATTTATTTAATCTTTATTATAATTTAATATTAATAATTTATTATATATAAATATATAATATTTATTAGAATATTATATATATTCTAATTTTATATATTAGATTAGAATTATATTCTAATTTCATTCTATATATTAGATTCTAAATTTATATATAATTATATATATATTAGATTCTAATTTATATATATATATTAGATATTAGAATTATATTCTAATTTAATAAATTGAATATTAAATAAATATAATATTAATATTATAATATAATAATATAAATATATATTAATATTAAAATATTAATATATTAATATAAATATATTCAAATATATTAAAATATTAAAATCAAATATTAATATTATTATATTAAAAATCAAATATTAATATTATTATATTAAAAATCAAATTATTATATTAAAATATATTA[A/T]AATATTTAAATAATATTAAAATATTAAAATAGATTTATAGATTTACTTTATTTATTTACTTATTTTTTTTTCTATAGATCTATCTTATCCACATAGTAGCTCATTCAGGAATTGAATCAAACGGGCCCTTTTAACTCAGTGGTAGAGTAACGCCATGGTAAGGCGTAAGTCATCGGTTCAAATCCGATAAGGGGCTTTGGCCTTTTTTTCTTTTTAATTTTAATAAAAAGAAATAAAAAGAAAAAACCTGCGATAATATTCCTATTTGATAATATTTCTATTTGAAGAGGGAATAGAAATATTGTTGATATTTGTCATAAAAAAAGTAAGAAACTCTAGAATTTAAATTAATTCTAAAATGGAAATTAGAAATTTTTGAAAATTAACATTATACATTATAATAAGTTATACCATATCAATACTCTATTAAAACTCTATTAAACGAATATTATAATGATTTTTTATAATGATTTTTAGTATATAAATTTTTACTATATAAA |
| SNP36 | G/A | 36073 | TATAAAGTTGAACATTTGTTTATTATATTAAACAGAATAATGAGAAATTGGCTCTTAAATCGCAAAATTTTCTTATTTTCCATTATTCTATTCCAATCAAATCAATTCTAAAGATTATATTAGAAATCAATAAAAGAAAAAGTAAGTGGACCTAACTTATTGCATCATAACTATATCTACTATTCTGATAGTAAAATTCGATATAGATGAAATTGAAACAGTAGCTCTGCTTTTTCTTTCCTTTTCATATTTCGTTTTCTTTGGACTCCAAAAAATCTGTCGATATTTCTGATTAAATCTTCTTGCTCCTAGTTATTCTATAGGAATAAATAACTATTTCCCTCCTCCATAGAAAAGTTTATTCGAAGTCATAACATAAGACATAGAAGATACTTTTTAAATATCTTTCTTTGATTCCAGGACACAAAATCAATTTATATTGCTATTTATAGGTATATAAGATTTATATATAATAAGATTGATATATCTATCAGATCATG[G/A]CTTCCTGTACCAAATATTTTGGTATCGATACATACAATATTTTTATTCCGACAATGTAATGTAGAATAGGTGTGAGAAAAATACTTTCATTTTTAAATCCTTTCATTTAAAGTTTTCTATTTATTTAATATTGTATTGAATTTAATAATAGGAAAATTCATTCTCTTTCTTCTTTTCTGACAGATATAAAGTCAAGTAAATAAAAAAACTATTCGAAGCGTCTTTCTTGCTTTGACCTACAAAATATTCTGGTTTTTTTTTTATTTATTTTATCTGATTTCTTTTTTATTTTATCTGAAGTAAAAAGAAATATAACAAAAATGGCAAAAAGAAAATATAAGAAAGTTGGCGAGAAAGAATAAAATATAAAATAATAAAATATAGGATACTGTAACAGTTTAATGCATATAGAAATTAGAAGAATACGTAAAAATATTGATTTTTAGAAATCTTACGAACAAGATCCAAGAATAAGATTAGTTTGATAGAATGAGAGAAAT |
| SNP37 | T/G | 36374 | CTTGCTCCTAGTTATTCTATAGGAATAAATAACTATTTCCCTCCTCCATAGAAAAGTTTATTCGAAGTCATAACATAAGACATAGAAGATACTTTTTAAATATCTTTCTTTGATTCCAGGACACAAAATCAATTTATATTGCTATTTATAGGTATATAAGATTTATATATAATAAGATTGATATATCTATCAGATCATGGCTTCCTGTACCAAATATTTTGGTATCGATACATACAATATTTTTATTCCGACAATGTAATGTAGAATAGGTGTGAGAAAAATACTTTCATTTTTAAATCCTTTCATTTAAAGTTTTCTATTTATTTAATATTGTATTGAATTTAATAATAGGAAAATTCATTCTCTTTCTTCTTTTCTGACAGATATAAAGTCAAGTAAATAAAAAAACTATTCGAAGCGTCTTTCTTGCTTTGACCTACAAAATATTCTGGTTTTTTTTTTATTTATTTTATCTGATTTCTTTTTTATTTTATCTGAAG[T/G]AAAAAGAAATATAACAAAAATGGCAAAAAGAAAATATAAGAAAGTTGGCGAGAAAGAATAAAATATAAAATAATAAAATATAGGATACTGTAACAGTTTAATGCATATAGAAATTAGAAGAATACGTAAAAATATTGATTTTTAGAAATCTTACGAACAAGATCCAAGAATAAGATTAGTTTGATAGAATGAGAGAAATAAGTCTGAGGATCCACTAGTAAGGAGAGGGGGATCACTTGTTCCTTGAACAGTGCTTTTAAAAAATTCATCTATCTGATTGATTTGATGAGTCATAAAAAAATTCATGGTTCATATGGTTATTAAGACTATAAAAAAGAATAACCGAATTGAATTCATGAATTTACCTAAGTCAGGTTATGGACCGATAAAGAATTTTTTTCTTCGAAACCCATTAGAAATTAGAAAGGGCAGTGTACAAGAAATCAAATCATACATAAATGATAGAAGCTTCAAAGGCCCTGAAAATGCTATGAGGTGTT |
| SNP38 | A/C | 38837 | CTTGAAGAATCTTTTCCATTTTTTGGTTATGTATGGAAAGATAGAAATAAAATGACAACAATTTTAGGTATTCACTTAATCTTGCTAGGTATAGGTGCTTTTCTTCTAGTATTCAAGGCTCTTTATTTTGGGGGCGTATATGATACCTGGGCTCCGGGGGGGGGAGATGTAAGAAAAATTACTAACTTAACCCTTAGCCCAAGTGTTATTTTCGGTTATTTACTAAAATCCCCTTTTGGAGGAGAAGGATGGATTGTTAGTGTGGACGATTTGGAAGATATAATTGGAGGGCATGTATGGTTAGGTTCCATTTGTATACTGGGTGGAATCTGGCATATCTTAACCAAACCCTTTGCATGGGCTCGCCGTGCACTTGTATGGTCTGGAGAGGCTTACTTGTCTTATAGTTTAGGTGCTTTATCCGTTTTTGGTTTCATTGCTTGTTGCTTTGTCTGGTTCAATAATACCGCTTACCCTAGTGAGTTTTACGGGCCTACTGG[A/C]CCAGAAGCTTCTCAAGCTCAAGCTTTTACTTTTCTAGTTCGAGATCAACGTCTTGGGGCTAACGTGGGATCTGCTCAAGGACCTACCGGGTTAGGTAAATATTTAATGCGTTCGCCTACCGGAGAAGTTATTTTTGGAGGCGAAACTATGCGTTTTTGGGATCTGCGTGCTCCTTGGTTAGAACCTCTAAGAGGTCCAAATGGTTTGGACTTGAGTAGGTTGAAAAAAGACATACAACCTTGGCAAGAACGCCGTTCCGCAGAATATATGACCCACGCGCCTTTAGGTTCGTTAAATTCTGTAGGTGGCGTAGCTACCGAAATCAATGCAGTCAATTATGTCTCTCCTAGAAGTTGGTTAGCTACCTCTCATTTTGTTCTAGGGTTCTTCCTATTCGTAGGTCATTTATGGCACGCGGGAAGGGCTCGTGCAGCTGCAGCAGGATTTGAAAAGGGAATTGATCGTGATTTTGAACCCGTTCTTTCCATGACTCCTCTTAA |
| SNP39 | G/T | 39557 | TTGAAAAAAGACATACAACCTTGGCAAGAACGCCGTTCCGCAGAATATATGACCCACGCGCCTTTAGGTTCGTTAAATTCTGTAGGTGGCGTAGCTACCGAAATCAATGCAGTCAATTATGTCTCTCCTAGAAGTTGGTTAGCTACCTCTCATTTTGTTCTAGGGTTCTTCCTATTCGTAGGTCATTTATGGCACGCGGGAAGGGCTCGTGCAGCTGCAGCAGGATTTGAAAAGGGAATTGATCGTGATTTTGAACCCGTTCTTTCCATGACTCCTCTTAACTAACTGAGACAAGAGATCCAATAGTTAAATCGATCTTAAAAAGTATTCCTTTTTCCTTTCTTTTCAATTCATTTAGATCTAATCTATTTTTTTCTGGCTCGGCTATCCCATCTAGCCGAGCCATTTACATTTATGATACTGGGCCGGTCAAAACCAATAAAGACATAAATCTATTCATCGAGAAAAGGAGAGAGAGGGATTCGAACCCTCGATAGTTC[G/T]CAAAGAACTATACCGGTTTTCAAGACCGGAGCTATCAACCACTCGGCCATCTCTCCGAAAGATAATTTCTATTTTACTATTTTATTTTTATTCTGCCGAATAGAATAGAACACGGCCATCGGAGTTGATACCATCACTGTACTATAGAAAGATATTTGGAGAAAGATATTTGGTGTAAGTCGATAAGTCTATTTATCTATCTGTAATATAGAGATAGATGCAGGATCTAGCATGACCATTTGTGAAGTAAAAAACGACTCTTGACCCCATAAAATGGGCATAAAATGGGAAAGGGTGGTTATAAGTCATATAGAATCAATGGATTCATGGCACAATCCCCCCATGATGCATTTTTTTTTACAATTTTTTTTTGACTGATAGAGGGATCAAATGGTATAGTTCTTTTGTTGGTAGCTTGGAGGATTAGAAACATGACTATTGCTTTCCAATTGGCTGTTTTTGCATTAATTGTTACTTCATCAATCTTATTGATTAGTGTA |
| SNP40 | C/T | 39558 | TGAAAAAAGACATACAACCTTGGCAAGAACGCCGTTCCGCAGAATATATGACCCACGCGCCTTTAGGTTCGTTAAATTCTGTAGGTGGCGTAGCTACCGAAATCAATGCAGTCAATTATGTCTCTCCTAGAAGTTGGTTAGCTACCTCTCATTTTGTTCTAGGGTTCTTCCTATTCGTAGGTCATTTATGGCACGCGGGAAGGGCTCGTGCAGCTGCAGCAGGATTTGAAAAGGGAATTGATCGTGATTTTGAACCCGTTCTTTCCATGACTCCTCTTAACTAACTGAGACAAGAGATCCAATAGTTAAATCGATCTTAAAAAGTATTCCTTTTTCCTTTCTTTTCAATTCATTTAGATCTAATCTATTTTTTTCTGGCTCGGCTATCCCATCTAGCCGAGCCATTTACATTTATGATACTGGGCCGGTCAAAACCAATAAAGACATAAATCTATTCATCGAGAAAAGGAGAGAGAGGGATTCGAACCCTCGATAGTTCG[C/T]AAAGAACTATACCGGTTTTCAAGACCGGAGCTATCAACCACTCGGCCATCTCTCCGAAAGATAATTTCTATTTTACTATTTTATTTTTATTCTGCCGAATAGAATAGAACACGGCCATCGGAGTTGATACCATCACTGTACTATAGAAAGATATTTGGAGAAAGATATTTGGTGTAAGTCGATAAGTCTATTTATCTATCTGTAATATAGAGATAGATGCAGGATCTAGCATGACCATTTGTGAAGTAAAAAACGACTCTTGACCCCATAAAATGGGCATAAAATGGGAAAGGGTGGTTATAAGTCATATAGAATCAATGGATTCATGGCACAATCCCCCCATGATGCATTTTTTTTTACAATTTTTTTTTGACTGATAGAGGGATCAAATGGTATAGTTCTTTTGTTGGTAGCTTGGAGGATTAGAAACATGACTATTGCTTTCCAATTGGCTGTTTTTGCATTAATTGTTACTTCATCAATCTTATTGATTAGTGTAC |
| SNP41 | A/T | 39559 | GAAAAAAGACATACAACCTTGGCAAGAACGCCGTTCCGCAGAATATATGACCCACGCGCCTTTAGGTTCGTTAAATTCTGTAGGTGGCGTAGCTACCGAAATCAATGCAGTCAATTATGTCTCTCCTAGAAGTTGGTTAGCTACCTCTCATTTTGTTCTAGGGTTCTTCCTATTCGTAGGTCATTTATGGCACGCGGGAAGGGCTCGTGCAGCTGCAGCAGGATTTGAAAAGGGAATTGATCGTGATTTTGAACCCGTTCTTTCCATGACTCCTCTTAACTAACTGAGACAAGAGATCCAATAGTTAAATCGATCTTAAAAAGTATTCCTTTTTCCTTTCTTTTCAATTCATTTAGATCTAATCTATTTTTTTCTGGCTCGGCTATCCCATCTAGCCGAGCCATTTACATTTATGATACTGGGCCGGTCAAAACCAATAAAGACATAAATCTATTCATCGAGAAAAGGAGAGAGAGGGATTCGAACCCTCGATAGTTCGC[A/T]AAGAACTATACCGGTTTTCAAGACCGGAGCTATCAACCACTCGGCCATCTCTCCGAAAGATAATTTCTATTTTACTATTTTATTTTTATTCTGCCGAATAGAATAGAACACGGCCATCGGAGTTGATACCATCACTGTACTATAGAAAGATATTTGGAGAAAGATATTTGGTGTAAGTCGATAAGTCTATTTATCTATCTGTAATATAGAGATAGATGCAGGATCTAGCATGACCATTTGTGAAGTAAAAAACGACTCTTGACCCCATAAAATGGGCATAAAATGGGAAAGGGTGGTTATAAGTCATATAGAATCAATGGATTCATGGCACAATCCCCCCATGATGCATTTTTTTTTACAATTTTTTTTTGACTGATAGAGGGATCAAATGGTATAGTTCTTTTGTTGGTAGCTTGGAGGATTAGAAACATGACTATTGCTTTCCAATTGGCTGTTTTTGCATTAATTGTTACTTCATCAATCTTATTGATTAGTGTACC |
| SNP42 | A/G | 39560 | AAAAAAGACATACAACCTTGGCAAGAACGCCGTTCCGCAGAATATATGACCCACGCGCCTTTAGGTTCGTTAAATTCTGTAGGTGGCGTAGCTACCGAAATCAATGCAGTCAATTATGTCTCTCCTAGAAGTTGGTTAGCTACCTCTCATTTTGTTCTAGGGTTCTTCCTATTCGTAGGTCATTTATGGCACGCGGGAAGGGCTCGTGCAGCTGCAGCAGGATTTGAAAAGGGAATTGATCGTGATTTTGAACCCGTTCTTTCCATGACTCCTCTTAACTAACTGAGACAAGAGATCCAATAGTTAAATCGATCTTAAAAAGTATTCCTTTTTCCTTTCTTTTCAATTCATTTAGATCTAATCTATTTTTTTCTGGCTCGGCTATCCCATCTAGCCGAGCCATTTACATTTATGATACTGGGCCGGTCAAAACCAATAAAGACATAAATCTATTCATCGAGAAAAGGAGAGAGAGGGATTCGAACCCTCGATAGTTCGCA[A/G]AGAACTATACCGGTTTTCAAGACCGGAGCTATCAACCACTCGGCCATCTCTCCGAAAGATAATTTCTATTTTACTATTTTATTTTTATTCTGCCGAATAGAATAGAACACGGCCATCGGAGTTGATACCATCACTGTACTATAGAAAGATATTTGGAGAAAGATATTTGGTGTAAGTCGATAAGTCTATTTATCTATCTGTAATATAGAGATAGATGCAGGATCTAGCATGACCATTTGTGAAGTAAAAAACGACTCTTGACCCCATAAAATGGGCATAAAATGGGAAAGGGTGGTTATAAGTCATATAGAATCAATGGATTCATGGCACAATCCCCCCATGATGCATTTTTTTTTACAATTTTTTTTTGACTGATAGAGGGATCAAATGGTATAGTTCTTTTGTTGGTAGCTTGGAGGATTAGAAACATGACTATTGCTTTCCAATTGGCTGTTTTTGCATTAATTGTTACTTCATCAATCTTATTGATTAGTGTACCC |
| SNP43 | A/C | 39561 | AAAAAGACATACAACCTTGGCAAGAACGCCGTTCCGCAGAATATATGACCCACGCGCCTTTAGGTTCGTTAAATTCTGTAGGTGGCGTAGCTACCGAAATCAATGCAGTCAATTATGTCTCTCCTAGAAGTTGGTTAGCTACCTCTCATTTTGTTCTAGGGTTCTTCCTATTCGTAGGTCATTTATGGCACGCGGGAAGGGCTCGTGCAGCTGCAGCAGGATTTGAAAAGGGAATTGATCGTGATTTTGAACCCGTTCTTTCCATGACTCCTCTTAACTAACTGAGACAAGAGATCCAATAGTTAAATCGATCTTAAAAAGTATTCCTTTTTCCTTTCTTTTCAATTCATTTAGATCTAATCTATTTTTTTCTGGCTCGGCTATCCCATCTAGCCGAGCCATTTACATTTATGATACTGGGCCGGTCAAAACCAATAAAGACATAAATCTATTCATCGAGAAAAGGAGAGAGAGGGATTCGAACCCTCGATAGTTCGCAA[A/C]GAACTATACCGGTTTTCAAGACCGGAGCTATCAACCACTCGGCCATCTCTCCGAAAGATAATTTCTATTTTACTATTTTATTTTTATTCTGCCGAATAGAATAGAACACGGCCATCGGAGTTGATACCATCACTGTACTATAGAAAGATATTTGGAGAAAGATATTTGGTGTAAGTCGATAAGTCTATTTATCTATCTGTAATATAGAGATAGATGCAGGATCTAGCATGACCATTTGTGAAGTAAAAAACGACTCTTGACCCCATAAAATGGGCATAAAATGGGAAAGGGTGGTTATAAGTCATATAGAATCAATGGATTCATGGCACAATCCCCCCATGATGCATTTTTTTTTACAATTTTTTTTTGACTGATAGAGGGATCAAATGGTATAGTTCTTTTGTTGGTAGCTTGGAGGATTAGAAACATGACTATTGCTTTCCAATTGGCTGTTTTTGCATTAATTGTTACTTCATCAATCTTATTGATTAGTGTACCCG |
| SNP44 | A/T | 40598 | AAAATGTTGTATTTTCCGGTACATCATTATGGATTGGATTAGTTTTTTTGGTAGGTATCCTTAATTCTCTCATCTCTTGAACCTAGTTGTTTCAGATACAAAAATGAAATGAAAACGACCCCCCCCCCCCCGAATCCTTTCGGGTTGCGAAACACATTAAAATTCAATATAAGCCTAAGTTCCGAAAATGCAAAATACAAATAAAGAAAACAAAAAATTAAAGGGAGGGGTCAAATCAAACTTTTTTTTGTAATTTGTAAATGTAATTAAATGTAAAATTTAGTCTTTAGTCTAATGGGATAAAAAAATTATTATAATTATAAATGAATCTATTAGAAATAAAATAGAAATAAAAAAAATAATAAATAGAAATAAAAAAAATAATAAAATTTATATTAATTTATATTTATAATTTATATTAATTTATATTTATATATATTTATATATATTTATATGTAATTATGTAATAAATTATAGTATAATAAATTAATATATAATAT[A/T]TATAGTATAATAAATAATATAAATTAATATAAATAATTATTAATTATAAATAATTAATATTAAATTGAATATTCAAATTAATAATATAATAATTATAAATAATTATAATAATAATCGCCCTGAAATATCGAGCATAGCTGTTCACAAATATGATCCAGATATTGTGTATCAAGAACGAAAAAAAAAAATGCGGATGCGGATATAGTCGAATGGTAAAATTTCTCTTTGCCAAGGAGAAGACGCGGGTTCGATTCCCGCTATCCGCCCAAGCCCAAGGTAAGGTAATTTTTTAAATAGGATAAAGGATTCAGTATAGTTGACCATGATAGTATAGTGATTCTATCCCCTATCTTCTTTCTTTTCTTTCCCCCTGCTCCAAAAAAGAAAAAAAGAAACTCCTCCCTCTAAAAATAAAAAAAAAAATGTTGCGGAGACGGGATTTGAACCCGTGACTTCAAGGTTATGAGCCTTGCGAGCTACCAAGCTGCTCTACCCCGCGT |
| SNP45 | A/T | 41012 | TATATTAATTTATATTTATATATATTTATATATATTTATATGTAATTATGTAATAAATTATAGTATAATAAATTAATATATAATATATATAGTATAATAAATAATATAAATTAATATAAATAATTATTAATTATAAATAATTAATATTAAATTGAATATTCAAATTAATAATATAATAATTATAAATAATTATAATAATAATCGCCCTGAAATATCGAGCATAGCTGTTCACAAATATGATCCAGATATTGTGTATCAAGAACGAAAAAAAAAAATGCGGATGCGGATATAGTCGAATGGTAAAATTTCTCTTTGCCAAGGAGAAGACGCGGGTTCGATTCCCGCTATCCGCCCAAGCCCAAGGTAAGGTAATTTTTTAAATAGGATAAAGGATTCAGTATAGTTGACCATGATAGTATAGTGATTCTATCCCCTATCTTCTTTCTTTTCTTTCCCCCTGCTCCAAAAAAGAAAAAAAGAAACTCCTCCCTCTAAAAATA[A/T]AAAAAAAAATGTTGCGGAGACGGGATTTGAACCCGTGACTTCAAGGTTATGAGCCTTGCGAGCTACCAAGCTGCTCTACCCCGCGTAATGAAGCGAAGAACTGGAAACTAATCTAATGGACAAACAAAGATTGAATGTGCCCCTCTACCATATCTGTACAAATAGAATAGCGCATTTATACAGAATGGTAAAGGGACCCCTCTACGATCGATGATCATAAAAATAACTAGAAAAATGAAGGGAAATTTTTAATCCTTACCAACTCGATCTTGTTGCCCCCGGTAACAAACATGCATGAACCATTTCACGAAGTATGTGTCCGGATAGTCCAAAGTCTCGATAGTTAGCTCTCGGTCTTCCAGTCGAAAAACAGCGTCGATGAAGACGTGTCGGTGCACTATTTCGCGGTGGGGATTGTAACTTTCCATGAATTTCCCATTTATCACTCAACGACGGAACTTTGCTTATTTCTTTTTTTGAGGATCGACGAATCAAATGAT |
| SNP46 | G/A | 46433 | TGAGAGTTGGCCGAAATGAGCACTAAATACTTTTCGAGAAATCTCCTCCAAATCACTGGTATGGCTATCGAAATCGTGAGCATCAGCATGTAGGTTCCAGATCCAAGTGGTAGTATCTGGTCCTTTAGCTATTGTTCTTGAGAAATGACCCGGTCTAGCCCATTCCTCGAAAGAAGTTTTGACGGGATCCCTATCTACCAAAATTTTTACTTCTGGTTCCGGCGAACGAATAATCATTGAGTCCTCCTCTTTCCGGACAACGCATATAAAGAGACCCGCCAACAATCGAGTCAAGCAATTAGTGAACCTATGAGAGATACTTATAATTAGTTTCTTTCTCTTCTATCTCCCATTTATCTTATCTAGTTTCTTTAGTTATTCACTAGAGCAATTATGATCTGGAAGTCGATCCAGGGCAAGTGTTCGGATCTATTATGACATAGCCACAAGGCGCTCAACGGACCTTTCTGGGCTTTGAATTAACGCAAAAACAATTTTTT[G/A]TGCAACCTAGTATATTCCTATCTTAATTAAAAGTTTCTAGCCGAGACTACTTTATATTTTACTTATGTTTTTTTTTTTTTTTTATTTTTACATAGAACATATTACTATTACTCTATTCCAAATCGGGTGAGAGGCCATTAGTCATTAGTAAGAGACATTCCAGGATCTATTAGGCTATCTTTTATTCGTTTTAATAACAGAAAAGAAAATATAGAAAATATATATTCTCTGCTGTTTATATCTGTGTAGTGTTTATTCCTATGAAATACCAGACGAAAGGGGACGATCTTAGAAGGGGTATAATGAAATTCATGAAATTTTTTGATTGGTTCTTCCCAGAGAAACCATCCTTTTATTTTATTTGACTGATAGGGATAACAAACAAATAATTATAACAAATTTTTTATAAGAAATAAAAAAATATACCAAATACTAAAAAAATACTAAATTAAATAATAAATACAAAAATACAAAGGGAATGCTCTTATCTTATTCAAAAC |
| SNP47 | A/G | 46445 | GAAATGAGCACTAAATACTTTTCGAGAAATCTCCTCCAAATCACTGGTATGGCTATCGAAATCGTGAGCATCAGCATGTAGGTTCCAGATCCAAGTGGTAGTATCTGGTCCTTTAGCTATTGTTCTTGAGAAATGACCCGGTCTAGCCCATTCCTCGAAAGAAGTTTTGACGGGATCCCTATCTACCAAAATTTTTACTTCTGGTTCCGGCGAACGAATAATCATTGAGTCCTCCTCTTTCCGGACAACGCATATAAAGAGACCCGCCAACAATCGAGTCAAGCAATTAGTGAACCTATGAGAGATACTTATAATTAGTTTCTTTCTCTTCTATCTCCCATTTATCTTATCTAGTTTCTTTAGTTATTCACTAGAGCAATTATGATCTGGAAGTCGATCCAGGGCAAGTGTTCGGATCTATTATGACATAGCCACAAGGCGCTCAACGGACCTTTCTGGGCTTTGAATTAACGCAAAAACAATTTTTTGTGCAACCTAGT[A/G]TATTCCTATCTTAATTAAAAGTTTCTAGCCGAGACTACTTTATATTTTACTTATGTTTTTTTTTTTTTTTTATTTTTACATAGAACATATTACTATTACTCTATTCCAAATCGGGTGAGAGGCCATTAGTCATTAGTAAGAGACATTCCAGGATCTATTAGGCTATCTTTTATTCGTTTTAATAACAGAAAAGAAAATATAGAAAATATATATTCTCTGCTGTTTATATCTGTGTAGTGTTTATTCCTATGAAATACCAGACGAAAGGGGACGATCTTAGAAGGGGTATAATGAAATTCATGAAATTTTTTGATTGGTTCTTCCCAGAGAAACCATCCTTTTATTTTATTTGACTGATAGGGATAACAAACAAATAATTATAACAAATTTTTTATAAGAAATAAAAAAATATACCAAATACTAAAAAAATACTAAATTAAATAATAAATACAAAAATACAAAGGGAATGCTCTTATCTTATTCAAAACGCCTTGTGATCT |
| SNP48 | A/C | 48237 | AATGACAGATCACGGCCATATTATTTAAAGCTTGTGGTAAGAAGGGGTTTCGTTCTAGTGCCCGAAAATAATATTCCAAAGCTTTTGTATGTTCTCCATTACTTGTGTGAATAAGGCCTATATTATAGAGTATATAACTTCGATCATAGGGATCAATTTCTAGCCGCATAGCTTCATAATAATTCTGTAAAGCTTCTGCATAATTTCCTTCGGATTGAGCAGACATCCGTTACGGTCGTCATTCGATTCAAAGAATCTCCGTTCCAGAACCGTACGTGAGATTTTCATCTCATACGGCTCCTCCCTTAATGTGCATAATGAGAATAAAACGTAGAATAAAAAGGGGAGTGAAATATTCTCATTATGAACTGAGCGGGGCTAGTGTTTTTACAAGAAATCTCTAGCCAACCTTCCTGCAAAAGATCTTTTCTTAACATCAAGCATGTTGATACTAGAGAAAAATATAATATTAAGCTAACTCCAACAATTTCTTTGTCCTC[A/C]ATCTTCCTTAATTTCTAGGAATTAGTCACTTCAACAGTCTTCGATGGTTATACGGGTATCCAAAGTACGAACGAGATGGATGTTTGTTGTCCCAACCATTCTTCTTAGTCCCAATCCCAATAAAGAAAAGGGATAATTTCTAACAAAGTTTTCGTGTTGTTGATTCCTAGGCGTAGTGTTTCTTCCTCTATGCCGCCTATTGGTACTAGTGGGGTAGGGTTAACCTGCACTGCAATACAGAACCCATAGTCATAGGTGTAACCTTACGTTCAATGCTAGAATTCACAATTGAAGCATCTGAGGCTGCATTAATCGGGGATACCCGACAGAAGGAATTGTTTTATTTATAAACTTATAAACTTCACCTTCAACAAGCGTAGAGTTATTTCAAATCTTTCTATCCCGACTAATGTGTCTTTCTCCTAAGACTTGAAGCGGTAAATAAAAAAAAATCAAATCACACCATCTCTGTAATAAGTAAATGCCTCTTTTTCTCCCGA |
| SNP49 | C/G | 48390 | CAATTTCTAGCCGCATAGCTTCATAATAATTCTGTAAAGCTTCTGCATAATTTCCTTCGGATTGAGCAGACATCCGTTACGGTCGTCATTCGATTCAAAGAATCTCCGTTCCAGAACCGTACGTGAGATTTTCATCTCATACGGCTCCTCCCTTAATGTGCATAATGAGAATAAAACGTAGAATAAAAAGGGGAGTGAAATATTCTCATTATGAACTGAGCGGGGCTAGTGTTTTTACAAGAAATCTCTAGCCAACCTTCCTGCAAAAGATCTTTTCTTAACATCAAGCATGTTGATACTAGAGAAAAATATAATATTAAGCTAACTCCAACAATTTCTTTGTCCTCAATCTTCCTTAATTTCTAGGAATTAGTCACTTCAACAGTCTTCGATGGTTATACGGGTATCCAAAGTACGAACGAGATGGATGTTTGTTGTCCCAACCATTCTTCTTAGTCCCAATCCCAATAAAGAAAAGGGATAATTTCTAACAAAGTTTT[C/G]GTGTTGTTGATTCCTAGGCGTAGTGTTTCTTCCTCTATGCCGCCTATTGGTACTAGTGGGGTAGGGTTAACCTGCACTGCAATACAGAACCCATAGTCATAGGTGTAACCTTACGTTCAATGCTAGAATTCACAATTGAAGCATCTGAGGCTGCATTAATCGGGGATACCCGACAGAAGGAATTGTTTTATTTATAAACTTATAAACTTCACCTTCAACAAGCGTAGAGTTATTTCAAATCTTTCTATCCCGACTAATGTGTCTTTCTCCTAAGACTTGAAGCGGTAAATAAAAAAAAATCAAATCACACCATCTCTGTAATAAGTAAATGCCTCTTTTTCTCCCGAAGTTGTCGGAATTATTCGTAATAAGATATTGGCTACAATTGAAAAGGTCTTATCAATAAAATTTCCAGTTATCCGGGATCTAGGCATAAGTAGCAATCCATTTTATAATTTCTTTTAATTACCTCTCATGAGAAAACGATCCCACAAAAAAGT |
| SNP50 | T/C | 53433 | TTCTTATTATTCAATGAGCATCTTGTATTTCATAAAAATTAGGAGCAATATAATCTTTACGTAAGGGCCACCCTATCCAACTTTCCGGCATTAAGATACGTTTCAGACGCGGATGATTATCATAAAGGATTCCCAACATATCATAAGATTCCCTTTCTTGAAAATCCGCACTTTTCCAAACCCAGAAAACAGACGGAATTCTAGGATTCTTCCTTGGGGCAAATACCTTTATACATACTTCTTCTGGTTGATCTATACCATACTCTATTCTCGTAAGATGATATACGCTAGCTAACAGCCCGCCCGGCGCTACATCATAGGCACATTGCAACCGCAGATAATTGTAACCATATACATATAAAATGACAGCAATGGAATGCCAATCTGCGGGCTTTATTTGTAAAGTCTCTATTCCTTGGTAATCAAAACCCAAAGATCTATGAACCAGTCCGTGTTTGACTAGCCAAGCAGACAAACGACCCTGCATCTTTTTTCTCCCT[T/C]CCGCATTTTTATTTGTATAATAATTTTAATAATTATTTTTTGTATAATATAAGTATTTCACATTCACGATGAAATTTATGAAGATTGGTTTGCCTTTTTTTGTTATTCTGTACAAATGAGCTCTTCCTCTAATTTACTAATTCGTGGGACGATACTGACCTTTTTTTATATTTGAAAAATGTTTCAGGAGGGATCTTTGAAGTAGACGGTGGTTGATATAGTAATCCTTGATCATAATTTCCAGTATGAGTAGCGCGTTCAATATTAAACTTGTGATTGGTAGTAAAACACCGATTCCCGGGTTGAGACCTAATTCGATCTTCATAAATTTCTCGAGATAGTTTTTTACGAAGTTTTGTTATAGCATCTATAACCGCCTCCGGTTTAGGTGGACAGCCCGGCAAATAGACATCTACAGGAATTAGCTTATCGACTCCCCGAACAGTACTATAAGAGTCGGTACTGAACATCCCCCCTGTAATTGTACATGCCCCCATAGC |
| SNP51 | T/C | 56954 | TCTAGCAAAACCGCCCATCAGAGCCATCGTTAACCATTGGTCATTAAGGCGTATTCTCAAAATACCGATATCGACTGCTGTGGCAATAGGCGCATGATTTGATAATACGCCAATTTGTCCACTATTAGTAGATAAAATGATTTCTTTCACTTCTGAATCCCAAACAATTCGATTTGGGGTCAGTACACAAAGATTTAAGGTCATTTCTTCAAGTTGTTCTCCATTTCTAAATTCGTAGCCTTCGCAGTAGCTTCATCAATATTACCTACCAAATAAAAGGCCTGCTCAGGTAGACTATCTAATTCTCCGGAAAGGATCAATTTAAATCCTCTAATAGTTTCTGCTAAACCGACATATTTCCCCGGGGAACCCGTAAATACTTCTGCTACAAAAAAGGGTTGTGATAAGAAACGCTCAATTTTTCGTGCTCTTGCTACAGTTAAGCGATCCTCTTCGGATAATTCGTCTAACCCAAGGATAGCTATAATGTCTTGAAGTTC[T/C]TTGTAACGTTGTAAAGTTTGCTTAACTCTTTGCGCAGTTTCATAATGTTCCTCACCAACGATCTGAGGTTGGAGCATAGTTGACGTTGAATCTAAAGGATCTACTGCTGGATAGATCCCTTTAGCAGCTAATCCTCTTGATAGTACAGTAGTAGCATCTAAATGTGCAAATGTCGTGGCAGGAGCAGGGTCAGTCAAATCGTCCGCAGGTACATAAACTGCTTGAATAGAAGTTATGGACCCTTCTTTTGTAGAAGTAATTCGTTCTTGTAAAGAACCCATTTCGGTACTAAGGGTAGGTTGATAACCCACAGCGGAAGGCATTCTACCTAATAAGGCGGATACTTCGGATCCTGCTTGGACGAAACGGAAGATATTGTCGATAAATAGAAGTACGTCCTGTTCATTAACATCTCGGAAATATTCCGCCATAGTTAGGGCAGTTAAGCCAACTCTCATACGAGCTCCCGGGGGTTCGTTCATCTGACCATAGACTAGAGC |
| SNP52 | T/G | 58263 | ATGTTGCGCGAGTATCTACAGGACCTAAATCATCAACAGGTTCTCCAAGCACGTTGAAAATTCGTCCTAGAGTCGCTCCGCCGACTGGAACACTGAGAGGACCTCCCGTGTCAATCACTTCCATTCCTCTCATTAGACCGTCTGTAGCACTCATAGCTACAGCCCGAACTCGATTATTTCCTAATAATTGTTGTACTTCACAAGTCACATTAATTTCTTGACCAACAGTATCCCGGCCCTTAACTACCAGAGCGTTGTAAATATTAGGCATCTTTCCCGGGGGAAAAGCTACATCTAGCACCGGCCCAATGATTTGAGTGATGCGCCCCAGGTTCTTTTTTTCAAGTGTGGAAACTCCCGGACCAGAAGTAGTAGGATTGATTCTCATAATAAAATAATAAAAATAATAAAAAAAATATGTCGAAATTTTTTTTTTTTCAAAATTTTCTAGTCCAAAATAAATGTTCGACGGCAAGTTGATCGATTAATTCAGTAAAAAA[T/G]CAAAAATGGAAGTTAGTACTGGATTTCGTTGATACTAGCTAACAAGTCCAATTCAATTGTTTACTTACTTTCAATGGGTGAGTTTTCAAGTTCAACTAACTCATTTTGAAAATATAAATAAATATTAAGTAAATATTAAGAATATTAAGTAAGTGGATGCATAAGAAACATAAAAAAAGGCTTCAGGAAGCCTTTCATTTGTCTATCATTATAGATAATACTATTCGTATTTTCTATGGAATTCGAACCTGAACTTATGATTCATTAATTATTTCATGGGCCCTTCTTTTTTTATTTCAGCATATCGACTTACACCTAGCTATTATTATTCTTTTCTTTTTTTCATAGATGAATTCTGCCGATTTTCACATCTCGGATTTACATATACAACATATATCACTGTCACGAGGAAATTTCTTATTATTTAGATATTTCGATTCAAAAAAGTTAAGGATTAGAAACTTGAAAAAAAAAAAAACAAGGATTGGGTTGCGCCATAG |
| SNP53 | C/A | 58264 | TGTTGCGCGAGTATCTACAGGACCTAAATCATCAACAGGTTCTCCAAGCACGTTGAAAATTCGTCCTAGAGTCGCTCCGCCGACTGGAACACTGAGAGGACCTCCCGTGTCAATCACTTCCATTCCTCTCATTAGACCGTCTGTAGCACTCATAGCTACAGCCCGAACTCGATTATTTCCTAATAATTGTTGTACTTCACAAGTCACATTAATTTCTTGACCAACAGTATCCCGGCCCTTAACTACCAGAGCGTTGTAAATATTAGGCATCTTTCCCGGGGGAAAAGCTACATCTAGCACCGGCCCAATGATTTGAGTGATGCGCCCCAGGTTCTTTTTTTCAAGTGTGGAAACTCCCGGACCAGAAGTAGTAGGATTGATTCTCATAATAAAATAATAAAAATAATAAAAAAAATATGTCGAAATTTTTTTTTTTTCAAAATTTTCTAGTCCAAAATAAATGTTCGACGGCAAGTTGATCGATTAATTCAGTAAAAAAT[C/A]AAAAATGGAAGTTAGTACTGGATTTCGTTGATACTAGCTAACAAGTCCAATTCAATTGTTTACTTACTTTCAATGGGTGAGTTTTCAAGTTCAACTAACTCATTTTGAAAATATAAATAAATATTAAGTAAATATTAAGAATATTAAGTAAGTGGATGCATAAGAAACATAAAAAAAGGCTTCAGGAAGCCTTTCATTTGTCTATCATTATAGATAATACTATTCGTATTTTCTATGGAATTCGAACCTGAACTTATGATTCATTAATTATTTCATGGGCCCTTCTTTTTTTATTTCAGCATATCGACTTACACCTAGCTATTATTATTCTTTTCTTTTTTTCATAGATGAATTCTGCCGATTTTCACATCTCGGATTTACATATACAACATATATCACTGTCACGAGGAAATTTCTTATTATTTAGATATTTCGATTCAAAAAAGTTAAGGATTAGAAACTTGAAAAAAAAAAAAACAAGGATTGGGTTGCGCCATAGA |
| SNP54 | G/T | 58371 | TGTCAATCACTTCCATTCCTCTCATTAGACCGTCTGTAGCACTCATAGCTACAGCCCGAACTCGATTATTTCCTAATAATTGTTGTACTTCACAAGTCACATTAATTTCTTGACCAACAGTATCCCGGCCCTTAACTACCAGAGCGTTGTAAATATTAGGCATCTTTCCCGGGGGAAAAGCTACATCTAGCACCGGCCCAATGATTTGAGTGATGCGCCCCAGGTTCTTTTTTTCAAGTGTGGAAACTCCCGGACCAGAAGTAGTAGGATTGATTCTCATAATAAAATAATAAAAATAATAAAAAAAATATGTCGAAATTTTTTTTTTTTCAAAATTTTCTAGTCCAAAATAAATGTTCGACGGCAAGTTGATCGATTAATTCAGTAAAAAATCAAAAATGGAAGTTAGTACTGGATTTCGTTGATACTAGCTAACAAGTCCAATTCAATTGTTTACTTACTTTCAATGGGTGAGTTTTCAAGTTCAACTAACTCATTTT[G/T]AAAATATAAATAAATATTAAGTAAATATTAAGAATATTAAGTAAGTGGATGCATAAGAAACATAAAAAAAGGCTTCAGGAAGCCTTTCATTTGTCTATCATTATAGATAATACTATTCGTATTTTCTATGGAATTCGAACCTGAACTTATGATTCATTAATTATTTCATGGGCCCTTCTTTTTTTATTTCAGCATATCGACTTACACCTAGCTATTATTATTCTTTTCTTTTTTTCATAGATGAATTCTGCCGATTTTCACATCTCGGATTTACATATACAACATATATCACTGTCACGAGGAAATTTCTTATTATTTAGATATTTCGATTCAAAAAAGTTAAGGATTAGAAACTTGAAAAAAAAAAAAACAAGGATTGGGTTGCGCCATAGATATGAAAGAGTATACAATAATGATGTATTTGGTGAATCAAATACCATGGTCTAATAAAAAAATTCTGATTAGTTGATAATATTGGTTGATAACTTTGTGAAAGATTC |
| SNP55 | A/C | 59244 | AGGATTGGGTTGCGCCATAGATATGAAAGAGTATACAATAATGATGTATTTGGTGAATCAAATACCATGGTCTAATAAAAAAATTCTGATTAGTTGATAATATTGGTTGATAACTTTGTGAAAGATTCCTGTGAAAGGTTTTATTAACTCCTAATTCATGTCGAGTAGACCTTATTGTTGTGAGAATTCTTAATTCATGAGTTGTAGGGAGGGACTTATGTCACCACAAACAGAGACTAAAGCAAGTGTTGGGTTCAAGGCTGGTGTTAAAGATTATAAATTGACTTATTATACTCCTGAATATGAAACCAAAGATACTGATATCTTGGCAGCATTCCGAGTAACTCCTCAACCTGGAGTTCCGCCTGAGGAAGCAGGAGCTGCAGTAGCTGCTGAATCTTCTACTGGTACATGGACAACTGTGTGGACCGATGGGCTTACCAGTCTTGATCGTTATAAAGGACGATGCTACCACATTGAGCCCGTTGCCGGAGAAGAAA[A/C]TCAATTTATTGCTTATGTAGCTTACCCCTTAGACCTTTTTGAAGAAGGTTCTGTTACTAACATGTTTACTTCCATTGTGGGTAATGTATTTGGGTTCAAAGCCCTACGCGCCCTACGTCTGGAGGATTTGCGAATCCCTCCTGCTTATACAAAAACTTTCCAAGGGCCGCCTCATGGCATCCAAGTTGAGAGAGATAAATTGAACAAGTATGGTCGCCCTCTATTGGGTTGTACTATTAAACCTAAATTGGGGCTATCCGCTAAGAATTACGGTAGAGCAGTTTATGAATGTCTACGCGGTGGACTTGATTTTACCAAAGATGATGAGAACGTGAACTCCCAACCATTTATGCGTTGGAGAGACCGTTTCTTATTTTGTGCCGAAGCAATTTATAAAGCACAGGCTGAAACAGGTGAAATCAAAGGACATTATTTGAATGCTACTGCAGGTACATGCGAAGAAATGATCAAAAGGGCTGTATTTGCCAGAGAATTAGGAG |
| SNP56 | G/A | 61328 | TTTTTTAGAAGAAGAGAAATATTACTTTTCTCGATGCGAATTTGACACAATATAACAAATTCCTTTTCTATTTCTATTTAGACAGACTTATTCTAATTGAAAAATAATGGAAAACGGGATTCCGGCATAAACATATCATATAGAGTGAAGCGATACTCCAGGTTCTTACAAAAAAAGCAAAAGATAAGCGTTTTTTTTAATACCCCCCCTATTAGTTAATAATCCTATAATAATCCTAGTAATCGGATCTATATACTTATATAGAGAGGAAATGAAATATTTCAATGATTTTTCATCGAATGACTATTCATCTATTTATTTAAATGTAAATAGCAGCAAGAAAGAAAGTTCTATGGAAAAACGGCGGTTCAATTCGATCTTATCCAATGTGGAATTAGGATACAGGTGTAGGCTAAGTCAATCGATAGATAGTTTCAGTCCTCTTGAAAATACCAGTGTAAGCGAAGACCCGATTCTAAATGATACAGATAAAAACACCC[G/A]TAGTTGGAGTAATAGTGACAGCTCTAGTTACAGTAATGTTGATCGTTTAGTCGGCGCCAGAGACATTCGGAATTTCGGCGCTGATGAAACTTTTTTAGTTAGGGATAATAATAGGGACAGTTATTCCATATATTTTGATATTGAAAATCAAGTTTTTGAGATTGACACTGATCATTCTTTTCTGAGTGAATTAGAAAGTTATTTTTATAGTTATTGGAATTCTAGTTCTCTGAATAATGGGTCTAGGAGTGGCGATTCCTACTATGATCATTATATGTATGATACTAAATATAGTTGGAAGAATTACATCAATAGTTGCATTGACAGTTATCTTCGTTCTCAAGTCTGTATTGATATTTATATTTTAACTGGCAGTTACAATTACGGCGAAAGTTCCATTTATAGTTACATTTGTGGTGAAAGTGGAAATAGTAATGAAAGCGAGAGTTCCAGTCTAAGAACTAGTACGAATGGTAGCGATTTAACTAGAAGAGAAAGTT |
| SNP57 | T/A | 63614 | TTTTACTAAAAAAAAATCGACGGATTCTAAATTATAACGAATTTTGGGGGAATTTATAATCGGAAAAACTCTTTATTTTTTTTTTTATTTTATATTAAATATATTAAAATATTTTCTATTTTCTATTAAAGTAAAGTTAAAAAAATGAAAAAAGAAAAATGAAAGTTATAAGACAAGAAAAAAAAAGATAATAAAAGAAGAATAACAAATAAATGGATTATCATATTTCATGTAGAAATATGAATAAGTCATTTAGTTAGTTCTACGCTCTTTGCACTTTATTATATTATATATACTCACTTAGATATACTTAGTATAATTATATACGAATTTTAATGATTATAAGAAATCTTTCTAATAACTAAATAACTAATAAATAACTAATAATAGAATAATTAATATAAATATAATTATAATTAATTTAAATTATTAATTTAAATTATTAAATTAATAGAATAAAATAATAATTAATAATAAAAAAAAAATAATTATAATAATAA[T/A]AAATAATAATAAAAAAAAAAAAATAATGAAAATATATAAATATATTAAAATATAAATATATTAAATATATATTAAAATATATTAAATATATATTAAAATATTCAAAATAATATAATATAAAAATAATATAATTATAATATATAATAAAAAAAAAAAAATGAAATAACAGGTACAAATATTAAATCGAGGTGCCCATTCTATGACAATTCTCAACAGCTTACCCTCCATTTTTGTGCCTTTAGTAGGCTTAGTATTTCCGGCAATTGCGATGGCTTCCTTATCTCTTCATGTTCAAAAAAACAAGATTTTTTAGATCCAATTAGGTCTAACGGGGGTAGATTTCATTTCTTTTTTTTTTCAAGACTTAGACTTGGATCATAATATAATACAGATATAATACGGATATCTCTTTAGTTTAGTATAATATGATATAACATACGAGCAGCTCCCTTCAAACGTAAATGAAAATGAAAGGGTTCTTATGCAGACGTAAATATGAT |
| SNP58 | C/T | 69651 | AATGCTATGAATGACCCAATATCGAATACTGGTAATAATATCAGCAAAAGAACGTTCTCCTGTGCTTCCAGACATACTGAGCTCCACATATTCTTGTACAATCAAAAGGGATCGATTCTGTAAAAGATGAGATCAGTAAATGCAATTTCACTGAAATTGAATCTTTGTGAGATCGTCAATATTGTACCAAAGGTTTCTTTAAAGTATACCGAATCAGTATAGCTATCCTTCTTCTGACGCAGCAACGCAACTTCAATCAGTATCGAAATGAAATCTTAGATAATCCCTTTCTTCTTTTCTTTTTCTTGTTGCTGTAGAACTGTGTACCATAAGATAAGGTCGGGTAAAATGCGAATTCAACGGGTTACAATAATAATTCATGGAGGAGATTATCATATTTCCGCAATTCAATTAGATGTGAATGGATGTGAAATCAAAAACTTTTGTGCTTTTGTGGTTATAGAAGAGTTTTTTGTTGGAATCCCAACTCCCCCCCCCCC[C/T]TTTTTTTTTTTCATTTTGTTTTTCATTTTAAGGAATTGGTTAATCATCTATTAAATTAACAAGTACCAATAGTAGAGAATATTCGATGAAAGAGAAAGAAGGAGAACAAGGAAAAAATAATTGTAATAATCAATATTCGTGATGCACGTATTTGTGTATTAGATTTAAAAGGTCTTTCTTGACACTTAACTACATGTTTTTTCGTTTTAATATTAATATAATATGGAACGAAAAATGTAAAATCTAGAAAAGAAAAGGATAATTGAAATTACTAGTCTTAGAATCTAGTTGTACAAAAAACAAAAAAAAAAAGAAAGATTTTCTTTTTTGAGTGATCTGATCGTGCGATACTTTTTTTCTTCTTATTTTATTGTATTGGATTGGAGATATTATGTGAATGAATCAACTAATAAATCTAATAAGTTAAATTCAAATTTAAAATAAAAATAGAAAAAAGTAAAAAAAAAAAAGTAAAAAGGAAAAGGTATTCTAAAGAAAAA |
| SNP59 | C/A | 71295 | ACTATAACTAGTTATTTCGGTTTTCTACTAGCGGCTTTAACTATAACCTCAGTTCTATTTATTGGTCTGAGCAAGATACGACTTATTTGAAATAAATTGAATGAACAATTTATAAAAAAATTTATAAAAAGAAAAGGAAATTTTTCGGCAGTATTCCATCTATTCTCTAGTTCTTTACCGTGTTAATTTTCAATTCTTGCTTATTGAGATTTTTCTTATTGAGATTTATGGGCGATATGGATTAATATTTAAGGATAGATATTACCTTTCTTTCCCCCCCCCTTTTTTTTTTTCAAATAAATTGAAATGATTGAAGTTTTTCTATTTGGAATCGTCTTAGGTCTAATTCCTATTACTTTGGCCGGATTATTCATAACTGCATATTTACAATACAGGCGTGGTGATCAGTTGGACCTTTAATTAATTAATTAATTAACACCTTGTTTTTTTGACCTCCTCTTTTCTTTAATCCACAGGAGGTCAAATTCAGATTGCTCTTC[C/A]ATTTTTTCTGTATAAAATTTGACCTAACAAAACAAGAACAGAATCACGCTCTGTAGGATTTGAACCTACGACATTGGGTTTTGGAGACCCACGTTCTACCGAACTGAACTAAGAGCGCTTTCTTATCCCAATCACAAAAAAATCAGACTGTAAGTAAAAAGATTCTTTTTTTAACTACTCCAATATATCTTGAATGCCTATACTATCATATATAATATGATAAAAATAAAAGATTAAGTATGGCCAAGTTTAATTGATCTCAATTGATCCCTCGTTATTACTCAGAGACGAAGTAATAGGTAGGGATGACAGGATTTGAACCCGTGACATTTTGTACCCAAAACAAACGCGCTACCAAGCTGCGCTACATCCCTTTCAATTGGTTTACAATGTTATTGTAAAGAATACCCGTTTTGTTTTCCACATACTTATTTCATTTTCTCCATTGATATACTTTTTCTTTTTGATCTCATATCATATATTATATTCATATATTATAT |
| SNP60 | C/A | 73568 | TATATATATATTTAAATATAAAATAAACAAGCAAAATCCTATTTCTTAATTCTAAATCATTTTTGATCCAATTGAACGAAATAGGATTTTCGAAATAAGGAATAAACAAACCATGGATAAATCCAAACGGCCTTTTCTTAAGTCCAAGCGATCTTTTCGTAGGCGTTTGCCCCCGATCCAATCGGGGGATCGAATTGATTATAGAAACATGAGTTTAATTAGTCGATTTATTAGTGAACAAGGAAAAATATTATCTAGACGGGTGAATAGATTGAGTTTAAAACAACAACGATTAATTACTATTGCTATAAAACAAGCTCGTATTTTATCTTCGTTACCTTTTCTTAATAATGAAAAACAATTTGAAAAAAAGCGAGCTGGTCACTATAACTACTGATCTTAGAACCAGAAAAATAGGCTTACTCCTCAATTGAATCAAAATTCAAATCCGAATTTAATTTAAACCTAGATTAATGTTTTGTTCAAAAAATCCGAAAATC[C/A]AAATTTGAGTGTCGTGTCGTAAGAAAAAAAACGAGTTGGGTAAGAAGAAATTTTTTTTATTGAATGTTTTTGTTCAGTTTGACTACTTTATACTTGATTATATTATCATCATATTTTATCATACTAATTTCTATCATATTTTATCATACTAATTTCTATTCTACCTTCCCGGAATTCCTTCTTCAGGGAATTCCATGTAAAATATTTCATAGATTCTTTCCAATTTACTTATTTGATAATGTCATTGGAAATCATATAAAGGCAATTCCTATTTAATATAGCTATTTGTGCAAGTATTTTACGATTAAGAAGCAACTGTCTCTTGTACAGATTATTTATTAATATACTATAACTATAGGATACCCTATTCTCACGAATTACTGCATTTATCCGAGTGACCCACAAACGACGAAAATTTCTTTTTTGCCTATCTCTATCCCGATGGGCCAAAGCCAAAGCTCTTATTTTTTGTTGAATAATAGTTCGAGTAAGTCTTGAAT |
| SNP61 | A/C | 73795 | TTATTAGTGAACAAGGAAAAATATTATCTAGACGGGTGAATAGATTGAGTTTAAAACAACAACGATTAATTACTATTGCTATAAAACAAGCTCGTATTTTATCTTCGTTACCTTTTCTTAATAATGAAAAACAATTTGAAAAAAAGCGAGCTGGTCACTATAACTACTGATCTTAGAACCAGAAAAATAGGCTTACTCCTCAATTGAATCAAAATTCAAATCCGAATTTAATTTAAACCTAGATTAATGTTTTGTTCAAAAAATCCGAAAATCCAAATTTGAGTGTCGTGTCGTAAGAAAAAAAACGAGTTGGGTAAGAAGAAATTTTTTTTATTGAATGTTTTTGTTCAGTTTGACTACTTTATACTTGATTATATTATCATCATATTTTATCATACTAATTTCTATCATATTTTATCATACTAATTTCTATTCTACCTTCCCGGAATTCCTTCTTCAGGGAATTCCATGTAAAATATTTCATAGATTCTTTCCAATTT[A/C]CTTATTTGATAATGTCATTGGAAATCATATAAAGGCAATTCCTATTTAATATAGCTATTTGTGCAAGTATTTTACGATTAAGAAGCAACTGTCTCTTGTACAGATTATTTATTAATATACTATAACTATAGGATACCCTATTCTCACGAATTACTGCATTTATCCGAGTGACCCACAAACGACGAAAATTTCTTTTTTGCCTATCTCTATCCCGATGGGCCAAAGCCAAAGCTCTTATTTTTTGTTGAATAATAGTTCGAGTAAGTCTTGAATGAGCCCCGCGAAAGCTTGATGCGAATAAACGAATTTTTGTTCTACGCCTCCGAGCTATATATCCTCGTCTAATTCTGGTCATTGAATAAACGAAACTTTGATAAATAACTAATTGATTTCCTTTCTTTCAGTTATTCTTTTCCCCTTTTCTATAATAACAAAACGGATTCTTCCAGTGTATAAAATAATAATTCCAACGGCTTTTGCTACTATAACCTTCCCAACCA |
| SNP62 | A/C | 73912 | TTAATAATGAAAAACAATTTGAAAAAAAGCGAGCTGGTCACTATAACTACTGATCTTAGAACCAGAAAAATAGGCTTACTCCTCAATTGAATCAAAATTCAAATCCGAATTTAATTTAAACCTAGATTAATGTTTTGTTCAAAAAATCCGAAAATCCAAATTTGAGTGTCGTGTCGTAAGAAAAAAAACGAGTTGGGTAAGAAGAAATTTTTTTTATTGAATGTTTTTGTTCAGTTTGACTACTTTATACTTGATTATATTATCATCATATTTTATCATACTAATTTCTATCATATTTTATCATACTAATTTCTATTCTACCTTCCCGGAATTCCTTCTTCAGGGAATTCCATGTAAAATATTTCATAGATTCTTTCCAATTTACTTATTTGATAATGTCATTGGAAATCATATAAAGGCAATTCCTATTTAATATAGCTATTTGTGCAAGTATTTTACGATTAAGAAGCAACTGTCTCTTGTACAGATTATTTATTAAT[A/C]TACTATAACTATAGGATACCCTATTCTCACGAATTACTGCATTTATCCGAGTGACCCACAAACGACGAAAATTTCTTTTTTGCCTATCTCTATCCCGATGGGCCAAAGCCAAAGCTCTTATTTTTTGTTGAATAATAGTTCGAGTAAGTCTTGAATGAGCCCCGCGAAAGCTTGATGCGAATAAACGAATTTTTGTTCTACGCCTCCGAGCTATATATCCTCGTCTAATTCTGGTCATTGAATAAACGAAACTTTGATAAATAACTAATTGATTTCCTTTCTTTCAGTTATTCTTTTCCCCTTTTCTATAATAACAAAACGGATTCTTCCAGTGTATAAAATAATAATTCCAACGGCTTTTGCTACTATAACCTTCCCAACCACGATTTTTTCTTTCGAGGCATTTCACCTCGAAATAAGAAACTGTATTGATACTAGGTATCAAACAAAAATATAATAAAAAAGTAGTAAATAAAAATATAGAAATAGATAAATAAATA |
| SNP63 | A/G | 73941 | CGAGCTGGTCACTATAACTACTGATCTTAGAACCAGAAAAATAGGCTTACTCCTCAATTGAATCAAAATTCAAATCCGAATTTAATTTAAACCTAGATTAATGTTTTGTTCAAAAAATCCGAAAATCCAAATTTGAGTGTCGTGTCGTAAGAAAAAAAACGAGTTGGGTAAGAAGAAATTTTTTTTATTGAATGTTTTTGTTCAGTTTGACTACTTTATACTTGATTATATTATCATCATATTTTATCATACTAATTTCTATCATATTTTATCATACTAATTTCTATTCTACCTTCCCGGAATTCCTTCTTCAGGGAATTCCATGTAAAATATTTCATAGATTCTTTCCAATTTACTTATTTGATAATGTCATTGGAAATCATATAAAGGCAATTCCTATTTAATATAGCTATTTGTGCAAGTATTTTACGATTAAGAAGCAACTGTCTCTTGTACAGATTATTTATTAATATACTATAACTATAGGATACCCTATTCTC[A/G]CGAATTACTGCATTTATCCGAGTGACCCACAAACGACGAAAATTTCTTTTTTGCCTATCTCTATCCCGATGGGCCAAAGCCAAAGCTCTTATTTTTTGTTGAATAATAGTTCGAGTAAGTCTTGAATGAGCCCCGCGAAAGCTTGATGCGAATAAACGAATTTTTGTTCTACGCCTCCGAGCTATATATCCTCGTCTAATTCTGGTCATTGAATAAACGAAACTTTGATAAATAACTAATTGATTTCCTTTCTTTCAGTTATTCTTTTCCCCTTTTCTATAATAACAAAACGGATTCTTCCAGTGTATAAAATAATAATTCCAACGGCTTTTGCTACTATAACCTTCCCAACCACGATTTTTTCTTTCGAGGCATTTCACCTCGAAATAAGAAACTGTATTGATACTAGGTATCAAACAAAAATATAATAAAAAAGTAGTAAATAAAAATATAGAAATAGATAAATAAATAATAGAAATAATAGTGGGTTCCATCGTTTC |
| SNP64 | G/A | 75739 | ATATTTAATTGAAATTGAAAGTCCGGTAAGAAAAAAAATATTCAATCGTGATTTGCGTGAAATTTCTGCTATTTCTTATGCAACTGCTACAAGATCAACAATTCCATGAGCTTGGGCTTCTGCTGCTGACATAAAAACATCTCTTTCCATGTCTTCGGATACAACCCATAAAGGTTTTCCCGTTCTTTGTGCATAAATCCTTGTGAGGATTTCGCGCAGTTTCAGTAGTTCTTCTGCTTCCAGGACAAATTCTCCTATTTGGGCCTCATAAAAACCAGCAATAGGTTGATGAATCATTACCCTGATGATATAAGAAAAAAATGGCTATTCTATCTCGCATAATAAATAAAAAGATGACGAGAAGAGATAAAGAATAATAAGAAAAAAAAAGATAGAATTGAACAACCGTACAGGCATTGTTTACGCATTGCATACGGCTCCACAATGGAATTCACTTTTACTTACCTTTTTTTATCGAATAAAAACATAGAGTTTATCAG[G/A]TTTAAATCAGTAAATGATCCAATAACCACCCTTTCCTTGTCTTTGGAGGAGTTAAAAAATAAAACAAAATACTATGGTGGTCCCGTTGCTTTATTTCATCAGTGATTTAGCAATCCCAAAGTTTTTTTTTTTTATTTGAAAAATAAATCTTTTTTTCGTACTCTTTTATTTTTTTATTTTTATAACATAAATAATGTTAAGAGCCCTCCGGTGCGAAAACAAAAAAGTTTGTGACGCTGAAATAGGCCCCTGATAGAATAAAATCAGAAATACCCTTACTTAATATCTCATACTACTCTTTCGATACATAATCTAATGTTTAAAAAAACAATTCTTATATCTATATCGAATTCGAACTGCCATGCTATTATTACTTAATATTTATATTTCAATATTTCATATGGCGAAGGCATAGTTTTCTTTTTTCTTTCAAATAAAAACCCATTGGCGCCAAGCATGAGGGAATGCTAAACGTTTGGTAATTTTTCCTCCAACTAGGA |
| SNP65 | C/T | 76499 | AAAATCAGAAATACCCTTACTTAATATCTCATACTACTCTTTCGATACATAATCTAATGTTTAAAAAAACAATTCTTATATCTATATCGAATTCGAACTGCCATGCTATTATTACTTAATATTTATATTTCAATATTTCATATGGCGAAGGCATAGTTTTCTTTTTTCTTTCAAATAAAAACCCATTGGCGCCAAGCATGAGGGAATGCTAAACGTTTGGTAATTTTTCCTCCAACTAGGATAAAAGATGCCATTGAAGCAGCTAATCCCATGCATACTGTTTGTACATCTGGTCGCACAAATTGCATAGTATCATAAATAGCTATTCCGGGTATTACCCATCCCCCAGGAGAGTTTATAAACAAATACAAATCTTTAGTCTCGCTCTCTATACTGAGATATACCATAAGACCAATAAGTTGATTCGAGATCTCGCTATCAACATCTTGACCTAAAAAAAGTAATCTTTCTCGATAAAGTCGGTTGATTGAGATAAATTT[C/T]TATCTTCTAGGAACCGTACGTGCACCTTTTGATGCATACGGTTCAACAAAAATCGACGAAAATAAAATAAAGAATCAATGTGTAGATTCCAACCTTCCTTTTTTGATAGTGAGTACTTTTTAACTTAACTCTTTTTTTTTCTAATGTCTAATGCTAATGAAAGGGCTTTTCTTCCAGTTTTCAAGAAAGATGAGTTTTAACCCGTTTACCTATAAAATAAAAAAAGGTTTCCCTATAAAGATAAAAAAAAATTATTAAACTTATCAAACAAACTTCTCATTGATGTATTTTTTCATCGAGATTCAATTCAAATCACGACGGCATTATCTTGTTCCTGAATGGGTTTCTTCAATTCTTTTAGGTTTGTGCTCTACTCCGAGTAAAGATTTGCCCGATTTTTATTTGCACATATAGAGCAAATGCCCCAATACCGCGTCTTTTTGTTACAACTTCTTTTTTTTTTATTTAATTCATTTCACGTCTTCTGCCAAATATTTGAC |
| SNP66 | C/A | 77468 | ATTCATTTCACGTCTTCTGCCAAATATTTGACATATTCATTATTATATTATTCGATTGAGTATGATTAGCAGTTTGAAATTATTTCAACTTCATAATTATTTCAACTTCATAAATAGAATATGATACAAGCAGTAATCGTAATAGATATATTACCAATTGGGTTCTTCTAAACGGAGCCTGGATACTTCATTTTTTTGGTCCAAACAAGCCAACCATAAATTATTCTAATTGATATATATAATATTAATCTGAGTACCTCAAAAGCATAGATCTAATTGGACTTCATTGCATTTCACGCTCCAAATTTTGGATGATTTAATCAATCTTTCTTGGGCGAAACCGAGGATATCTCAATCGGGGGAGAGAACGGGGGAATCCCGTATGACCCAATATATCTGACAAGTCGCACTATACGTCAATCCAAATGGAATCGTCTTCCCCAGGATTTCGAAAAGGGACTTTTGGAACACCAATAGGCATTAAATGACAGAAAAATTTT[C/A]AGTACTCTATTTCACTTTGATGTGAAAACGTAACAATGAATTTATTGTCTTATAACAATGAATTTATTGTCTTAATAATAATAACCTTTATAATATTTTATACATGGATTCGATAAGTTTATATTTATATATTTAATATATTTATATATATATAATATATAAATATATATATAAAAATATATATATAAAATAAAAAAAAAAGGGAAGACAAAACCAATAGAGTTCAATTCTTACGAATAGGTACTTTGAATGATGAACAAATCTCTATGCATTCGCTCATATAAAATAGAGTCAACCCCCCATTGCGTATTGGTACTTATCGGGTATAGAATAGATCTGGTTCTCTTTTCTTTGTTCCTACAAACGGAATTGTTACATTATTACTAAAATAAAAAAGAAAAAGAATAGAAAAAAATATTAATTCTTTCTCCGAGATAATCTACTTAAAAGGAGAGATCCATAACATAGTTTTTCCAGTGCAATAAAGTTACATAGTGTCT |
| SNP67 | T/A | 77640 | CGGAGCCTGGATACTTCATTTTTTTGGTCCAAACAAGCCAACCATAAATTATTCTAATTGATATATATAATATTAATCTGAGTACCTCAAAAGCATAGATCTAATTGGACTTCATTGCATTTCACGCTCCAAATTTTGGATGATTTAATCAATCTTTCTTGGGCGAAACCGAGGATATCTCAATCGGGGGAGAGAACGGGGGAATCCCGTATGACCCAATATATCTGACAAGTCGCACTATACGTCAATCCAAATGGAATCGTCTTCCCCAGGATTTCGAAAAGGGACTTTTGGAACACCAATAGGCATTAAATGACAGAAAAATTTTCAGTACTCTATTTCACTTTGATGTGAAAACGTAACAATGAATTTATTGTCTTATAACAATGAATTTATTGTCTTAATAATAATAACCTTTATAATATTTTATACATGGATTCGATAAGTTTATATTTATATATTTAATATATTTATATATATATAATATATAAATATATATA[T/A]AAAAATATATATATAAAATAAAAAAAAAAGGGAAGACAAAACCAATAGAGTTCAATTCTTACGAATAGGTACTTTGAATGATGAACAAATCTCTATGCATTCGCTCATATAAAATAGAGTCAACCCCCCATTGCGTATTGGTACTTATCGGGTATAGAATAGATCTGGTTCTCTTTTCTTTGTTCCTACAAACGGAATTGTTACATTATTACTAAAATAAAAAAGAAAAAGAATAGAAAAAAATATTAATTCTTTCTCCGAGATAATCTACTTAAAAGGAGAGATCCATAACATAGTTTTTCCAGTGCAATAAAGTTACATAGTGTCTATTTTTCGTTAATAAAGGGGTATTTCCATGGGTTTGCCTTGGTATCGTGTTCATACCGTCGTATTAAATGATCCCGGTCGTTTGCTGTCTGTCCATATAATGCATACAGCTTTAGTTGCTGGTTGGGCCGGTTCGATGGCTCTTTATGAATTAGCAGTTTTTGATCCCTCTG |
| SNP68 | A/T | 77644 | GCCTGGATACTTCATTTTTTTGGTCCAAACAAGCCAACCATAAATTATTCTAATTGATATATATAATATTAATCTGAGTACCTCAAAAGCATAGATCTAATTGGACTTCATTGCATTTCACGCTCCAAATTTTGGATGATTTAATCAATCTTTCTTGGGCGAAACCGAGGATATCTCAATCGGGGGAGAGAACGGGGGAATCCCGTATGACCCAATATATCTGACAAGTCGCACTATACGTCAATCCAAATGGAATCGTCTTCCCCAGGATTTCGAAAAGGGACTTTTGGAACACCAATAGGCATTAAATGACAGAAAAATTTTCAGTACTCTATTTCACTTTGATGTGAAAACGTAACAATGAATTTATTGTCTTATAACAATGAATTTATTGTCTTAATAATAATAACCTTTATAATATTTTATACATGGATTCGATAAGTTTATATTTATATATTTAATATATTTATATATATATAATATATAAATATATATATAAA[A/T]ATATATATATAAAATAAAAAAAAAAGGGAAGACAAAACCAATAGAGTTCAATTCTTACGAATAGGTACTTTGAATGATGAACAAATCTCTATGCATTCGCTCATATAAAATAGAGTCAACCCCCCATTGCGTATTGGTACTTATCGGGTATAGAATAGATCTGGTTCTCTTTTCTTTGTTCCTACAAACGGAATTGTTACATTATTACTAAAATAAAAAAGAAAAAGAATAGAAAAAAATATTAATTCTTTCTCCGAGATAATCTACTTAAAAGGAGAGATCCATAACATAGTTTTTCCAGTGCAATAAAGTTACATAGTGTCTATTTTTCGTTAATAAAGGGGTATTTCCATGGGTTTGCCTTGGTATCGTGTTCATACCGTCGTATTAAATGATCCCGGTCGTTTGCTGTCTGTCCATATAATGCATACAGCTTTAGTTGCTGGTTGGGCCGGTTCGATGGCTCTTTATGAATTAGCAGTTTTTGATCCCTCTGACCC |
| SNP69 | G/A | 85118 | TGGCATAGGGGTTACATCCCGGACGAAGCTTAATAATATACCACTTCTGCGAATAGCTCTTAATGCTGCATCTCGCCCGAGACCAGGACCTTTTATCATGACTTCTGCCCGTTGCATACCTTGATCCACTACTGTTCGAATAGCATTTCCTGCTGCGGTTTGAGCTGCAAATGGCGTTCCTCTTCTTGTGCCCCTGAATCCACAAGTGCCGGCGGAGGACCAAGAAATCACTCGACCCCGTACATCTGTAACGGTCACAATAGTATTGTTGAAACTTGCTTGAACATGAATAACTCCTTTTGGTATTTTATGCGCACTCTTACGTGAACCAATACGTCCATTCCTGCGTGAACCAATTCTTGGTAAAGGTTTTGCCATATTTTATTATCTCATAAATATAAGTCAGGGGTCTATGGATATATCCATTTCATGTCAAAATAGATCCTTTTTTTTATTTATACATTTTTTTATTTGTACATCAGATCCTTTAGAGCGTTTCC[G/A]CTTAGCAAGATTATCCTTGTCTTTGTTTATGTCTCGGGTTAAAGCAAATTACTATAATTCGCCCCCGTCTACGTATCAGTCGACATTTTTCACAAATTTTACGAACAGAAGCTCTTATTTTCATATTTGCCGTCCCTTAAATTTTGAATATACATACTTTTTTTGAAGAAAATAAGTTTCTTTAAACTTTGAAATCGCGAATTGTATCCCTTGAAAGTGATGAAAGTGAAAGGACTTTTTAAGTTGAAAAAAAAAAACTGACTAATCATTCAAATCTTTGTTTGTTACGGAGTCTATAAATTAGAAGTGCTCTGGTCGAATTCTAAATACTTAACACTATTTCTGAGCAGTATAGGTATAAAAAAACTATGTTGGATCCTTCCTGAAGCATAACCTAAAACCAGATCTTCATTATCTAAAGGAACTTGGAACATACTATTGAAAAAGTGATTCAGAAATTTAACCTTCCCAAATTGAATCCATTTTTTGTTCTTTCATTC |
| SNP70 | G/T | 85340 | AGAAATCACTCGACCCCGTACATCTGTAACGGTCACAATAGTATTGTTGAAACTTGCTTGAACATGAATAACTCCTTTTGGTATTTTATGCGCACTCTTACGTGAACCAATACGTCCATTCCTGCGTGAACCAATTCTTGGTAAAGGTTTTGCCATATTTTATTATCTCATAAATATAAGTCAGGGGTCTATGGATATATCCATTTCATGTCAAAATAGATCCTTTTTTTTATTTATACATTTTTTTATTTGTACATCAGATCCTTTAGAGCGTTTCCGCTTAGCAAGATTATCCTTGTCTTTGTTTATGTCTCGGGTTAAAGCAAATTACTATAATTCGCCCCCGTCTACGTATCAGTCGACATTTTTCACAAATTTTACGAACAGAAGCTCTTATTTTCATATTTGCCGTCCCTTAAATTTTGAATATACATACTTTTTTTGAAGAAAATAAGTTTCTTTAAACTTTGAAATCGCGAATTGTATCCCTTGAAAGTGAT[G/T]AAAGTGAAAGGACTTTTTAAGTTGAAAAAAAAAAACTGACTAATCATTCAAATCTTTGTTTGTTACGGAGTCTATAAATTAGAAGTGCTCTGGTCGAATTCTAAATACTTAACACTATTTCTGAGCAGTATAGGTATAAAAAAACTATGTTGGATCCTTCCTGAAGCATAACCTAAAACCAGATCTTCATTATCTAAAGGAACTTGGAACATACTATTGAAAAAGTGATTCAGAAATTTAACCTTCCCAAATTGAATCCATTTTTTGTTCTTTCATTCCATCTAAAATCCCCTTCAAGTATCAATTAATGTAATAGGAATGATATTAGAAACCTCTTCTTTCTCTTTTTTTTTTTTTCACAAATAAGAAGTTGGGATACAATTCGGATATCCGAAAGATTACCATATATAACACAAGATTTCTCCACCGATTCTTTCTAGTCGAGCTTCTCGGTCGGTCATTATACCCCGAGAAGTAGAAAGAATTACAATACCCATCCC |
| SNP71 | A/G | 86048 | AACATACTATTGAAAAAGTGATTCAGAAATTTAACCTTCCCAAATTGAATCCATTTTTTGTTCTTTCATTCCATCTAAAATCCCCTTCAAGTATCAATTAATGTAATAGGAATGATATTAGAAACCTCTTCTTTCTCTTTTTTTTTTTTTCACAAATAAGAAGTTGGGATACAATTCGGATATCCGAAAGATTACCATATATAACACAAGATTTCTCCACCGATTCTTTCTAGTCGAGCTTCTCGGTCGGTCATTATACCCCGAGAAGTAGAAAGAATTACAATACCCATCCCGCCCAAAATTCTAGGAATTTTTTGATAGTTAGAATAGATTCGTAGACCAGGTCGGCTAATCCGTTTTAAATTTAGACTAGTTCTATAGGGTCCCTTCCTCTTCCTTCTATGTCGTAGGGTTAAAACCAAAAATTTTTTGTTGCCTTCCTGGTGTTTCCTGACATTTTCAATAAAACCTTCTCGTAAAAGTATTTTAATAATGTTTTC[A/G]GTGATGTTAGTGAATGCTATTCGAACGGTTCCTTTTCTATTCATGTCAGCATTTCGTATAGAGGTTATTATCTCAGCAATAGGATCCCTGCCCATGATAAACTAAATTTTGAATTTCTCTCTAGTTTTGATATAATCAACATACTTTTTTTTTTTCTTTTTTTTTTTTTGAATTACTTATTTTATTAAAGGTATATGCGTGAAACACAATTTACTAATGAAATTCTATTTCATTTTTATTCAAATACTATAACTATACAATAAAAACTATATAAAAAATACTATAACTATATACTAGAACTGTATCATGGTCTCATCTTAATTTTAAATGGTATTATCTTCTATTTTATTTAGAATTTTTTATTTTATAATACTTCAGGCGCTAATGAAACTATTTTAGTAAAATTTAACTGTCTCAATTCTCGGGCGATTGCACCAAAAATTCGAGTTCCCTTTGGATTTCCTTCTTGATCAATGACAACTGCAGCATTGTCATCATAT |
| SNP72 | T/C | 87496 | CTACTCCACTCATTTCATAAAGTATTCTACCTGGTTTAACGACAGCCACCCAATATTCAGGAGATCCTTTCCCCGAACCCATACGTGTTTCCGTAGGTCTTAAAGTAACTGGTTTGTCGGGAAATATGCGTACCCATATTTTTCCACCGCGGCGTGCATTTCGTGTCATTGCCCGTCGTCCCGCTTCTATTTGTCTAGATGTAATCCAAGCGGGTTCAAGTGCCTGAAGAGCATATTTGCCGAAACAAATACGATTACCTCGAAAAGCTATTCCTTTCATTCTTCCTCTATGTTGTTTACGGAATCGGGTTCTTTTTGGGTTATAGTTGATGGTTGTTTCTCAATTCCATCTCTACTACAGAACCGGACATGAGAGTTTCTTCTCATCCAGCTCCTCGCGAATGAAATGTATATATATGTGTTTGATGAATAATATACTAAATCATGGGATTCTTTGAGATTTCACTTAATTTATTTACTTAATCTAATCTATTTCTAAT[T/C]TATTTATTAGATTATTTTCTATTTTATTAGATTATTAGAATAGAAATAGAAATTGGTATATATATATATATATATATATATTTTTTTATTATAATTATGATATCAGATCGCTAAAATTTAGAAATCCAATAATCTAAAAAATTTCGCGGGCGAATATTTACTCTTTCAATATTTATTTTATTTGTTTTGTAGAGCTAATCCACGATCCTTCAGAATAAATGGATTGTCTTTTGGTTCCTTTCGCTATCCTCCCAATAAATCATTAAGATTTGTTTTCAATAAAATCTTATGTATTTACAGGTTCCGTCGTTCCCATCGCTTCTCCATTAATGGTTAGGTCTTAATTTTACAATGGAGCCTCTAATTTGATTTGTTCTTGAGTCAATCTTCTCAGTCTTTATTGGCTCGAGGCTCTTGATTTTTTGTTTTATGAATGAATTAGTTTCATTATAGATCAATTGGTATTGATGCTTTAGTACATTAGCCTTTATCTGATGATT |
| SNP73 | T/A | 87578 | ACGTGTTTCCGTAGGTCTTAAAGTAACTGGTTTGTCGGGAAATATGCGTACCCATATTTTTCCACCGCGGCGTGCATTTCGTGTCATTGCCCGTCGTCCCGCTTCTATTTGTCTAGATGTAATCCAAGCGGGTTCAAGTGCCTGAAGAGCATATTTGCCGAAACAAATACGATTACCTCGAAAAGCTATTCCTTTCATTCTTCCTCTATGTTGTTTACGGAATCGGGTTCTTTTTGGGTTATAGTTGATGGTTGTTTCTCAATTCCATCTCTACTACAGAACCGGACATGAGAGTTTCTTCTCATCCAGCTCCTCGCGAATGAAATGTATATATATGTGTTTGATGAATAATATACTAAATCATGGGATTCTTTGAGATTTCACTTAATTTATTTACTTAATCTAATCTATTTCTAATTTATTTATTAGATTATTTTCTATTTTATTAGATTATTAGAATAGAAATAGAAATTGGTATATATATATATATATATATATAT[T/A]TTTTTATTATAATTATGATATCAGATCGCTAAAATTTAGAAATCCAATAATCTAAAAAATTTCGCGGGCGAATATTTACTCTTTCAATATTTATTTTATTTGTTTTGTAGAGCTAATCCACGATCCTTCAGAATAAATGGATTGTCTTTTGGTTCCTTTCGCTATCCTCCCAATAAATCATTAAGATTTGTTTTCAATAAAATCTTATGTATTTACAGGTTCCGTCGTTCCCATCGCTTCTCCATTAATGGTTAGGTCTTAATTTTACAATGGAGCCTCTAATTTGATTTGTTCTTGAGTCAATCTTCTCAGTCTTTATTGGCTCGAGGCTCTTGATTTTTTGTTTTATGAATGAATTAGTTTCATTATAGATCAATTGGTATTGATGCTTTAGTACATTAGCCTTTATCTGATGATTCATAGACCTTACATATCGGAATCATAGATCATTGATATTTTTTTCTCTCTTTCTTTCACCCTTCCATTTATCCGCATAATTT |
| SNP74 | C/A | 88908 | ATAGAAATATAATTCGAATTGATTCTTTTTTTTTTTAATAATAAAAAAAAAAAGAATCAAAGAGTTTGTCATTTTTGTTTTATCAATAGATAGAATGTAAAGACAAGTCAAGTAAGGGTTTATTATTTTTTGTCTAGAAATGTCCAAATTTTTATGCCTAATACCCCATAAATGGTTCTAACTGTATATGAACAATAATCAATTTTAGCCCGAATGGTTTGTAGAGGAACCCTACCCTCTCTAATCCACTCGACGCGTGCAATTTCTTTTCCGTCAAGACGCCCCGCAATTTGTACTTGAATTCCTTTTGTATCCGCCTGTTCAGTTAATTCAATAGCTTTTTTCATTGCTTTGCGAAATGAAACTCTATTCTTTAATTGTCCGGCTATAAATTCTGCAAGAATATTAGGGTGCCCATAAGGGTTTGAAATTCTTGTAATAGCAATATTGAGTTTTCGGTTCACACAATTTAGTTCTTTTTGTACATTTATCTGTAATTT[C/A]TCGATTCGTTTAGGTTTACTTTCTATTAATAATTTTGTGAATCCCATATATATTATTACTTGAATCACATCAATTCTTTTTTGAATCTCTATACATGCAATTCCCTCAACACCAGAAGATATTTTTGTATTGTTTTTTACATAATTCTTGATACAGTTTCTTATTTTTTGATCTTCTTGTAGACCCTCAGAGTAATTTTTTGGTTGAGCAAACCAAAGAGAATAATGACTTTGGGTTGTACCAAGTCGGAAACCTAGTGGATTTATTTTTTGTCCCATAATCCCCCACTACTTACTATACATATCATGAAATGTCGCATCTGTGGATTTCGTTTTTTTTATCCACCCCCGGTTTTTTAAGCATATGTTATATTCTTCATATTCTTCATATTCTTCATATAAAGATATATTTTTTAATACAATAGTTATATGACAGGTCGATCTTTTTATCAGATAACCCCGCCCTCGAGCCTGAGGTTTTAATTTTTTCACAGTAGCACC |
| SNP75 | C/A | 117643 | TATTTTATTTAATTTTTATTATTTATTAAATATTAATATTAAATTCAAAATTATATTAAATAATTAAAATTAGAATTATATTCTATATTAAATAATTCTAAAATAAAAAAATTCAATATTAAAATTAATAAAATTTAAAATAAAGTTTTACAATTTGTAAATAAAATTGAATTTTTTTTTTACAAATTGTAAAAAGAATAAATTAATAAAAAGATTAATGCATAAAATAAATATAATAAGAGATAAGAAGAGATGCGACCGCTTCCTACATATTTTATACCCTCTCCTACAAAGAAACTGGTAACACCAACTCCATTGGTAATTCCATCAATTATTCGTTTATCAAAAAAATGAATTAATTCAGCTAATTTTCTTATACCTCCAATAAAAGATATTTCATAAAAAGCATCTATGTAACCTCGATTATAGGACCAGTTATATATGACGTTTATTATTTTGTCCCAAAGAATTCTCTTAGAACCTTTTTTAGCGAGCGAATT[C/A]AAGAAATTCAAATTTTGTAACGATGAATAAACAGGCTTATATAAAGAGGACGCTATAAATATTCCGAAAAAAGCTATACTGACTGAAAAAATTGCATTTGTTACAAATTCATACCAATCCACAGAATTCTTTGAATTTTGATGCAAAAGGTTTAAAGACGGAGTTAACAGTTTTGACAATATATCCACCTCCATTCCTTTTTGATTGAAAGGAATTTGATTGAAAGGAATTCCTATGGCTCCAATAAACAAAGCAAATAGTACCAAGACAAGCATAGGAAATAACATAGTATTATCCGATTCATGAGGATAGGCAAAAATCCTTTTAGTGTTAAAATTTTTAATAGTAATAAAAGGCCACATCATCTTTCTTACATTACCATCAATTTGATATGTGTTCTTCCAAAAAAAAGAAGCCCTTTCGTTATTATTCGTTGTTAAGAAAGGTAATAAACCCAAACTTTCGTTAAGCATTTTTGATCCTTCTTTACCCCATAAAGA |
| SNP76 | G/T | 118922 | TAAGAATCATATTTTGACTCTTATCTGGAGAATAACCAACAATAGCTTCCATTGAATGAATAATGGATCCAGATCCTAAAAACAATAATGCTTTCGAATAGGCATGAGTAATCAAATGAAATAAAGCGACTCGATAAGACCCCATACCTAGAGCTAACATCATATAACCTAATTGAGACATTGTAGAATAGGCTAAACTTCTCTTAATATCTTTTTGAGCAAGAGCTAAAGTAGCTCCTAAAAATACTGTTATTATACCTATCAAAGCTATTAGATTCATTATGTAAGGTATGACTACAAAAAGAGGAAAAAGTCGAGCTACAAGAAAAATTCCTGCCGCTACCATAGTAGCAGCATGTATTAGAGCCGAAATAGGAGTAGGCCCTTCCATGGCATCCGGTAACCATACATGGAGAGGAAATTGCGCCGATTTAGCAATTGCGCCAGAAAATACTAGAAAGGCACACAAAGTAACAAATAAAAAATGAACCTGATTATTA[G/T]AAATCAAGTTATTCAATATTTTGAACAAATCCCGAAATTCGAAACTGCCCGTTATCCAATAAATGCCCAAAATTCCCAATAATAAACCAAAATCCCCTACACGATTAGTTACAAAGGCTTTTTGACAAGCATTCGATGCAATAGGTCGTGTGAACCAAAACCCTATTAATAGATAAGAACACATTCCAACCAACTCCCAAAAAATATAAATTTGTATCAAATTAGAACTAGTAACTAATCCCAACATTGAAGTATTGAAAAAACTCATATAAGCAAAAAATCTCAAATAGCCTTGATCATGAGACATATAATTGTCACTATAAAAAAGAACCATAATTCCAACTGTAGTAATTAATACTGACAAAATAGAAGTAAGTGGGTCAATCAAGTGTCCGAATTCTAAAGAAAAATCATTATTGATAGTCCACGACCATATATATTGATAAATAAAACTGCTATTTATTTGGTGAATAGACAAGTCGATTGAAAAAATCATGACT |
| SNP77 | C/A | 120378 | ATATTAATTACATTCCATAATTATTAAATATTAATTACATATACATAATTATTACATTAATTATTAATATTAATTTAATTACATAATTACAAGTTTTTATATACATAGAGAAAAGATAAAGAATTTTAACAATTATAAGAAAAGTAGTATTTTTTTTCTTGATAGAAATAAAAAAAGAAAGATGATTTTATCGGATCCTTACTGGATCAAAATAATTATTTTTTTGAGTTCTTTTATTTTTTTTTTTATTCAGAATTATTAACTAGTGGATTTTGGAACGGATTTATTGCCTTTCATTATGTTTGTAGAAAAGACTATCATATTTGAAACTTTTCCTTTACATAAGATAAAAGCAAAGAATTACTAAAATTTTTTTCCTTTTTTAACAAATTAATAGCCTCATTCAATTTGAAAATTTTAATTCAATTAGATATACTTTTTTTTCGAGTTTGACCAATTACTAGAAAAATGAAGTTAAAGTCTTTTTATTAGGATAAATC[C/A]AATTTAGAGTCTTAAACTTTATTGCTGTATTTTATTCCACGTATATATGGAATATAACGAAAAAGGCAGAAATAGAAAAAAAAAATAAGCGGATAGGCTTCTTTTTTGACAAGAGTATAATTGAGAGACTAAATACATAGATATAGTAAAGAACAATTTTGTTTTGAACAATAAATGTCTTTCACATCCAACTATAACAATAAACAACTTCTTTATTATGGCAGTTCCAAAAAAGCGCACTTCTAGATCAAAAAAGCGTATTCGAAAAAATATTTGGAAAAAAAAGGGATATTGGGCAGTATTGAAAGCTTTTTCATTAGCGAAATCTCTTTCTACAGGTAATTCAAAAAGTTTTTTTGTGCAACAAATAAAAAATAAAAGGTTGGAATAATCTGATTTGACTTGACACGAGAAAGGGTCTAATTTCGTTTATTGTTTATAATTTCTAATAATTTAGAATATAAATTTCTAATATAAAATAGAAATATATATAATATAGA |
| SNP78 | A/G | 120968 | CGGATAGGCTTCTTTTTTGACAAGAGTATAATTGAGAGACTAAATACATAGATATAGTAAAGAACAATTTTGTTTTGAACAATAAATGTCTTTCACATCCAACTATAACAATAAACAACTTCTTTATTATGGCAGTTCCAAAAAAGCGCACTTCTAGATCAAAAAAGCGTATTCGAAAAAATATTTGGAAAAAAAAGGGATATTGGGCAGTATTGAAAGCTTTTTCATTAGCGAAATCTCTTTCTACAGGTAATTCAAAAAGTTTTTTTGTGCAACAAATAAAAAATAAAAGGTTGGAATAATCTGATTTGACTTGACACGAGAAAGGGTCTAATTTCGTTTATTGTTTATAATTTCTAATAATTTAGAATATAAATTTCTAATATAAAATAGAAATATATATAATATAGAATAGAAAATATATAAAATATATAGAAAATATATAAAAAGGATTTCCATTCTTTTTTAATGTTTTGTTTTGAACTAATAAATATTAAATT[A/G]CACTCCTTTTGCTTTTTTCATATGTAGACTAAAGAATTCTTTTTTATACTGAATTCTAAAACTGGTACTTTTTGCAAAAAAAGAAAAAAAAAGAGAAGACACAAAGTTTCACCTTTCTTTTTATTGTTAGGATATTTTATCATTTTCGGGATGGGGATTATTATTTAAATTTTATTTTCCCCATCGACCCATTTGTCACAATCACAATACTAAACATTCATAAGTTTTTTCTTTTTTATACTATTTGCATTCTTTTTTATACTATTTGCATATAAAAGAAGAAATCTAAGACCTTTAGTCAAAAAAATTGACCGTTAAAATAATTGATTAAGTTTAAAATTTTTAACTTACTTAACAATTATTATATTTATATTATTCTAAATCGCTATATATAATAGATTCTTTGTTGCTTTCAATCTAATTAATAAAAAAAGCACCTTTTATCTTTAGATTTTCTCTTTAGAAAAAAATGTGTCAATTTTGAATGATCTACTTTATGC |
| SNP79 | C/A | 121030 | AACAATTTTGTTTTGAACAATAAATGTCTTTCACATCCAACTATAACAATAAACAACTTCTTTATTATGGCAGTTCCAAAAAAGCGCACTTCTAGATCAAAAAAGCGTATTCGAAAAAATATTTGGAAAAAAAAGGGATATTGGGCAGTATTGAAAGCTTTTTCATTAGCGAAATCTCTTTCTACAGGTAATTCAAAAAGTTTTTTTGTGCAACAAATAAAAAATAAAAGGTTGGAATAATCTGATTTGACTTGACACGAGAAAGGGTCTAATTTCGTTTATTGTTTATAATTTCTAATAATTTAGAATATAAATTTCTAATATAAAATAGAAATATATATAATATAGAATAGAAAATATATAAAATATATAGAAAATATATAAAAAGGATTTCCATTCTTTTTTAATGTTTTGTTTTGAACTAATAAATATTAAATTACACTCCTTTTGCTTTTTTCATATGTAGACTAAAGAATTCTTTTTTATACTGAATTCTAAAA[C/A]TGGTACTTTTTGCAAAAAAAGAAAAAAAAAGAGAAGACACAAAGTTTCACCTTTCTTTTTATTGTTAGGATATTTTATCATTTTCGGGATGGGGATTATTATTTAAATTTTATTTTCCCCATCGACCCATTTGTCACAATCACAATACTAAACATTCATAAGTTTTTTCTTTTTTATACTATTTGCATTCTTTTTTATACTATTTGCATATAAAAGAAGAAATCTAAGACCTTTAGTCAAAAAAATTGACCGTTAAAATAATTGATTAAGTTTAAAATTTTTAACTTACTTAACAATTATTATATTTATATTATTCTAAATCGCTATATATAATAGATTCTTTGTTGCTTTCAATCTAATTAATAAAAAAAGCACCTTTTATCTTTAGATTTTCTCTTTAGAAAAAAATGTGTCAATTTTGAATGATCTACTTTATGCTTGATGCTTGCGCTTGAAATTAGAATGAATCAAGGCATGTATTAAATTTGAAATTAGATATT |
| SNP80 | C/T | 121171 | TGGGCAGTATTGAAAGCTTTTTCATTAGCGAAATCTCTTTCTACAGGTAATTCAAAAAGTTTTTTTGTGCAACAAATAAAAAATAAAAGGTTGGAATAATCTGATTTGACTTGACACGAGAAAGGGTCTAATTTCGTTTATTGTTTATAATTTCTAATAATTTAGAATATAAATTTCTAATATAAAATAGAAATATATATAATATAGAATAGAAAATATATAAAATATATAGAAAATATATAAAAAGGATTTCCATTCTTTTTTAATGTTTTGTTTTGAACTAATAAATATTAAATTACACTCCTTTTGCTTTTTTCATATGTAGACTAAAGAATTCTTTTTTATACTGAATTCTAAAACTGGTACTTTTTGCAAAAAAAGAAAAAAAAAGAGAAGACACAAAGTTTCACCTTTCTTTTTATTGTTAGGATATTTTATCATTTTCGGGATGGGGATTATTATTTAAATTTTATTTTCCCCATCGACCCATTTGTCACAAT[C/T]ACAATACTAAACATTCATAAGTTTTTTCTTTTTTATACTATTTGCATTCTTTTTTATACTATTTGCATATAAAAGAAGAAATCTAAGACCTTTAGTCAAAAAAATTGACCGTTAAAATAATTGATTAAGTTTAAAATTTTTAACTTACTTAACAATTATTATATTTATATTATTCTAAATCGCTATATATAATAGATTCTTTGTTGCTTTCAATCTAATTAATAAAAAAAGCACCTTTTATCTTTAGATTTTCTCTTTAGAAAAAAATGTGTCAATTTTGAATGATCTACTTTATGCTTGATGCTTGCGCTTGAAATTAGAATGAATCAAGGCATGTATTAAATTTGAAATTAGATATTTGAAATTAGATAAGAGTTTTTTTTTTATTATTAACTGAATTTGGCTTCATTAATTTGAATGTTTCAAAAAAAAATATTGCATTGAATTGACTCCCTCAAGCTCGACGATTAAATAAAAATGGATTATTATGATTTCGAGTA |
| SNP81 | T/G | 121197 | AGCGAAATCTCTTTCTACAGGTAATTCAAAAAGTTTTTTTGTGCAACAAATAAAAAATAAAAGGTTGGAATAATCTGATTTGACTTGACACGAGAAAGGGTCTAATTTCGTTTATTGTTTATAATTTCTAATAATTTAGAATATAAATTTCTAATATAAAATAGAAATATATATAATATAGAATAGAAAATATATAAAATATATAGAAAATATATAAAAAGGATTTCCATTCTTTTTTAATGTTTTGTTTTGAACTAATAAATATTAAATTACACTCCTTTTGCTTTTTTCATATGTAGACTAAAGAATTCTTTTTTATACTGAATTCTAAAACTGGTACTTTTTGCAAAAAAAGAAAAAAAAAGAGAAGACACAAAGTTTCACCTTTCTTTTTATTGTTAGGATATTTTATCATTTTCGGGATGGGGATTATTATTTAAATTTTATTTTCCCCATCGACCCATTTGTCACAATCACAATACTAAACATTCATAAGTTTT[T/G]TCTTTTTTATACTATTTGCATTCTTTTTTATACTATTTGCATATAAAAGAAGAAATCTAAGACCTTTAGTCAAAAAAATTGACCGTTAAAATAATTGATTAAGTTTAAAATTTTTAACTTACTTAACAATTATTATATTTATATTATTCTAAATCGCTATATATAATAGATTCTTTGTTGCTTTCAATCTAATTAATAAAAAAAGCACCTTTTATCTTTAGATTTTCTCTTTAGAAAAAAATGTGTCAATTTTGAATGATCTACTTTATGCTTGATGCTTGCGCTTGAAATTAGAATGAATCAAGGCATGTATTAAATTTGAAATTAGATATTTGAAATTAGATAAGAGTTTTTTTTTTATTATTAACTGAATTTGGCTTCATTAATTTGAATGTTTCAAAAAAAAATATTGCATTGAATTGACTCCCTCAAGCTCGACGATTAAATAAAAATGGATTATTATGATTTCGAGTAAGCCGCTATGGTGAAATCGGTAGACA |
| SNP82 | G/T | 121587 | TTTTATTGTTAGGATATTTTATCATTTTCGGGATGGGGATTATTATTTAAATTTTATTTTCCCCATCGACCCATTTGTCACAATCACAATACTAAACATTCATAAGTTTTTTCTTTTTTATACTATTTGCATTCTTTTTTATACTATTTGCATATAAAAGAAGAAATCTAAGACCTTTAGTCAAAAAAATTGACCGTTAAAATAATTGATTAAGTTTAAAATTTTTAACTTACTTAACAATTATTATATTTATATTATTCTAAATCGCTATATATAATAGATTCTTTGTTGCTTTCAATCTAATTAATAAAAAAAGCACCTTTTATCTTTAGATTTTCTCTTTAGAAAAAAATGTGTCAATTTTGAATGATCTACTTTATGCTTGATGCTTGCGCTTGAAATTAGAATGAATCAAGGCATGTATTAAATTTGAAATTAGATATTTGAAATTAGATAAGAGTTTTTTTTTTATTATTAACTGAATTTGGCTTCATTAATTT[G/T]AATGTTTCAAAAAAAAATATTGCATTGAATTGACTCCCTCAAGCTCGACGATTAAATAAAAATGGATTATTATGATTTCGAGTAAGCCGCTATGGTGAAATCGGTAGACACGCTGCTCTTAGGAAGCAGTGCTAGAGCATCTCGGTTCGAGTCCGAGTGGCGGCATAACATCCTGTAATTATCAAAAGGATACAATAAATCCTATAATGAATTCAATTCCTGATTTCACCATAGCGGCTTACTTTTCTAATTAAAGAGCCTTTTTATTTTATTTATTATTATTAATTTATTAATTTAAAATTTTTTAAAAATTTTATGATATTTTCAACTTTAGAACATATATTAACTCATATATCTTTTTCAGTCGTGTCACTTGTAATTACAATTCATTTGATAACTTTAGTAGTCGATGAATTCGTTGAACTATATGATTCGTCAGAAAAGGGCATGATAACCACTTTTTTCTGTATAACAGGATTATTAGTTACTCGTTGGATTTC |
| SNP83 | T/A | 121893 | ATAAAAAAAGCACCTTTTATCTTTAGATTTTCTCTTTAGAAAAAAATGTGTCAATTTTGAATGATCTACTTTATGCTTGATGCTTGCGCTTGAAATTAGAATGAATCAAGGCATGTATTAAATTTGAAATTAGATATTTGAAATTAGATAAGAGTTTTTTTTTTATTATTAACTGAATTTGGCTTCATTAATTTGAATGTTTCAAAAAAAAATATTGCATTGAATTGACTCCCTCAAGCTCGACGATTAAATAAAAATGGATTATTATGATTTCGAGTAAGCCGCTATGGTGAAATCGGTAGACACGCTGCTCTTAGGAAGCAGTGCTAGAGCATCTCGGTTCGAGTCCGAGTGGCGGCATAACATCCTGTAATTATCAAAAGGATACAATAAATCCTATAATGAATTCAATTCCTGATTTCACCATAGCGGCTTACTTTTCTAATTAAAGAGCCTTTTTATTTTATTTATTATTATTAATTTATTAATTTAAAATTTTT[T/A]AAAAATTTTATGATATTTTCAACTTTAGAACATATATTAACTCATATATCTTTTTCAGTCGTGTCACTTGTAATTACAATTCATTTGATAACTTTAGTAGTCGATGAATTCGTTGAACTATATGATTCGTCAGAAAAGGGCATGATAACCACTTTTTTCTGTATAACAGGATTATTAGTTACTCGTTGGATTTCTGGGGGACATTTACCATTAAGCGATTTATATGAATCATTAATCTTTCTTTCATGGGCTTTTTCCATTATTCATATGGTTCCGTATTTTAAAAAACATAAAAATTATTTAAGCGCAATAACCGCCCCAAGTACTTTTTTTACCCAAGGGTTTGCTACTTCAGGCCTTTTAACTAACACGCATCAATCCAAAATCTTAGTGCCTGCTCTCCAATCCCAGTGGTTAATGATGCACGTAAGTATGATGATATTGGGCTATGCAGCTCTTTTGTGTGGATCATTATTATCAGTAGCATTTCTAGTAATCAC |
| SNP84 | A/C | 124810 | AAAATAATCCTATTTGACCACTATACATTGCTAACATCAGAAAATTAAATAAGCGGGAATCACGAGTAATTGGCCAAGCCGCTAAAGTAGCTAAAGTGGTGATAAATCCTGTCAGTAAAATAGGGCTTAAAGAAAATCCATCTATTCCCAATCTCCAGTAAAAATCAAAAAATTGGATCCATTTATAATCTTCTGTTAATTGGATTAATGGGTCGTCCAATTGGAAATAATAAGAGAACGCATAAGTCATTAAAAGGAGTTCTAAAATACATATAAACAAAGTATACCACCTAATTACCTTATTTCCTCTATGGGGGAAAAAGAAAATTAAGGAACCCGCAGATATCGGTAAAACTACAAATATTGTTAACCAAGGAAAAGAATTCGTGGTAAAGGCAAGATACATTTGGACTAGAAAAACCCGTACTCGAAAACATAATATATATATATTTTTTGTATTTTTTGTTTTTCGAGTACGGGTTTTTGTCGGTAAACATAAA[A/C]TCAAATGCATTCAAGTGGATTTTTCTGAAAAGTATCAATAAGCTAGACCCATGCTTCGGGTTGTTTCATGCCATAAATAAACTCGAACACTCAAGAAATCAGTTGGACAGGCGGATTCACATCTCTTACAACCAACACAGTCTTCTGTTCTTGGAGCAGAAGCGATTTGCTTAGATTTACACCCATCCCAAGGTATCATTTCTAATACATCTGTGGGGCAGGCTCGGACACATTGAGTACACCCTATACATGTATCATAAATCTTTACTGAATGCGACATTGGATTTCTAAATTTTTTAACGTCATAAAATTTCAATCTAGTTAATTTCTAAATGAATCATCATATATTTAGACACCAGACGAATCAATGATTTATCAGAATTTTTCTATTGAATTCTGGATCGACTCGTGAGATAGGGCCAGGATACTTTAATTTCGTACGTTTTCGCAAACATGATCGGATAAGTTACGTATCATACATGCCAACTTGAATTAATGAA |
| SNP85 | A/T | 126532 | TAATCTGATTTGTTTTTGTAGTCCAAATAATCCCGTACCATGACGTATCTGGAATAATAGTAATTAGTGAAACAAAAATACTTGTACAAATTAAGGAAGTAACTCCGGTTCCAACAGTCCAAAGATTAAAATCTTTGTAATATTCTGAACCATTCATGAACATCACGGCAAATATAATTAAAACGTTTATGGCTCCCACATAAATAAGGAGCTGCGCAGCAGCTACAAAATGAGAGTTTGATAAAATATAGAATAAAGATATACAAACAAGAACCAATCCCAATGAAAAGGCAGAATAAATTGGGTTGGTAAGTAATACCACTCCTAGACCTCCTAATATAAGACCTAATCCCATAAAAAATAAAAGAAAATCATGTATTAGTCCAGGCAAATCCATTGCACGTAAAATAAGAGATAAAAAAATTGAATTGTTTTTTCATGACCTTATTGACCTGACCAGGAAAAACTTATTTATAATAGGTTCTAAACCCTAAGGGGTG[A/T]AAATTACTATAGGTACAGCTATTGTATGAATCTCATTCTTTCTCGAAAAAGGCATTCTAAATTAAATTTTAGAAATAATTATGGCTAGAATTACGCATATATTAATAGATTTTCACAAAAAAATAAAGCCGCGATGCAATTCTGACCAATCAAGTCAATAGTTGGAATTTGTAATTTTTGTATTTATTGACCAAGAAAAATAAAAAAAAAACCTATTGCAGCCCTTTAGATCAAAACAGAATTTTAGTTTAATAATTGTACTTTTTAATCAATCAAAGCGGGTTATCTTTTTTGTTTCTAGTTGAATTCAAAATTGTTCGAATTGTATAATCGTCGACTACTGACATTGGTAAACGACCTAAAGCAATTTGATTATAATTCAATTCGTGACGATCATAAGTGGAAAGTTCATATTCTTCAGTCATTGATAAACAATTTGTTGGACAATACTCAACACAGTTGCCACAAAATATACAGATTCCGAAATCAATACTGTAATT |
| SNP86 | G/T | 126814 | ATGAAAAGGCAGAATAAATTGGGTTGGTAAGTAATACCACTCCTAGACCTCCTAATATAAGACCTAATCCCATAAAAAATAAAAGAAAATCATGTATTAGTCCAGGCAAATCCATTGCACGTAAAATAAGAGATAAAAAAATTGAATTGTTTTTTCATGACCTTATTGACCTGACCAGGAAAAACTTATTTATAATAGGTTCTAAACCCTAAGGGGTGAAAATTACTATAGGTACAGCTATTGTATGAATCTCATTCTTTCTCGAAAAAGGCATTCTAAATTAAATTTTAGAAATAATTATGGCTAGAATTACGCATATATTAATAGATTTTCACAAAAAAATAAAGCCGCGATGCAATTCTGACCAATCAAGTCAATAGTTGGAATTTGTAATTTTTGTATTTATTGACCAAGAAAAATAAAAAAAAAACCTATTGCAGCCCTTTAGATCAAAACAGAATTTTAGTTTAATAATTGTACTTTTTAATCAATCAAAGCGG[G/T]TTATCTTTTTTGTTTCTAGTTGAATTCAAAATTGTTCGAATTGTATAATCGTCGACTACTGACATTGGTAAACGACCTAAAGCAATTTGATTATAATTCAATTCGTGACGATCATAAGTGGAAAGTTCATATTCTTCAGTCATTGATAAACAATTTGTTGGACAATACTCAACACAGTTGCCACAAAATATACAGATTCCGAAATCAATACTGTAATTAAGCAACCGTTTCTTTCGAATGTCAGTTTCCAATTTCCAATCAACAACAGGTAGATCTATAGGACATACCCGAACACATACTTCACAAGCAATGCATTTATCAAATTCAAAATGGATTCGACCGCGGAAACGCTCCGATGTGATTAATTTTTCATAAGGATATTGAATAGTTACAGGTAAACGATTTGCATGGGATAAGGTAATCATGAAACTTTGACCAATATACCGTGCAACTCGTATGGTTTGTTGCCCATAATTCATGAACCCAGTTACCATGGGAAA |
| SNP87 | T/G | 132497 | TTCAATAGAAATAGAATCGATTTTATCTCTGTATTGTTGATTTATTTGTTGTTTGTTCTTATGAAATAATAAGATACTTATGGTTTGATACAAAAGAAATTGTCCATTATTTTTTACCGATAGACGAATAGGTTCGATAGTAAATATTCTCTTTTTCATCAATTCTGTAAGAGTTAAATCCTTCTGAATCATCAAAATATTCAGACTTATTTCTTGCCTTTGAATAGAAGATATAATAATTTCGCGTGGATTTGTCAGTTTAAGCAGGAGACAATATACTTTGATATTATTGATTATTTTTTGATTTAAAGAACCATTCCATCTTAATTGAAAATATAAATATCGTTTTAGAAAGAAATCAAGTTCTACTTCCGTATGACTTTTGTTTTTCTTTTTATGTCTATGTTTTTTCATATCTAATTCCATATAATCTTTTTCAATATCTTTTTTTTGATTGGATGAATTGACTTCTTGATCTTGGTGAATTGTAATAAAAAAAA[T/G]CTTTTTTTTATCCATTTTCTCAATTATTTGATACTTAGTAGTCTTAGTATTTTTTTTATGTTCGCTTCCGGTATCGATCCAGGATTCAATCTCGACCTTCTCTCTAAGACAAAAATTGAGAATTTTCCAATCAAAATATTTTCTATCTGGGCTTTTCGCCATATCGATAATATCATCTTCCGCTAGATAATTATTGATAGGAATACCTTCTAACATGTCGAAAAAGTTACTTTTATTTGTGTTGTAATTATAAGAAATCTTTTGTTTATTATTTATTTGTAATGGTGATCCATAAATATATGAGTCCTTCTTATTTTCATAATTAATAGATTTATATGATAAAAGATTATATTTATAGTGTTTTTTAAAATTATCTTTTTGATTCGGTAATGAGTCTGCCTCAAAATCCTTTTTTTTTTCGTAATGAATTAATTTGTCTTTTTCATATAAATTCCATTTGTTTAATTTTTTGTTTTGAACCATAGGGTGTTGGTATTGAT |
| SNP88 | C/A | 132498 | TCAATAGAAATAGAATCGATTTTATCTCTGTATTGTTGATTTATTTGTTGTTTGTTCTTATGAAATAATAAGATACTTATGGTTTGATACAAAAGAAATTGTCCATTATTTTTTACCGATAGACGAATAGGTTCGATAGTAAATATTCTCTTTTTCATCAATTCTGTAAGAGTTAAATCCTTCTGAATCATCAAAATATTCAGACTTATTTCTTGCCTTTGAATAGAAGATATAATAATTTCGCGTGGATTTGTCAGTTTAAGCAGGAGACAATATACTTTGATATTATTGATTATTTTTTGATTTAAAGAACCATTCCATCTTAATTGAAAATATAAATATCGTTTTAGAAAGAAATCAAGTTCTACTTCCGTATGACTTTTGTTTTTCTTTTTATGTCTATGTTTTTTCATATCTAATTCCATATAATCTTTTTCAATATCTTTTTTTTGATTGGATGAATTGACTTCTTGATCTTGGTGAATTGTAATAAAAAAAAT[C/A]TTTTTTTTATCCATTTTCTCAATTATTTGATACTTAGTAGTCTTAGTATTTTTTTTATGTTCGCTTCCGGTATCGATCCAGGATTCAATCTCGACCTTCTCTCTAAGACAAAAATTGAGAATTTTCCAATCAAAATATTTTCTATCTGGGCTTTTCGCCATATCGATAATATCATCTTCCGCTAGATAATTATTGATAGGAATACCTTCTAACATGTCGAAAAAGTTACTTTTATTTGTGTTGTAATTATAAGAAATCTTTTGTTTATTATTTATTTGTAATGGTGATCCATAAATATATGAGTCCTTCTTATTTTCATAATTAATAGATTTATATGATAAAAGATTATATTTATAGTGTTTTTTAAAATTATCTTTTTGATTCGGTAATGAGTCTGCCTCAAAATCCTTTTTTTTTTCGTAATGAATTAATTTGTCTTTTTCATATAAATTCCATTTGTTTAATTTTTTGTTTTGAACCATAGGGTGTTGGTATTGATT |
| SNP89 | A/C | 134080 | TTCTTCTTTTTAATATCTGTGAAATATTTTTTGATGATTTTAATTTTAATGTTTTAGCATTATAACTTATTTTGTTAGGACTAATATTTATCTCCGAGGTTATAATTTTTTTTTCCTTTTCTTTTGAAATTTTTTCTATTTGATTTAAGATTATCTTTCGTCTAGCAGAAAGATCTTTCATTTTTTTTTCTGTCAGTGAAAAATTTGTCCAAACCATAGATCGCGTTGAGGTGGACATTTTCTGACTCGCCCGATTATTCTTATTGATTAGCGAATCTTTTTCTTTTTTAGTTGCATTCAATTCATATACTTCTTTAACTCCAGATAATAGAATTCGGTTTCTTTTTGATTTTTCAAATTTGTTTATTTTGTTTATTATGTCTTTTCGAAAAAAAGAGTTTTGGATGACCCAGTTTGCTTTTTCTTTTGATAAATTTAGAAATAATTTTCTTCTTTCTTCTAAAGTTCTTCTAATTCGAAAACCCTTTTTTTTCATTTTT[A/C]TAATTTTTTTTTCAAGTTTTTTAAAGATGGGTTCAAAAAGTGAAAGCCGTTTTCGGGGAAAACCAAAAGGCAGTTCAGCCTCCATCCCCAAAACTGTTAAAAAACAAAAATCATTTCTTTGTACTTTCTTTTTCATTGGATCTTTATGAGGGAATTTTAACTTAGATCTGTGCCAAGGTTTTAGACGAAAAGGAAATAGGATCTTTATTTGAATCCCGTCTGTTAACCAGTTTTTCGGAAATTCTTTTTCTGATAATTGAACTCCATTATAAGTGCATTTAATATGCATTTCTCTACTCCAATCCTTTAAATCCTCCGACCATTCGGGAATTTGAAAAAATAGTATACGAATGCAATTTTTAGCTATTATTAATAAAGGTAATATAATATATTTTCTAAGAATCGATTGGGTTACTAAAATACAACCTCTTATTATTTGAGCAAAAAGAATGCTATCCCAGGCTTCAGCTATTTCTATCCGAGTTCTTTCCTCTCTTTTG |
| SNP90 | A/C | 134427 | GATTTTTCAAATTTGTTTATTTTGTTTATTATGTCTTTTCGAAAAAAAGAGTTTTGGATGACCCAGTTTGCTTTTTCTTTTGATAAATTTAGAAATAATTTTCTTCTTTCTTCTAAAGTTCTTCTAATTCGAAAACCCTTTTTTTTCATTTTTATAATTTTTTTTTCAAGTTTTTTAAAGATGGGTTCAAAAAGTGAAAGCCGTTTTCGGGGAAAACCAAAAGGCAGTTCAGCCTCCATCCCCAAAACTGTTAAAAAACAAAAATCATTTCTTTGTACTTTCTTTTTCATTGGATCTTTATGAGGGAATTTTAACTTAGATCTGTGCCAAGGTTTTAGACGAAAAGGAAATAGGATCTTTATTTGAATCCCGTCTGTTAACCAGTTTTTCGGAAATTCTTTTTCTGATAATTGAACTCCATTATAAGTGCATTTAATATGCATTTCTCTACTCCAATCCTTTAAATCCTCCGACCATTCGGGAATTTGAAAAAATAGTAT[A/C]CGAATGCAATTTTTAGCTATTATTAATAAAGGTAATATAATATATTTTCTAAGAATCGATTGGGTTACTAAAATACAACCTCTTATTATTTGAGCAAAAAGAATGCTATCCCAGGCTTCAGCTATTTCTATCCGAGTTCTTTCCTCTCTTTTGTCGTCTTCTCTTGTGTCCTCGTCTTTTTTCTTACTTTCTTTTGTTTTTTTCTCTGTATAAGTATAATCTGAAATTGTGAATTCTGCGTTTTTACACATCCAATTTATAAACATTGTTTTTATCAGTTCGAAAATATCAAAAGAAAAAAAAAGAGATTTGTCTATTCTGTCCAAAAAAAGGGGCGAATGCGCATTTGCTTGAAACAGTTCAAAAATAGCTATTTTGCGTCTTTGTGCTCGCGTGGATCCTTTTATTATGTCTCGACGAAAGTCTGATTGTTGCGAATAACGGATCAAAGTAACTTCGTCTATTTGGTCAAAATCTGTCGTATCTTTGGTATTATTATA |
| SNP91 | T/G | 8795 | TTATAGCAATTGATAAGATCAGATAAGTCTTATAGTATAAAGTATAAACTCTTAATTCAAATATTGAAGTTATTGTATAAATATAAACTATAAACGCGAGAATTCTGGATCACCCCATTTTTTTTCATTCTAAACTTTTTTTCCATTGCAGATCCTATTAGAATCCTTTTTGTAGTTATGAAAAAATATCTAATTTTCGGTATGAAACAAAATTTTTGGCAATTAAAGACTCGACTTTTATTACAAATGAATTTTGAATTTAGAAAAATTATTTTCTATTTCTAGAAACCGCTCCATGTCTTGGTGTTAAAATAGAATATGTGGTATAAAAATAGAGAATCTATTTTTTTTTCCAAACCAAAAAAAGATCTTGGAGATTTTATAATGCTTACTCTCAAACTCTTTGTTTACACAGTAGTGATATTCTTTGTTTCTCTATTCATCTTTGGATTTTTATCTAATGATCCAGGACGTAATCCCGGACGTGAAGAATAAAAAAA[T/G]AAAATATATAAAATTAAAATAAAAAAAAAATATAAGGGTTTTTCTTGATTTTTCAATGTTCTTAGTATTTTACTATTCTACATTTTTAAATATTAAATAAAACAAAAAAGTTCGCTAAATTTAAAAGAAAAGAAAAAAATTCCAAGTCATCACCGGAACCGGAAAGAGAGGGATTCGAACCCTCGGTACGGATAACTCGTACAACGGATTAGCAATCCGACGCTTTAGTCCACTCAGCCATCTCTCCCGATTGAAAAAGGATACTTACTATGTTACATTACACAACAGGTAAGGCTTGAAAATAAAAAAAAAGCCTTTTTCCCTCTTTTCTTTATTTTATTACTTTTATTACTTTCAAAATTACTTTTTATATTTTTTTTATTCTTTTATTTTTATATGGAACTATTTTATTTAATTTAATATATTAATTTAATATATTAAATTAAATATATATTCTATAATTATATATTTAATTTGAATAATTATATATTCAATTTAAA |
| SNP92 | A/C | 24512 | CTATAGGTCTCCCCCCTTCAAAATCGGACGTGAAAGTTTCCTCTCATCCGGCTCAAGTAGTTATACCAAATAGAAAGAAAAGGGTTCCTGCTTTCAAATTTCAAAATCGATAAAAACCAAAAAAAGAGCTACTCCTTACTCAAGTTCCCAATGAATAATGAACACCAAGCAAGCTCTCATTAATTCCTTCTTCCTTTTTTTTTTTCGAATTTCTGAATTCTTTATTCAATTATCACAACTTATTCAATTTTCACAATTACGATCTAAATGAAATGTGAAATTCTTGAGTAGTCTACTTCCCTTCGAATGATAAAATCCTAGTACCTTAGAATTCATAAGGGATTTATTTGTCTATGTCTCTTTCTATTCGATCTCTTAGGTCCCGAACTCACCTCGACGGTTATGCCACGATGCTGTTAAAGCCTATATGCGATGTATAGACTCCTGTAACCATGACATATTTGATTACTTGAGTATAAACATAGTTTCTTTCTATTTTT[A/C]AAATAAAAAGAAAGAAAGAAGTCGGTTTTCACGAGGTACAACTAGGAATTCCTATTTTTTCGTTACTGCATCGACCATAGACCAATTCCTCTTTTATTGGGGAGTATTGAATACACCCATAATTCTGAGCTTCATGTTACTCCTCTCAAGAGACATGTCAGATCCAGGGCATCCCAAAATAAATGGGATGACAGTTTTTCATTTCAAAACTGTAAAATCAGAATTTCGATCAAATCACACATCGCAATATACTAGGCCTTCTAATTCTTTAAGAGGTTTATCTAAAAGATTTGCGATATAACTAGGAAGACGTTTCAAATACCACACATGAGTTACTGGGCATGCCAGTTTGATGTAGCCCATTTGATATCTTCGTATCCGAGAATCAACAAATTCGACTCCGCATTGTTCACAAAATTTTTGGTCTTCTTTTTCATTTCTGATTACTCGATAATTTCCACAAGCACAAATTCCACTTTTTATAGGCCCAAAAATTCTTT |
| SNP93 | A/G | 63073 | GCTTATTCGATTCAATCGTACCACGTAATCCGTTAAAAGGCGTTCTGAATGAGTTACTTCAGCTCCACGATTTCTTTCCTTTGAATCATAAATCAAGTAGAGTCTGAAGTTAAATTAATTATTATGAAGTTAAATTAATTATTAATTAAATTTTAAAAATTTTAAATTAAATTACTTAATTAAAATTAAAAATAAAAATGAGTTAATTAAATTAAAATTAGTTAATTCATTTTTATTTCTGGCGAGCAGGTATTTTTTTTAGCAGAATCAAAGGAAAAATCAATTTTTTTGATTTTTTTGTGACATAACATAAGAGTAAATTGTAGAAAGAATCATGTAGATAATTTTTTTTTACCGATATTTCTGATTACTAATTAAGAAACCTCTATCAACAAGATCAACAAGAAAAAGAGTGAATTCTTTTTTTCGTGAAAAAATGGGCAAGGTATAATAAATAAAACGAATTTCATGTTCTTTTCTTACCTATGCATAGAAAATAT[A/G]GAGTCTTGCATATTTATATATCTCCTGGGAATCCCTTTTTTTTTACTAAAAAAAAATCGACGGATTCTAAATTATAACGAATTTTGGGGGAATTTATAATCGGAAAAACTCTTTATTTTTTTTTTTATTTTATATTAAATATATTAAAATATTTTCTATTTTCTATTAAAGTAAAGTTAAAAAAATGAAAAAAGAAAAATGAAAGTTATAAGACAAGAAAAAAAAAGATAATAAAAGAAGAATAACAAATAAATGGATTATCATATTTCATGTAGAAATATGAATAAGTCATTTAGTTAGTTCTACGCTCTTTGCACTTTATTATATTATATATACTCACTTAGATATACTTAGTATAATTATATACGAATTTTAATGATTATAAGAAATCTTTCTAATAACTAAATAACTAATAAATAACTAATAATAGAATAATTAATATAAATATAATTATAATTAATTTAAATTATTAATTTAAATTATTAAATTAATAGAATAAA |
| SNP94 | G/T | 86159 | ATGATATTAGAAACCTCTTCTTTCTCTTTTTTTTTTTTTCACAAATAAGAAGTTGGGATACAATTCGGATATCCGAAAGATTACCATATATAACACAAGATTTCTCCACCGATTCTTTCTAGTCGAGCTTCTCGGTCGGTCATTATACCCCGAGAAGTAGAAAGAATTACAATACCCATCCCGCCCAAAATTCTAGGAATTTTTTGATAGTTAGAATAGATTCGTAGACCAGGTCGGCTAATCCGTTTTAAATTTAGACTAGTTCTATAGGGTCCCTTCCTCTTCCTTCTATGTCGTAGGGTTAAAACCAAAAATTTTTTGTTGCCTTCCTGGTGTTTCCTGACATTTTCAATAAAACCTTCTCGTAAAAGTATTTTAATAATGTTTTCAGTGATGTTAGTGAATGCTATTCGAACGGTTCCTTTTCTATTCATGTCAGCATTTCGTATAGAGGTTATTATCTCAGCAATAGGATCCCTGCCCATGATAAACTAAATTTT[G/T]AATTTCTCTCTAGTTTTGATATAATCAACATACTTTTTTTTTTTCTTTTTTTTTTTTTGAATTACTTATTTTATTAAAGGTATATGCGTGAAACACAATTTACTAATGAAATTCTATTTCATTTTTATTCAAATACTATAACTATACAATAAAAACTATATAAAAAATACTATAACTATATACTAGAACTGTATCATGGTCTCATCTTAATTTTAAATGGTATTATCTTCTATTTTATTTAGAATTTTTTATTTTATAATACTTCAGGCGCTAATGAAACTATTTTAGTAAAATTTAACTGTCTCAATTCTCGGGCGATTGCACCAAAAATTCGAGTTCCCTTTGGATTTCCTTCTTGATCAATGACAACTGCAGCATTGTCATCATATCGTATTATCATACCGTTATCACGTTTGAGTTCTTTACAAGTACGTACAATTACAGCTCTGATTACTTCTGATCTTTCTAGAGGTGAATTTGGTGCCGCTTCCTTGATCACA |
| SNP95 | G/T | 126139 | GCATCATTGACCAACTCCTTATCAATTTCGATTTATTTCAATATGAACAACAATTCAACATCAACAGATTGGATTGACTAGAATAAGAATATCCAAAGTACAGAACAAAGAAATATATGGATAATAGATCAAATATGGATAATAGAATAATAGATCAAATAAAAGAATTTATACATAAATAATCTTTAAATGGAATTGAAAGAAAATAAATGTGATAACATAGAACAAATAGATTGGGGTTACTAATTCTAAAGATTTATTGCTGACGAGCCGCAGCAATCGCACCTATCAAAGCAACTAAAAGAATTATTGAAATGAATTCGAACGGAAGAAAAAAATCTGTTGATAAATGAATTCCAATTTGTTGACCATTACTTATTAAATCTTGTTCTATAATCTGATTTGTTTTTGTAGTCCAAATAATCCCGTACCATGACGTATCTGGAATAATAGTAATTAGTGAAACAAAAATACTTGTACAAATTAAGGAAGTAACTCCG[G/T]TTCCAACAGTCCAAAGATTAAAATCTTTGTAATATTCTGAACCATTCATGAACATCACGGCAAATATAATTAAAACGTTTATGGCTCCCACATAAATAAGGAGCTGCGCAGCAGCTACAAAATGAGAGTTTGATAAAATATAGAATAAAGATATACAAACAAGAACCAATCCCAATGAAAAGGCAGAATAAATTGGGTTGGTAAGTAATACCACTCCTAGACCTCCTAATATAAGACCTAATCCCATAAAAAATAAAAGAAAATCATGTATTAGTCCAGGCAAATCCATTGCACGTAAAATAAGAGATAAAAAAATTGAATTGTTTTTTCATGACCTTATTGACCTGACCAGGAAAAACTTATTTATAATAGGTTCTAAACCCTAAGGGGTGAAAATTACTATAGGTACAGCTATTGTATGAATCTCATTCTTTCTCGAAAAAGGCATTCTAAATTAAATTTTAGAAATAATTATGGCTAGAATTACGCATATATTAATA |
| SNP96 | A/C | 121304 | TCGTTTATTGTTTATAATTTCTAATAATTTAGAATATAAATTTCTAATATAAAATAGAAATATATATAATATAGAATAGAAAATATATAAAATATATAGAAAATATATAAAAAGGATTTCCATTCTTTTTTAATGTTTTGTTTTGAACTAATAAATATTAAATTACACTCCTTTTGCTTTTTTCATATGTAGACTAAAGAATTCTTTTTTATACTGAATTCTAAAACTGGTACTTTTTGCAAAAAAAGAAAAAAAAAGAGAAGACACAAAGTTTCACCTTTCTTTTTATTGTTAGGATATTTTATCATTTTCGGGATGGGGATTATTATTTAAATTTTATTTTCCCCATCGACCCATTTGTCACAATCACAATACTAAACATTCATAAGTTTTTTCTTTTTTATACTATTTGCATTCTTTTTTATACTATTTGCATATAAAAGAAGAAATCTAAGACCTTTAGTCAAAAAAATTGACCGTTAAAATAATTGATTAAGTTT[A/C]AAATTTTTAACTTACTTAACAATTATTATATTTATATTATTCTAAATCGCTATATATAATAGATTCTTTGTTGCTTTCAATCTAATTAATAAAAAAAGCACCTTTTATCTTTAGATTTTCTCTTTAGAAAAAAATGTGTCAATTTTGAATGATCTACTTTATGCTTGATGCTTGCGCTTGAAATTAGAATGAATCAAGGCATGTATTAAATTTGAAATTAGATATTTGAAATTAGATAAGAGTTTTTTTTTTATTATTAACTGAATTTGGCTTCATTAATTTGAATGTTTCAAAAAAAAATATTGCATTGAATTGACTCCCTCAAGCTCGACGATTAAATAAAAATGGATTATTATGATTTCGAGTAAGCCGCTATGGTGAAATCGGTAGACACGCTGCTCTTAGGAAGCAGTGCTAGAGCATCTCGGTTCGAGTCCGAGTGGCGGCATAACATCCTGTAATTATCAAAAGGATACAATAAATCCTATAATGAATTCAAT |
| SNP97 | T/C | 71077 | CACTCTATTTTTTGAGTGCTGTCTATAATCTATAATGATAATGATTGATAAATGATTGATAAATAAAAATAAATAAAAAGTGCTCAATTGAACTTTTTCTTTCAATTGGTATTTTTGTTTATCTTCGTATCTTTTATCTTTCAAAAAATCGAACTTAGGAAAGTACTTTTGCTTTACAAGTATATGTATAAAAAGGTTTATTTAGTTTCTATATGCTTACTATAACTAGTTATTTCGGTTTTCTACTAGCGGCTTTAACTATAACCTCAGTTCTATTTATTGGTCTGAGCAAGATACGACTTATTTGAAATAAATTGAATGAACAATTTATAAAAAAATTTATAAAAAGAAAAGGAAATTTTTCGGCAGTATTCCATCTATTCTCTAGTTCTTTACCGTGTTAATTTTCAATTCTTGCTTATTGAGATTTTTCTTATTGAGATTTATGGGCGATATGGATTAATATTTAAGGATAGATATTACCTTTCTTTCCCCCCCCC[T/C]TTTTTTTTTTCAAATAAATTGAAATGATTGAAGTTTTTCTATTTGGAATCGTCTTAGGTCTAATTCCTATTACTTTGGCCGGATTATTCATAACTGCATATTTACAATACAGGCGTGGTGATCAGTTGGACCTTTAATTAATTAATTAATTAACACCTTGTTTTTTTGACCTCCTCTTTTCTTTAATCCACAGGAGGTCAAATTCAGATTGCTCTTCCATTTTTTCTGTATAAAATTTGACCTAACAAAACAAGAACAGAATCACGCTCTGTAGGATTTGAACCTACGACATTGGGTTTTGGAGACCCACGTTCTACCGAACTGAACTAAGAGCGCTTTCTTATCCCAATCACAAAAAAATCAGACTGTAAGTAAAAAGATTCTTTTTTTAACTACTCCAATATATCTTGAATGCCTATACTATCATATATAATATGATAAAAATAAAAGATTAAGTATGGCCAAGTTTAATTGATCTCAATTGATCCCTCGTTATTACT |
| SNP98 | C/T | 34123 | TCATTGTTAAGATAAATAGGCATATCTCTCTCTCACACTAAACCAGTAATTTAACAAATGAGAAATCTGGGAAGAGGGATAGGCATCAACAAGTTATCCAATTTGATTTTAAGAATTTGGTTGAGGGGACAAATAGAATCTTTTCATCAGCGATTCGATGAAATATCTTGGATCTATGTTGAATTGCTAAATACATGTATCAATCAAGCGAATTTCGGTGGTGAGGTTCGGCCTTGATAAATTCATTACATTGATATTTATATTTATCAAATTCAATATCAATAAACTCTTTTTTTACCTATATTCACTAGTTTAGTCTAAACTCACTAGGTAAATCAAACCTTTCGCTTATAAATGAACTACTATGAATCTACTTCCTATACTTAGTATGTATAATATACTTATTCTAATTTATATTAGTTTCTATTAGAATTTATGAATTTAGAATAGAATTCTAAATTAGAATAATAATATTCCATGTTTAGAATAATCTAATTTTT[C/T]TAATATCTAATAATAGTATTAAAAAATAATGTTATTATTTTATATTTTAGATTAAAATATTTATTTATTTTATTTATTTAATATTTATTTATTTAAATAATATAAATAATATTTATTTATTTAAATAAATATTTATTTATTATAATATTTATTATAATTTATTATATATATTATATATTTATTATTCTAATTATATATATTTATTATTATAATTTTATTTTTATTATTATAATTTATTATATATTATATATATATATTTATTATTATATTATATTAATATTATATTTATTTATATTATATTTATTTATATTATATTTATAATTTATTTATATTATATTATATTTATTTATATTATATTTTATTTTTATTATAATATATAATAATTATAATATTTATTTAATCTTTATTATAATTTAATATTAATAATTTATTATATATAAATATATAATATTTATTAGAATATTATATATATTCTAATTTTATATATTAGATTAGAATTA |
| SNP99 | T/A | 34122 | CTCATTGTTAAGATAAATAGGCATATCTCTCTCTCACACTAAACCAGTAATTTAACAAATGAGAAATCTGGGAAGAGGGATAGGCATCAACAAGTTATCCAATTTGATTTTAAGAATTTGGTTGAGGGGACAAATAGAATCTTTTCATCAGCGATTCGATGAAATATCTTGGATCTATGTTGAATTGCTAAATACATGTATCAATCAAGCGAATTTCGGTGGTGAGGTTCGGCCTTGATAAATTCATTACATTGATATTTATATTTATCAAATTCAATATCAATAAACTCTTTTTTTACCTATATTCACTAGTTTAGTCTAAACTCACTAGGTAAATCAAACCTTTCGCTTATAAATGAACTACTATGAATCTACTTCCTATACTTAGTATGTATAATATACTTATTCTAATTTATATTAGTTTCTATTAGAATTTATGAATTTAGAATAGAATTCTAAATTAGAATAATAATATTCCATGTTTAGAATAATCTAATTTT[T/A]CTAATATCTAATAATAGTATTAAAAAATAATGTTATTATTTTATATTTTAGATTAAAATATTTATTTATTTTATTTATTTAATATTTATTTATTTAAATAATATAAATAATATTTATTTATTTAAATAAATATTTATTTATTATAATATTTATTATAATTTATTATATATATTATATATTTATTATTCTAATTATATATATTTATTATTATAATTTTATTTTTATTATTATAATTTATTATATATTATATATATATATTTATTATTATATTATATTAATATTATATTTATTTATATTATATTTATTTATATTATATTTATAATTTATTTATATTATATTATATTTATTTATATTATATTTTATTTTTATTATAATATATAATAATTATAATATTTATTTAATCTTTATTATAATTTAATATTAATAATTTATTATATATAAATATATAATATTTATTAGAATATTATATATATTCTAATTTTATATATTAGATTAGAATT |
| SNP100 | G/T | 31210 | TTTCTTGCTTAGTGCTTAGTTTAGTATTTGTTTTATTATTTTAGAGAAATTGGATTTGTGCTATACCTTATTGATTTGACTCATTTCATATTATGATTCAAAGGTTCAAAATCGGCAGGGTTTACTAATCCTTTTCGTTGAAATCTGAAAAAGTTTTTTTATAAAGTTTTTTTATAGAATTCCTTTTGATTGATTTTATTGTTTCGGTACATGGTAGGATTCATATTTTAAATAATCAGAAATCTAGGAATTAGAATCCTAAAAGTAAGAGTAAGAGTTGCCTTTCGACATTAATTGGAATCAACACAAATCAAATAGATGAAGAAATAGAATTGGGACGTTATGGACATTCCATATAGATAATTGATATCTAGACATATGAATATGAGATGATATTATAGATATAGATTAATTTATATCTAGATTAAGCCTATATCTTTATATAGTACAACTGTATACACTAGAATAAAAAAAAACGGATAGTATGATAGAAAGAAATA[G/T]ATTTCTTTCTATCATACTATCGGATCTCATAGAATACTGCTAATTCTAGTCTGCTCATTTCATTTAAGACTCAAAAAAGGAATCCTTTTCATTTTTTTTCTTCAATCATTGATAAGAACTAAGAAGTCAAAGAAGTCAAGTTTCATTCAAATTAATCACTTTGACTAACCGTTTTTACGTAAATTATAAGTAAAAAAGCAGTAGGAACTAGAATGAACAGGGCAGTAGCAATAAACGCAAGAATATTTACTTCCATAATCTCATCGTTTCTTTTTCTTTACTTCGTAATAACTCGGGATTTAATCCCATAGAGATAATAAATAAAAAATCTTTCGCCTCTAAATTCAATGGGATGAATTCCATCTCGATGATATCGAGTCTGATCAATATCATGAATAACAATATCTGAGTTATCAAATCGATTCATCGTCGAGAATTGAATAGTATAACATAGAAAGATCGTTTATCCATACTGAATTCAAAAATGGCTGGATTCTTGA |
| SNP101 | A/C | 31209 | TTTTCTTGCTTAGTGCTTAGTTTAGTATTTGTTTTATTATTTTAGAGAAATTGGATTTGTGCTATACCTTATTGATTTGACTCATTTCATATTATGATTCAAAGGTTCAAAATCGGCAGGGTTTACTAATCCTTTTCGTTGAAATCTGAAAAAGTTTTTTTATAAAGTTTTTTTATAGAATTCCTTTTGATTGATTTTATTGTTTCGGTACATGGTAGGATTCATATTTTAAATAATCAGAAATCTAGGAATTAGAATCCTAAAAGTAAGAGTAAGAGTTGCCTTTCGACATTAATTGGAATCAACACAAATCAAATAGATGAAGAAATAGAATTGGGACGTTATGGACATTCCATATAGATAATTGATATCTAGACATATGAATATGAGATGATATTATAGATATAGATTAATTTATATCTAGATTAAGCCTATATCTTTATATAGTACAACTGTATACACTAGAATAAAAAAAAACGGATAGTATGATAGAAAGAAAT[A/C]GATTTCTTTCTATCATACTATCGGATCTCATAGAATACTGCTAATTCTAGTCTGCTCATTTCATTTAAGACTCAAAAAAGGAATCCTTTTCATTTTTTTTCTTCAATCATTGATAAGAACTAAGAAGTCAAAGAAGTCAAGTTTCATTCAAATTAATCACTTTGACTAACCGTTTTTACGTAAATTATAAGTAAAAAAGCAGTAGGAACTAGAATGAACAGGGCAGTAGCAATAAACGCAAGAATATTTACTTCCATAATCTCATCGTTTCTTTTTCTTTACTTCGTAATAACTCGGGATTTAATCCCATAGAGATAATAAATAAAAAATCTTTCGCCTCTAAATTCAATGGGATGAATTCCATCTCGATGATATCGAGTCTGATCAATATCATGAATAACAATATCTGAGTTATCAAATCGATTCATCGTCGAGAATTGAATAGTATAACATAGAAAGATCGTTTATCCATACTGAATTCAAAAATGGCTGGATTCTTG |
| SNP102 | C/A | 27775 | TGGATTAGTTCGATCCAAAACTTGAGATAATGGGTGTAACCCGAAAAAAGATTCATAAGTGGTTGTTAATGGAGTTGAAGTTACCAAAGTTTGAGGAGTCGGTATCAATTTATGCCTAATTGCTCCACATATAGTCCCTCTAACCACATTTTCTAAACGAATCAGAGCCAATCCGAATTGATCTTGTAAAAGATCCGCTACAGAACGAATACGTTTATTTTTTAAATGATTCATATCGTCAAGTGTACCCATTCCAAATTTCATTCCAATCAAATGATCCGCAGCTGCCAATATATCTCGCGGTAACAAAAATGTATTGTTATGAGGTATATCAAGATTCAGTCTCCGGTTCATATTTAATCGACCAATCCTTCCTAATTCACATCTTTGTTGAAAGAATTTCTTTTGTAATTCCTTACATAAGGATTCAGAAAATACCGGATCTCCGCCTACACAAGTAAATTGTTGATAAAACTCCAAAATGGCATTTTCCTTTGACC[C/A]AATTTTTTTTTTTTCCTTATCATTCAGGAAAGATAAGAAAATTTCAGGGTAGCGCACATTCTCTAAAATTTCTCTTAGATTCAAACCCATAGCGGATAATAGAACTAGAATAGATATTTTCTGTTTCCTACTTACACGAGCCCATATCCTTGCTTTTCTATCAATCTCTAATTCTACTCGCCCCCCCCAATCTGATATTATGGTGCCGGTATAGACCGAAATTCCGTTATGATCCAATTCTGACCGGTAATAGATACCGGGACTTTGCAATATTTGATTGATCACAATTCTGTATATTCCATTTATTATAAAAGTTCCCAGGGAATTCATTAGAGGAATGTTTCCAATAAAAATAGTTTGTTCTTGCATATCCCTACTAGTTTTCCAAATTAATCCCGCGGATATATATAATTCAGAAGAATATGTAAGTGATTCATATACAGCATCTCCTTCTTTTATCAACGGTTCTACTAATTGATATGTTTCCACAAATAATTGAA |
| SNP103 | C/T | 23421 | ACATGAACAGCCATTTGATCCCCATCAAAGTCTGCATTGAATCCCTTACAAACTAATGGATGTAAACAAATAGCGCGGCCTTCCACTAAAATGGGTTGGAATGCCTGTATGCCTAATCTATGCAAAGTCGGCGCTCTATTCAGTAATACCGGATGCCCCTGCATAACTTCCTGAAGTATTTCCCATACAATTGGCTCTTTTTCCCGAATTTTACTCTTAGCAACTCCTATGTTCGAAGCAAGATGTTGTCTAATTAGACCACGAATTACAAATATCTGGAAAAGTTCTATTGCTATTTCGCGAGGCAATCCACATCGATGTAATGAAAGTGAGGGGCCTACGACAATGACAGAACGCCCCGAATAATCAACCCGTTTTCCAAGCATAGTCTCACGAAATCTTCCTTCTTTGCCTTCAATTACATCCGAAAACGACTTGTAAACCTTATTATGACCGTCTCTCATTGGTTGTCCGCGGATTCCATTATCAAGAAGTGTATC[C/T]ACGGCTTCTTGTACTAATTTTTCCTGACACATTACTAATTCTCCTGGCGTAGATCTACTTGTTGTTAATAGATCAATAAGAGTATTGTTCCGATAGATAACTCTTCTATAGAGTTCATTAATATCTGAACTCATTAGTTTACCCCCATCTATCTGAATGATCGGTCTCAACTCGGGAGGAAGAACTGGTAATAGACATAAAACCATCCATTCTGGTTCTATATTTGTTCGAATAAAATGCTTAGCTAATTCCACGCGTCGAACCAAAAAATCCTTTCTTCTTCCAACTTTTCGATCTTCCCATTCATTCCCCGTAGGCCCTTCTTCCCCTAATTCTTTCCATTCTACCGATGAATAATCTATAATAATTCGCAAATCTAGATCGGCTAATTGTTCTCGGATAGCACCAGCTCCTGTAGAAATTTCTCGATTTCGAAATGTATCGAAGCCTTGGGCAGTAAAAAAAAGTGGAATACTGTATTTCCAGGATTGAATTTCGTA |
| SNP104 | C/A | 19152 | CCAACTCTCTTTTCTAGGTTTATCGATATTGAATCAATTGAACGCACTTCTAACACTTGTTCTACTTTTGGAAGACCCTGCGTTATATCACCAGATCTCGACTTTTCATATATAAATGTAACTAATGTATCTCCTTCGTAAAGGATTTCTCCATAATGGCCATGAACGGTTGCTCCCGGAGTGGCCAAATAAGGCTTAGCGGATCTTATTACTACAGAGCCAATGTGAATAATTATAACTTGACCCGATTTTAGATGTGGTCCGTTTTTGGCCATACAGACATTTTCGCAAATAAACTGTCCCAGGCTAATTATTGTGAAGCTTTCTTCACAATAATTATGACAATAATTATGATGATAATTATGATGGAGAAAATACCAATTCAAATTGAATGGATTCAAAACACTGTTACTGCATGGATCGGGATTAACAACTCTCCCGTTTTCGTCCATTAAATAATATTTAAGTACTCGAAAAGTCTGTTTTAAATTGTCAAGTTT[C/A]AAATATTTAGTTACTGAGATCCGATTATGAGTTATTAAATTTAAATGGTAAAATAAATACAAATTCGCAATTTGAAGGGCTGTTCCTAAAGGGCCCAACGAATTTCTAATTGGAATTATAGGATCTCTTTTAATTGATTCTTTTATTACATTGTGATGTTTTACATCGTTGAAGGGATCCATTCGAAAACAATTAGATGATGACAAAATTATCAAAGATTGACATTCCTTATTTCTATTCAACAACGTACTAATAGTTCCTTGATTTTGTTTAAGTGATTGTTGAATCCTTGTCTTGAAATAGATGGGATAAAATGGATTAATATTGGTGTGATTTGCCCCATTATTAGAGATCGGCCCTGAACCCGATGGATCATTCCTTTTTCTACTGATATATGAAATATGGGATTTCACTAGGTTGATTCTTAAGAAATCACGAATCAGACCATTTGTGTTTACTTCAACAAAGGAAGCGCGGGCCTCTTCGATAGAAGAACTTTT |
| SNP105 | T/G | 18506 | TATAAATGATTGTTCTGATGTATCGTAGAAATTTTTTGAAATGCAAGAATAAAATAACTCTCTGTGGTGGAATAAAATATCTCTATCTCTAAATTCCCCCCCGAATAAATTATTCTTTTTGGTTTTCAAAGGAATGTTCTTATATTGCCTTGAACCTTGTACTAATCCTTTGAATCCGGTACCAACGGGTATCATACCGCCTAGAACAACGTTTTCTTTCAGGCCTTTCAACCAATCGATACGACCGCGGAGAGCGGCTTTTGCTAAAACACGAGCAGTTTCTTGAAAACTGGCCTCGGATATGAAACTTTGAGTATTCAGGGATGCTCTCGTTATTCCCAATAATATGGCTCGGTAACAGATGGCTTCTTCCAAAGCACGTCCCGTTCGTTCCGCTCGCAACAATCCAATTAGTTCTCCGGGTGAAAAAACATTAGACATTCCGTCTTCTGAAACCAATACTTTTGATGTTATTTGACGTACAATAATTTCTATATGCC[T/G]ATTATGGATCTGCACCCCTTGGGATCGATAAACCCTTTGGATCTTATTAACCAAAGAGATGCGACTTTGCACTATAGTTAGCTCAGTACCAATCAAGAATCCCCAAGGAATTCCAAGAATTCTTGGTATACATTCGTTCCAACCCCCAACTCTCTTTTCTAGGTTTATCGATATTGAATCAATTGAACGCACTTCTAACACTTGTTCTACTTTTGGAAGACCCTGCGTTATATCACCAGATCTCGACTTTTCATATATAAATGTAACTAATGTATCTCCTTCGTAAAGGATTTCTCCATAATGGCCATGAACGGTTGCTCCCGGAGTGGCCAAATAAGGCTTAGCGGATCTTATTACTACAGAGCCAATGTGAATAATTATAACTTGACCCGATTTTAGATGTGGTCCGTTTTTGGCCATACAGACATTTTCGCAAATAAACTGTCCCAGGCTAATTATTGTGAAGCTTTCTTCACAATAATTATGACAATAATTATGAT |
| SNP106 | T/G | 10905 | ATTATAATTATATTATAATTATTAATATTATTATTCTAATTATTAATATTATTATTATATTATAATATTATATAATATTATATAAATTTTTTAATATTAATATTATATAAAATATTATATAAATTTTTTAATTTTATATTTTATATTATATAAATAGCTAACTATATATTAACAATATATAATAACCCTCTATTCTATATAGTTAGTTATTATAGAAGTTATTAAGTTTTTATATATTTCTATTATAACTAGTTAATATCTATTATAACTATATAATATAAAGTTATAGTATATAGTTATAATTTGTTCTATATAAATAGACTCTAGATAGTGTATATACTATCTTTATATTAATATACTATCTTTATATTATTAGAATATAAATTAATAAGAAACTAGAATAATGTTAGAAAAAGTAATTGTAAATTGTAATAAAAAAAATTGAAAATTCTAATAATCTAATAATAAATTCTAAAAATTAGAATATTTCATTCTAAAAT[T/G]AAATAATTCTAAAATGAATATAAAAGAATTAATCCATCATTTACATTTACATTTTTTACATTTCATTTTTAATTCAAAAAATAGGTAATTCCTAAAATTATCTTGCATATTCTTTTTTTTATGAACAAAAAATATGAAAGAAAAAAGAAAAAAATTGGAATGGAATAGAAAAGCGTCCATTGTCTAATGGATAGGACAGAGGTCTTCTAAACCTTTGGTATAGGTTCAAATCCTATTGGACGCAATTTTTTTTCATATAATATACATATTATAATATAATAATCTATATATAGATTATATAGATTTTATATAGATTCTAAAATATAAATATATAGATTATAAATTTATAAATTATAAAATAAAATTTTTATAATTAAAAGTTAAAGATTAAAAGTGATTAATTTCTTTATACTTATACCTGTAGTGATTTATTTCTTTAGACTTGTTCCTGAAGTAGAAAACGTTCCTTCTGTTCCTGAATAGCTTCTTTCAAAAGGGTT |
| SNP107 | T/A | 8826 | ATAGTATAAAGTATAAACTCTTAATTCAAATATTGAAGTTATTGTATAAATATAAACTATAAACGCGAGAATTCTGGATCACCCCATTTTTTTTCATTCTAAACTTTTTTTCCATTGCAGATCCTATTAGAATCCTTTTTGTAGTTATGAAAAAATATCTAATTTTCGGTATGAAACAAAATTTTTGGCAATTAAAGACTCGACTTTTATTACAAATGAATTTTGAATTTAGAAAAATTATTTTCTATTTCTAGAAACCGCTCCATGTCTTGGTGTTAAAATAGAATATGTGGTATAAAAATAGAGAATCTATTTTTTTTTCCAAACCAAAAAAAGATCTTGGAGATTTTATAATGCTTACTCTCAAACTCTTTGTTTACACAGTAGTGATATTCTTTGTTTCTCTATTCATCTTTGGATTTTTATCTAATGATCCAGGACGTAATCCCGGACGTGAAGAATAAAAAAATAAAATATATAAAATTAAAATAAAAAAAAAA[T/A]ATAAGGGTTTTTCTTGATTTTTCAATGTTCTTAGTATTTTACTATTCTACATTTTTAAATATTAAATAAAACAAAAAAGTTCGCTAAATTTAAAAGAAAAGAAAAAAATTCCAAGTCATCACCGGAACCGGAAAGAGAGGGATTCGAACCCTCGGTACGGATAACTCGTACAACGGATTAGCAATCCGACGCTTTAGTCCACTCAGCCATCTCTCCCGATTGAAAAAGGATACTTACTATGTTACATTACACAACAGGTAAGGCTTGAAAATAAAAAAAAAGCCTTTTTCCCTCTTTTCTTTATTTTATTACTTTTATTACTTTCAAAATTACTTTTTATATTTTTTTTATTCTTTTATTTTTATATGGAACTATTTTATTTAATTTAATATATTAATTTAATATATTAAATTAAATATATATTCTATAATTATATATTTAATTTGAATAATTATATATTCAATTTAAATGAAAATTTAAAATAAAATTCAAAATGTCTT |
| SNP108 | C/T | 83854 | TTGCCTATTGATAAATCCCTAACTTTAGGTCTTTTTTAAATTGATTCAATTGTAAAATAAAATATCATGATGTGTATGTCTATCTAGGGAATAGTCGCTTCAAAGTGAATTCTCCCTAGATAACATCTATCGGATTTATATTTTATTCTTTGGTAAAAAAATTAAATTGCGAAATTTTTTTCTATTTCTAGAATACCCAATATATGTTTTACATCGTCTATACGAAAATGTTCAATTTTCATAAGATCTTCTTGACTCTTATTCAAAAGGTCCGATAATGTATGTATATTGGATCTTTTGAGGCAATTATAGATCTTAGGAGTCAATTCTGATTGGTCAATAAAAACGTATTTTAATGTTATTTCGTTTTGATTTTTTCTTAATTTAGTCAATCTATCATAAAAGGTAAAAAGAGGTAAAGTAACCTTGTGTTGATTTTTTTCTAAATGGAAGTTTTCTTCTTCTGCATGTAGAAAAGGAATAAATAAATCAATCAAATT[C/T]CGGGAGGCTTCATGAAGTGCTTCTTTAGGAGTTAAACTCCCATTTGTCCATATTTCGAGAAAAAGTATCTCTTGCTTTTCATTCCCATTTCCATAAGAATGAACACTATGATTCGCATTTCGAACAGGCATAAATACAGCATCTATAGGATAACTTCCCTCGTGAAAGTTATTTGTCGGTTTTATACGATAGCCGCGATTTCTCTCGATTTGTAATCCAATACACAAATCAATTGGTTCCGTTAGGCTAGCGATATGCTGTGTATTATCAATGATTTCTACAAAAGGTGGTAAAATGATGTCTTGAGCAGTTATATATCCAGGACCCTTGACACAAATAGACGCGTCACAAGTTCCATACAAATTGCTTCTCAATACAATTTCTTTCAAATTCATTAAAATTTCATGTATTGATTCTTGAATACCTGCTATAGTCGAAAATTCGTGTGGTATTTTCTCAGATTTTGCACGTGTGATACATGTTCCTTCGATTTCTCCAAG |
| SNP109 | A/C | 36413 | CCTCCTCCATAGAAAAGTTTATTCGAAGTCATAACATAAGACATAGAAGATACTTTTTAAATATCTTTCTTTGATTCCAGGACACAAAATCAATTTATATTGCTATTTATAGGTATATAAGATTTATATATAATAAGATTGATATATCTATCAGATCATGGCTTCCTGTACCAAATATTTTGGTATCGATACATACAATATTTTTATTCCGACAATGTAATGTAGAATAGGTGTGAGAAAAATACTTTCATTTTTAAATCCTTTCATTTAAAGTTTTCTATTTATTTAATATTGTATTGAATTTAATAATAGGAAAATTCATTCTCTTTCTTCTTTTCTGACAGATATAAAGTCAAGTAAATAAAAAAACTATTCGAAGCGTCTTTCTTGCTTTGACCTACAAAATATTCTGGTTTTTTTTTTATTTATTTTATCTGATTTCTTTTTTATTTTATCTGAAGTAAAAAGAAATATAACAAAAATGGCAAAAAGAAAATATA[A/C]GAAAGTTGGCGAGAAAGAATAAAATATAAAATAATAAAATATAGGATACTGTAACAGTTTAATGCATATAGAAATTAGAAGAATACGTAAAAATATTGATTTTTAGAAATCTTACGAACAAGATCCAAGAATAAGATTAGTTTGATAGAATGAGAGAAATAAGTCTGAGGATCCACTAGTAAGGAGAGGGGGATCACTTGTTCCTTGAACAGTGCTTTTAAAAAATTCATCTATCTGATTGATTTGATGAGTCATAAAAAAATTCATGGTTCATATGGTTATTAAGACTATAAAAAAGAATAACCGAATTGAATTCATGAATTTACCTAAGTCAGGTTATGGACCGATAAAGAATTTTTTTCTTCGAAACCCATTAGAAATTAGAAAGGGCAGTGTACAAGAAATCAAATCATACATAAATGATAGAAGCTTCAAAGGCCCTGAAAATGCTATGAGGTGTTCGGAAATGGTTGAAGTAGTTGAATAGGAGGATCACTATG |
| SNP110 | C/A | 85588 | TTTGTACATCAGATCCTTTAGAGCGTTTCCGCTTAGCAAGATTATCCTTGTCTTTGTTTATGTCTCGGGTTAAAGCAAATTACTATAATTCGCCCCCGTCTACGTATCAGTCGACATTTTTCACAAATTTTACGAACAGAAGCTCTTATTTTCATATTTGCCGTCCCTTAAATTTTGAATATACATACTTTTTTTGAAGAAAATAAGTTTCTTTAAACTTTGAAATCGCGAATTGTATCCCTTGAAAGTGATGAAAGTGAAAGGACTTTTTAAGTTGAAAAAAAAAAACTGACTAATCATTCAAATCTTTGTTTGTTACGGAGTCTATAAATTAGAAGTGCTCTGGTCGAATTCTAAATACTTAACACTATTTCTGAGCAGTATAGGTATAAAAAAACTATGTTGGATCCTTCCTGAAGCATAACCTAAAACCAGATCTTCATTATCTAAAGGAACTTGGAACATACTATTGAAAAAGTGATTCAGAAATTTAACCTTCC[C/A]AAATTGAATCCATTTTTTGTTCTTTCATTCCATCTAAAATCCCCTTCAAGTATCAATTAATGTAATAGGAATGATATTAGAAACCTCTTCTTTCTCTTTTTTTTTTTTTCACAAATAAGAAGTTGGGATACAATTCGGATATCCGAAAGATTACCATATATAACACAAGATTTCTCCACCGATTCTTTCTAGTCGAGCTTCTCGGTCGGTCATTATACCCCGAGAAGTAGAAAGAATTACAATACCCATCCCGCCCAAAATTCTAGGAATTTTTTGATAGTTAGAATAGATTCGTAGACCAGGTCGGCTAATCCGTTTTAAATTTAGACTAGTTCTATAGGGTCCCTTCCTCTTCCTTCTATGTCGTAGGGTTAAAACCAAAAATTTTTTGTTGCCTTCCTGGTGTTTCCTGACATTTTCAATAAAACCTTCTCGTAAAAGTATTTTAATAATGTTTTCAGTGATGTTAGTGAATGCTATTCGAACGGTTCCTTTTCTAT |
| SNP111 | C/T | 124154 | TAAGAAAAAAAGTGCAGCGCCAATAAACCCATGTGATATTATTTGTAAAATGGCTCCATTGAGTCCCATATCACTTATAGAGCAAATTCCTATAATTATGAAACCCATATGAGATACAGAAGAATAGGCTATTCTTTTTTTTAAATTTCGTTGACCAGGAGATGTTGAAGCTGCATAGATTATTTGCATGACGCCTACTATTATCAACCAGGGAGAAAAGATAGAATGAGCATGAGGTAATAATTCCATATTGATTCGAACCAACCCATACGCCCCCATTTTTAATAAGATTCCGGCTAGAAGCATACAAGTACTGTAATGTGCTTCCCCATGGGTGTCTGGTAACCATATATGTAAGGGTATAATCGGTAATTTGACAGCAAAAGCAATAAGAAATCCAATATAAAAAAATATTTCTAGCGCCACAGGATATGATTGATTGGCTGATGTTTCAAAATTGAATCTTGGTTCATTGGAACCATATAAAGCGATACCCAAAG[C/T]TCCCATTAATAAAAAAACGGAACCCCCGGCAGTATACAAAATAAACTTTGTAGCTGAATACAGACGTTTCTTTCCCCCCCACATGGATAGAAGTAGATAAATGGGAATTAATTCTAACTCCCACATGATAAAAAAAAGCAAAAGATCTTGAGAAGAAAATAATCCTATTTGACCACTATACATTGCTAACATCAGAAAATTAAATAAGCGGGAATCACGAGTAATTGGCCAAGCCGCTAAAGTAGCTAAAGTGGTGATAAATCCTGTCAGTAAAATAGGGCTTAAAGAAAATCCATCTATTCCCAATCTCCAGTAAAAATCAAAAAATTGGATCCATTTATAATCTTCTGTTAATTGGATTAATGGGTCGTCCAATTGGAAATAATAAGAGAACGCATAAGTCATTAAAAGGAGTTCTAAAATACATATAAACAAAGTATACCACCTAATTACCTTATTTCCTCTATGGGGGAAAAAGAAAATTAAGGAACCCGCAGATA |
| SNP112 | G/A | 55604 | ACTGATTATTATCAAAATGATTCGAACTTTTTCAAAGACCCAACATGCATTTTTTTGCATTGGGCTCGTTCATTAACTGATATAAATAATCGGTTAGTCTACCATAATTCTCTTGGCAGAAAGATAAGAGGATGGCTCCACGTGCTCTGATTTATGGATTCCGATCCGAGAGCACTACCAAAGTGTTTCAAAGAAGGGTTATCCTGACGTAGGTCTGCTTTTGGCTTAGATTAACCTAAGTTAAATGGAATCTCCATCGCCCCGCTTCTGCTTAAAGAATCAAATATGAAACTTCATACACCTTAAAGTTCATAGGATAGGACGAAAAGAGAATTTTTTGAGGTCCTTATACTCATTATGCCTAGCATTGAATAGACTGGGTATTCACCTTATCAAGGTCTTAAATCAATGATGGGTTCTATTGGGTGTCTGCAAAGGCACTTAAATTGGACCGAACCCTTTGTGTCAGGCTATTGTTCTCCTGTTCCCTAAAAGTAATG[G/A]AGTAAGACATCGACTTCTCAATAAGTCTTTTGATTACATGATGGACTCCCTTGAAAAAAAAAGCATTGGCGCGCGTGTAAACGAGGTGCTCTACCTAACTGAGCTATAGCCCTTGTGTTTGTGATACATATTTTATCATGTCAATAATTTCTTGTCAAGATGAATATTATATGATCCAGCATCCTATTCTATTTCTTTGATCTCTTTGATCGGTATTGCTTATTTTAATCGGTATTGCTTATAAATAATATTCCATTTATAATTAAAATTAATCGTCGATGCGACGGATTATATTTCTTTATCTTTGGGATGATAAATGACCTACTTAACTCAGTGGTTAGAGTATTGCTTTCATACGGCGGGAGTCATTGGTTCAAATCCAATAGTAGGTACAAATTATTAGATATCAGAATCAATGCTATCTAATAAATTTTTTTACCCACCTTAGTTTAGTGATTTTTGATCTTTTTCGTTCCATTCTATTTCATTTTTGAATTGAA |
